# Cu-catalyzed mild and efficient oxidation of THβCs using air: application in practical total syntheses of perlolyrine and flazin†

**DOI:** 10.1039/c7ra13434g

**Published:** 2018-02-12

**Authors:** Bo Zheng, Tien Ha Trieu, Tian-Zhuo Meng, Xia Lu, Jing Dong, Qiang Zhang, Xiao-Xin Shi

**Affiliations:** Shanghai Key Laboratory of Chemical Biology, School of Pharmacy, East China University of Science and Technology 130 Mei-Long Road Shanghai 200237 P. R. China xxshi@ecust.edu.cn

## Abstract

A mild, efficient and environmentally benign method for synthesis of aromatic β-carbolines *via* Cu(ii)-catalyzed oxidation of 1,2,3,4-tetrahydro-β-carbolines (THβCs) was developed, in which air (O_2_) was used as the clean oxidant. This method has advantages such as environmentally friendliness, mildness, very good tolerance of functional groups, high yielding and easy experiment operation. In addition, this new methodology was successfully applied in the efficient and practical total syntheses of β-carboline alkaloids perlolyrine and flazin.

## Abstract 2

A mild, efficient and ecofriendly method for synthesis of β-carbolines *via* Cu-catalyzed aerobic oxidation of 1,2,3,4-tetrahydro-β-carbolines (THβCs) was developed. In addition, this method was successfully applied in the practical total syntheses of perlolyrine and flazin.

## Graphical abstract

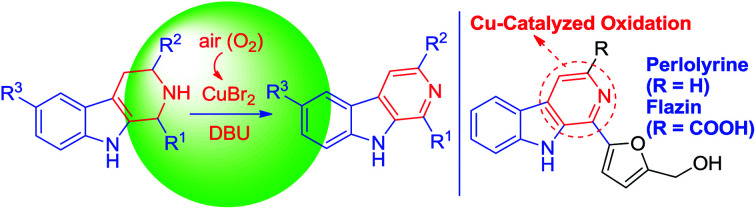

## Introduction

The aromatic β-carboline skeleton is ubiquitous in many alkaloids,^1^ bioactive congeners,^2^ agrochemicals^3^ and functional materials.^4^ In view of the significance of β-carbolines in drug discovery, agricultural chemistry and material science, development of practical, efficient and environmentally benign methods for syntheses of β-carbolines is of considerable interest and has attracted much attention from chemists.^5^

Since 1,2,3,4-tetrahydro-β-carbolines (THβCs) can be readily and efficiently prepared *via* the Pictet–Spengler reaction,^6^ so the aromatization of THβCs *via* dehydrogenation or oxidation is an easy and good method to obtain β-carbolines. Dehydrogenation of THβCs usually employed precious metallic catalysts including Pd,^7^ Pt^8^ and complexes of Ru^9^ and Ir.^10^ Oxidation of THβCs normally needed stoichiometric strong oxidants such as KMnO_4_,^11^ MnO_2_,^12^ SeO_2_,^13^ DDQ,^14^ TCCA^15^ and IBX.^16^ However, uses of expensive metallic catalysts and hazardous strong oxidants are sometimes lack of cheapness, practicality and environmental friendliness, therefore novel practical, efficient and environmentally benign methods for the conversion of THβCs to β-carbolines are highly desirable.

Oxygen is an important component of air, which is one of the most abundant resources on the earth, so the air has been used as a clean and eco-friendly oxidant for various oxidation reactions in recent decades.^17^ On the other hand, copper is one of the most abundant metals on the earth's crust, thus copper salts are normally very cheap, and moreover copper is a low toxic transition metal, so an increasing amount of copper-catalyzed reactions have been developed recently.^18^ Herein, we describe a new practical and very mild Cu(ii)-catalyzed oxidative conversion of THβCs to β-carbolines using air as the clean oxidant.

## Results and discussion

At first, we attempted to find out the optimized reaction conditions for the Cu-catalyzed oxidative conversion of THβCs 1 to β-carbolines 2. With the conversion 1-phenyl-THβC 1a to 1-phenyl-β-carboline 2a as the model reaction, we tried the reaction under various conditions, and results are summarized in Table 1. As can be seen from Table 1, various copper reagents have been tested as catalysts (Table 1, Entries 2–11), and it was found that cupric bromide is the best catalyst for the reaction. When 0.2 equivalents of CuBr_2_ was used as the catalyst, the aerobic oxidation of compound 1a took place smoothly at room temperature in DMSO in the presence of 2 equivalents of 1,8-diazabicyclo[5,4,0]undec-7-ene (DBU) to afford the desired compound 2a in high yield (Entry 2). Several amines such as DBU, 1,5-diazabicyclo[4,3,0]non-5-ene (DBN), 4-dimethyl-aminopyridine (DMAP), pyridine and trimethylamine have been tested as bases (Entries 2 and 12–15), DBU was found to be the most appropriate base for the reaction. Amount of DBU has significant effect on the reaction (Entries 2 and 16–19), almost no desired product 2a could be obtained in the absence of DBU (Entry 16); and the reaction was hard to be complete even for a longer time, if less than 2.0 equivalents of DBU were used (Entries 17–19). Several other solvents such as *N*,*N*-dimethylformamide (DMF), acetonitrile, ethanol, tetrahydrofuran (THF), dichloromethane, ethyl acetate and acetone have also been examined (Entries 20–26); DMSO is obviously better than them. Additionally, when the reaction temperature was elevated, the reaction velocity increased, but the yield of desired product 2a obviously decreased (Entries 27–29).

**Table 1:** Optimization of reaction conditions for the Cu-catalyzed aerobic oxidative conversion of 1-phenyl-THβC 1a to 1-phenyl-β-carboline 2a^a^

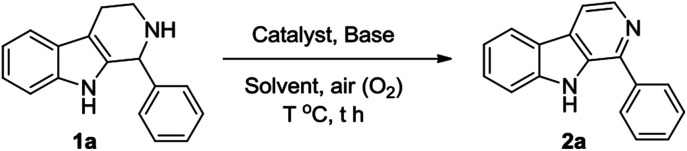						
Entry	Catalyst^b^	Base^c^	Solvent	*T* (°C)	*t* (h)	Yield^d^ (%)
1	None	DBU^e^	DMSO^f^	25	18	0
2	CuBr_2_	DBU	DMSO	25	18	95
3	CuCl_2_	DBU	DMSO	25	18	90
4	Cu(OAc)_2_	DBU	DMSO	25	18	86
5	CuBr	DBU	DMSO	25	18	85
6	CuCl	DBU	DMSO	25	18	84
7	CuI	DBU	DMSO	25	18	82
8	CuSO_4_	DBU	DMSO	25	24	<5
9	CuCO_3_	DBU	DMSO	25	24	11
10	CuO	DBU	DMSO	25	24	<5
11	Cu	DBU	DMSO	25	24	<5
12	CuBr_2_	DBN^g^	DMSO	25	20	88
13	CuBr_2_	DMAP^h^	DMSO	25	20	19
14	CuBr_2_	Py	DMSO	25	20	12
15	CuBr_2_	Et_3_N	DMSO	25	20	10
16	CuBr_2_	None	DMSO	25	18	0
17	CuBr_2_	DBU (0.5)^i^	DMSO	25	60	75
18	CuBr_2_	DBU (1.0)^j^	DMSO	25	30	83
19	CuBr_2_	DBU (1.5)^k^	DMSO	25	25	92
20	CuBr_2_	DBU	DMF^l^	25	24	82
21	CuBr_2_	DBU	CH_3_CN	25	24	65
22	CuBr_2_	DBU	EtOH	25	24	23
23	CuBr_2_	DBU	THF^m^	25	24	30
24	CuBr_2_	DBU	CH_2_Cl_2_	25	24	27
25	CuBr_2_	DBU	EtOAc	25	24	12
26	CuBr_2_	DBU	Me_2_CO	25	24	14
27	CuBr_2_	DBU	DMSO	45	13	90
28	CuBr_2_	DBU	DMSO	65	9	84
29	CuBr_2_	DBU	DMSO	85	6	76

a All reactions were performed at *T* °C for *t* hours under an atmosphere of air.

b 20% (mol%) of the catalyst was used.

c 2.0 equivalents of the base were used unless otherwise indicated.

d Isolated yields.

e 1,8-Diazabicyclo[5,4,0]undec-7-ene.

f Dimethyl sulfoxide.

g 1,5-Diazabicyclo[4,3,0]non-5-ene.

h 4-Dimethylaminopyridine.

i 0.5 equivalent of DBU was used.

j 1.0 equivalent of DBU was used.

k 1.5 equivalents of DBU was used.

l *N*,*N*-Dimethylformamide.

m Tetrahydrofuran.

Subsequently, we attempted the CuBr_2_-catalyzed oxidative conversion of variously substituted THβCs 1 to β-carboline 2 in DMSO with DBU as the base, and the results are summarized in Table 2. The scope of the reaction is very wide; almost all of the tested THβCs could be smoothly converted to β-carbolines in good to excellent yields. All the conversions were performed under the standard reaction conditions (see footnote of the Table 2), and a total of twenty-four variously substituted β-carbolines 2a–2x were obtained in 78–96% yields.

**Table 2:** CuBr_2_-catalyzed oxidation of variously substituted THβCs 1 β-carbolines 2 using air as the clean oxidant^a^

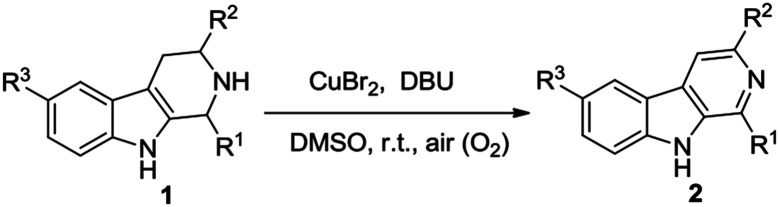
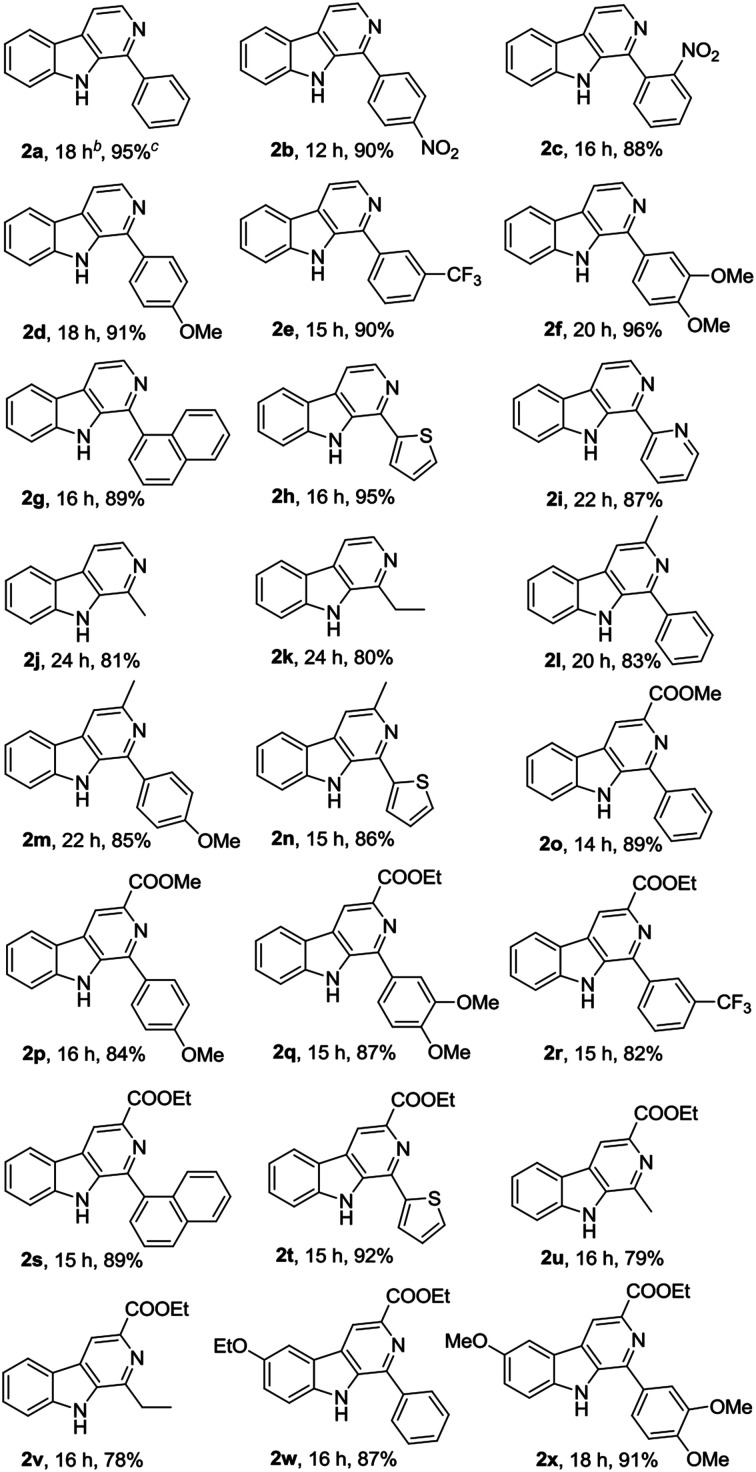

a Reaction conditions: THβCs 1 (2 mmol), CuBr_2_ (0.4 mmol), DBU (4 mmol), DMSO (6 mL), stirring at room temperature (25 °C) under an atmosphere of air.

b Reaction time.

c Isolated yields.

**Scheme 1:**
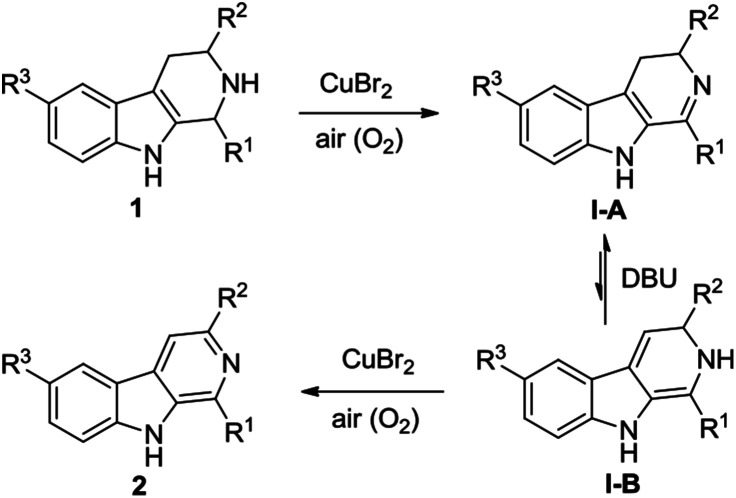
Possible mechanism for the CuBr_2_-catalyzed oxidative conversion of THβCs 1 to β-carbolines 2.

A possible mechanism for the CuBr_2_-catalyzed oxidative conversion of THβCs 1 to β-carbolines 2 is proposed in Scheme 1. As can be seen from Scheme 1, THβCs 1 would first undergo CuBr_2_-catalyzed aerobic oxidation of C–N single bond to C=N double bond to produce 3,4-dihydro-β-carbolines I-A according to Adimurthy's reports.^19^ 3,4-Dihydro-β-carbolines I-A would then undergo DBU-promoted reversible tautomerization^20^ to form unstable enamine intermediates I-B, which would also undergo the CuBr_2_-catalyzed aerobic oxidation of C–N single bond to afford β-carbolines 2. Actually, dihydro-β-carbolines I-A could be isolated if the reaction was stopped in the midway. For example, when the CuBr_2_-catalyzed oxidative conversion of 1-phenyl-THβC 1a to 1-phenyl-β-carboline 2a was stopped at 8 h, 1-phenyl-3,4-dihydro-β-carboline was obtained in 36% yield.

To showcase the synthetic utility of the above-described method for the CuBr_2_-catalyzed oxidative conversion of THβCs 1 to β-carbolines 2, we have successively applied the methodology to the efficient and practical total syntheses of two β-carboline alkaloids perlolyrine 3 and flazin 4.

**Scheme 2:**
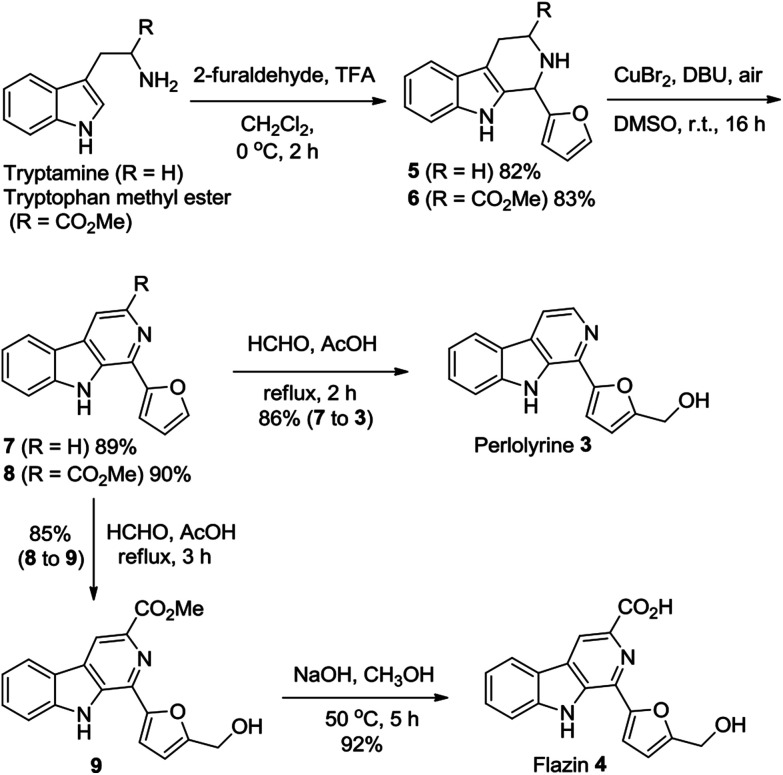
Novel concise total syntheses of β-carboline alkaloids perlolyrine 3 and flazin 4.

Perlolyrine 3 and flazin 4 are both strongly fluorescent 1-furanyl-β-carboline alkaloids. These two particular alkaloids are widespread in nature, and have been isolated from plants, bacteria, Japanese sake and soy sauce.^1*h*,3*b*,21^ Several total syntheses of perlolyrine 3 and flazin 4 have been reported hitherto,^22^ we herein report novel practical total syntheses of them, which are depicted in Scheme 2. Pictet–Spengler reaction of tryptamine and tryptophan methyl ester with 2-furaldehyde first gave THβCs 5 and 6 in 82% and 83% yields, respectively. CuBr_2_-catalyzed oxidation of compounds 5 and 6 with air then furnished β-carbolines 7 and 8 in 89% and 90% yields, respectively. Next, hydroxymethylation of compounds 7 and 8 with HCHO in AcOH produced perlolyrine 3 and compound 9 in 86% and 85% yields, respectively. Hydrolysis of the ester 9 afforded flazin 4 in 92% yield. Thus, perlolyrine 3 was synthesized from tryptamine in 3 steps in 63% overall yield; and flazin 4 was synthesized from tryptophan methyl ester in 4 steps in 58% overall yield.

## Conclusions

In conclusion, we have developed a novel method for the CuBr_2_-catalyzed oxidative conversion of THβCs to β-carbolines. This method has some advantages as follows: (a) the process was carried out at room temperature with air as the clean oxidant, so it is very mild and environmentally benign; (b) CuBr_2_ as the catalyst is inexpensive and low-toxic; (c) all products were obtained in good to excellent yields; (d) the reaction has very good tolerance of functional groups, it seemed to be applicable to all the tested substrates; (e) experiment operation is quite easy. In addition, novel practical total syntheses of β-carboline alkaloids perlolyrine and flazin were performed with the above-described CuBr_2_-catalyzed mild oxidation of THβCs as the key step.

## Experimental

### General

^1^H NMR and ^13^C NMR spectra were acquired on a Bruker AM 400 instrument, chemical shifts are given on the *δ* scale as parts per million (ppm) with tetramethylsilane (TMS) as the internal standard. IR spectra were recorded on a Nicolet Magna IR-550 instrument. Mass spectra were performed with a HP1100 LC-MS spectrometer. Melting points were measured on a Mei-TEMP II melting point apparatus. Column chromatography was performed on silica gel (Qingdao Chemical Factory). All reagents and solvents were analytically pure, and were used as such as received from the chemical suppliers.

#### General procedure for the preparation of various THβCs 1

1-Substituted tryptamine (3 mmol) was dissolved in CH_2_Cl_2_ (10 mL), an aldehyde (3.3 mmol) was added. The resulting solution was cooled to 0 °C, TFA (0.686 mg, 6.016 mmol) was added. The mixture was then stirred at 0–5 °C for 2–6 h. After the reaction was complete (checked by TLC, eluent: EtOAc/CH_2_Cl_2_ = 1 : 4), solvent was removed under vacuum, the residue was then partitioned between EtOAc (30 mL) and an aqueous solution of K_2_CO_3_ (10% w/w, 20 mL). Two phases were separated, and the aqueous phase was extracted again with EtOAc (20 mL). Organic extracts were combined, and dried over anhydrous MgSO_4_. Evaporation of solvent under vacuum gave crude product, which was purified by flash chromatography (eluent: MeOH/CH_2_Cl_2_ = 1 : 20–1 : 10) to afford THβCs 1a–1x in 70–95% yields.

#### General procedure for the CuBr_2_-catalyzed oxidative conversion of THβCs 1 to β-carbolines 2

THβCs 1 (2 mmol) was dissolved in DMSO (5 mL). DBU (609 mg, 4.000 mmol) and anhydrous CuBr_2_ (89.5 mg, 0.401 mmol) were added. The resulting solution was then stirred under air at room temperature (around 25 °C) for 12–24 h (see Table 2). After the reaction was complete (checked by TLC, EtOAc/CH_2_Cl_2_ = 1 : 1 to 1 : 4), the reaction solution was poured into the mixed solution of EtOAc (30 mL) and aqueous ammonia (5% w/w, 25 mL). After the mixture was stirred for 5 minutes, two phases were separated. The aqueous layer was extracted again with EtOAc (20 mL). The organic extracts were combined and washed with brine (10 mL). The organic solution was dried over anhydrous MgSO_4_, and then was concentrated under vacuum to give the crude product, which was purified by flash chromatography (eluent: EtOAc/CH_2_Cl_2_ = 1 : 2 to 1 : 15) to afford pure β-carbolines 2 in 78–96% yield as indicated in the Table 2. Characterization data of the β-carbolines 2a–2x are given in the ESI† of this paper.

### Total syntheses of perlolyrine 3 and flazin 4

#### 1-(Furan-2-yl)-1,2,3,4-tetrahydro-9*H*-pyrido[3,4-*b*]indole 5

A mixture of tryptamine (1.604 g, 10.01 mmol), 2-furaldehyde (1.076 g, 11.20 mmol) and CH_2_Cl_2_ (15 mL) was stirred and cooled to 0 °C by an ice-bath. TFA (2.285 g, 20.04 mmol) was slowly added, and then the reaction mixture was further stirred at 0 °C for 2 h. After the reaction was complete (checked by TLC, eluent: EtOAc/CH_2_Cl_2_ = 1 : 3), ethyl acetate (60 mL) and an aqueous solution of K_2_CO_3_ (10% w/w, 30 mL) were added. The mixture was vigorously stirred for 5 min, and then two phases were separated. Organic phase was extracted again with ethyl acetate (20 mL). Organic extracts were combined and dried over anhydrous MgSO_4_, and then was concentrated under vacuum to give the crude product, which was purified by flash chromatography (eluent: MeOH/CH_2_Cl_2_ = 1 : 10) to afford pure compound 5 (1.957 g, 8.213 mmol) in 82% yield as white solid, mp 138–139 °C. ^1^H NMR (CDCl_3_, 400 MHz) *δ* 8.17 (s, 1H, N*H* in indole), 7.94 (s, 1H), 7.64 (d, *J* = 7.8 Hz, 1H), 7.48 (d, *J* = 3.2 Hz, 1H), 7.32 (d, *J* = 8.0 Hz, 1H), 7.18 (dd, *J*_1_ = 8.0 Hz, *J*_2_ = 7.9 Hz, 1H), 7.11 (dd, *J*_1_ = 7.8 Hz, *J*_2_ = 7.9 Hz, 1H), 6.96 (brs, 1H, N*H*), 6.66 (d, *J* = 3.7 Hz, 1H), 6.44 (dd, *J*_1_ = 3.7 Hz, *J*_2_ = 3.2 Hz, 1H), 3.90 (t, *J* = 7.3 Hz, 2H), 3.17 (t, *J* = 7.3 Hz, 2H). ^13^C NMR (CDCl_3_, 100 MHz) *δ* 154.51, 142.52, 135.87, 131.84, 127.23, 121.91, 119.37, 118.34, 111.04, 110.35, 109.94, 107.76, 50.73, 41.46, 22.31. HRMS (ESI) *m*/*z* calcd for C_15_H_14_N_2_ONa [M + Na]^+^: 261.1004, found: 261.1008. IR (KBr, film) 3393, 3145, 2922, 2846, 1450, 1298, 1141, 1010, 739 cm^−1^.

#### Methyl1-(furan-2-yl)-1,2,3,4-tetrahydro-9*H*-pyrido[3,4-*b*]indole-3-carboxylate 6

Compound 6 was prepared from tryptophan methyl ester and 2-furaldehyde according to the same procedure as above for compound 5. Compound 6 was obtained as an epimeric mixture of *cis* and *trans* isomers (*cis*/*trans* = 3 : 5) in a combined yield of 83% as offwhite solid. Two epimers can be separated by chromatography, but herein the epimeric mixture was used as such for the next step. Data for *cis* epimer: white solid, mp 141–142 °C. ^1^H NMR (CDCl_3_, 400 MHz) *δ* 7.98 (s, 1H, N*H* in indole), 7.50 (d, *J* = 7.6 Hz, 1H), 7.41 (d, *J* = 3.5 Hz, 1H), 7.25 (d, *J* = 7.8 Hz, 1H), 7.15 (dd, *J*_1_ = 7.6 Hz, *J*_2_ = 7.7 Hz, 1H), 7.11 (dd, *J*_1_ = 7.7 Hz, *J*_2_ = 7.8 Hz, 1H), 6.32–6.36 (m, 2H), 5.38 (s, 1H), 3.91 (dd, *J*_1_ = 9.8 Hz, *J*_2_ = 4.5 Hz, 1H), 3.79 (s, 3H), 3.17 (dd, *J*_1_ = 15.0 Hz, *J*_2_ = 4.5 Hz, 1H), 2.95 (dd, *J*_1_ = 15.0 Hz, *J*_2_ = 9.8 Hz, 1H), 2.44 (s, 1H, N*H*). ^13^C NMR (CDCl_3_, 100 MHz) *δ* 173.06, 153.02, 142.85, 136.03, 131.79, 127.01, 122.18, 119.68, 118.28, 111.07, 110.48, 108.61, 107.78, 56.51, 52.33, 51.64, 25.46. HRMS (ESI) *m*/*z* calcd for C_17_H_16_N_2_O_3_Na [M + Na]^+^: 319.1059, found: 319.1057. IR (KBr, film) 3393, 2950, 1735, 1436, 1351, 1271, 1218, 1174, 1141, 1009, 740 cm^−1^. Data for *trans* epimer: white solid, mp 145–146 °C. ^1^H NMR (CDCl_3_, 400 MHz) *δ* 8.30 (s, 1H, N*H* in indole), 7.49 (d, *J* = 7.5 Hz, 1H), 7.34 (d, *J* = 3.4 Hz, 1H), 7.16 (d, *J* = 7.8 Hz, 1H), 7.11 (dd, *J*_1_ = 7.5 Hz, *J*_2_ = 7.6 Hz, 1H), 7.06 (dd, *J*_1_ = 7.6 Hz, *J*_2_ = 7.8 Hz, 1H), 6.23 (dd, *J*_1_ = 4.2 Hz, *J*_2_ = 3.4 Hz, 1H), 5.96 (d, *J* = 4.2 Hz, 1H), 5.25 (s, 1H), 3.88 (dd, *J*_1_ = 9.5 Hz, *J*_2_ = 4.7 Hz, 1H), 3.69 (s, 3H), 3.13 (dd, *J*_1_ = 15.2 Hz, *J*_2_ = 4.7 Hz, 1H), 2.88 (*J*_1_ = 15.2 Hz, *J*_2_ = 9.5 Hz, 1H), 2.59 (brs, 1H, N*H*). ^13^C NMR (CDCl_3_, 100 MHz) *δ* 173.82, 154.40, 142.67, 136.26, 130.97, 126.78, 122.14, 119.49, 118.27, 111.20, 110.28, 108.65, 108.14, 52.24, 52.23, 49.10, 25.17. HRMS (ESI) *m*/*z* calcd for C_17_H_16_N_2_O_3_Na [M + Na]^+^: 319.1059, found: 319.1055. IR (KBr, film) 3395, 2945, 1736, 1435, 1352, 1271, 1218, 1175, 738 cm^−1^.

#### 1-(Furan-2-yl)-9*H*-pyrido[3,4-*b*]indole 7

Compound 5 (0.962 g, 4.037 mmol) was dissolved in DMSO (8 mL), DBU (1.225 g, 8.047 mmol) and anhydrous CuBr_2_ (0.179 g, 0.801 mmol) were added. The resulting solution was then stirred under air at 25 °C for 16 h. After the reaction was complete (checked by TLC, eluent: EtOAc/CH_2_Cl_2_ = 1 : 1), the reaction solution was poured into the mixed solution of EtOAc (50 mL) and aqueous ammonia (5% w/w, 20 mL). After the mixture was stirred for 5 minutes, two phases were separated. The aqueous layer was extracted again with EtOAc (20 mL). The organic extracts were combined and washed with brine (10 mL). The organic solution was dried over anhydrous MgSO_4_, and then was concentrated under vacuum to give the crude product, which was purified by flash chromatography (eluent: EtOAc/CH_2_Cl_2_ = 1 : 3) to afford pure compound 7 (0.842 g, 3.594 mmol) in 89% yield as white solid, mp 166–167 °C. ^1^H NMR (DMSO-*d*_6_, 400 MHz) *δ* 11.50 (s, 1H, N*H* in indole), 8.38 (d, *J* = 5.2 Hz, 1H), 8.25 (d, *J* = 7.8 Hz, 1H), 8.08 (d, *J* = 5.2 Hz, 1H), 7.99 (d, *J* = 1.6 Hz, 1H), 7.77 (d, *J* = 8.2 Hz, 1H), 7.58 (dd, *J*_1_ = 7.6 Hz, *J*_2_ = 7.8 Hz, 1H), 7.23–7.32 (m, 2H), 6.79 (dd, *J*_1_ = 3.5 Hz, *J*_2_ = 3.2 Hz, 1H). ^13^C NMR (CDCl_3_, 100 MHz) *δ* 155.09, 143.34, 141.09, 139.47, 134.09, 131.97, 130.83, 129.17, 122.25, 121.87, 120.72, 114.26, 112.92, 112.25, 109.32. HRMS (ESI) *m*/*z* calcd for C_15_H_11_N_2_O [M + H]^+^: 235.0871, found: 235.0872. IR (KBr, film) 3458, 2998, 2920, 1624, 1557, 1493, 1424, 1377, 1235, 997, 754, 459 cm^−1^.

#### Methyl1-(furan-2-yl)-9*H*-pyrido[3,4-*b*]indole-3-carboxylate 8

Compound 8 was prepared from compound 6 according to the same procedure as above for compound 7. Compound 8 was obtained in 90% yield as white solid, mp 168–169 °C. ^1^H NMR (DMSO-*d*_6_, 400 MHz) *δ* 11.93 (s, 1H, N*H* in indole), 8.90 (s, 1H), 8.44 (d, *J* = 8.0 Hz, 1H), 8.06 (d, *J* = 3.3 Hz, 1H), 7.84 (d, *J* = 8.2 Hz, 1H), 7.65 (dd, *J*_1_ = 8.0 Hz, *J*_2_ = 8.1 Hz, 1H), 7.32–7.40 (m, 2H), 6.85 (dd, *J*_1_ = 3.3 Hz, *J*_2_ = 3.7 Hz, 1H), 3.96 (s, 3H). ^13^C NMR (DMSO-*d*_6_, 100 MHz) *δ* 165.76, 152.11, 144.23, 141.48, 136.37, 132.76, 132.11, 129.51, 128.79, 121.90, 120.82, 120.48, 116.41, 112.97, 112.34, 109.92, 52.07. HRMS (ESI) *m*/*z* calcd for C_17_H_13_N_2_O_3_ [M + H]^+^: 293.0926, found: 293.0922. IR (KBr, film) 3440, 3060, 2951, 1702, 1624, 1493, 1430, 1368, 1346, 1260, 738, 494 cm^−1^.

#### 1-[5-(Hydroxymethyl)furan-2-yl]-9*H*-pyrido[3,4-*b*]indole (perlolyrine 3)

Compound 7 (0.470 g, 2.006 mmol) was dissolved in acetic acid (3 mL). An aqueous solution of formaldehyde (37% w/w, 1 mL) was added. The resulting solution was then heated and stirred at reflux for 2 h. After the reaction was complete (checked by TLC, eluent: EtOAc/CH_2_Cl_2_ = 1 : 3), the solvent was removed by vacuum distillation to give a viscous residue, which was partitioned between ethyl acetate (30 mL) and an aqueous solution of potassium carbonate (10% w/v, 20 mL). Two phases were separated, and aqueous phase was extracted again with ethyl acetate (20 mL). The organic extracts were combined and washed twice with brine (2 × 10 mL). After having been dried with anhydrous MgSO_4_, the organic solution was concentrated under vacuum to give crude solid, which was purified by flash chromatography (eluent: MeOH/CH_2_Cl_2_ = 1 : 10) to give pure perlolyrine 3 (0.457 g, 1.729 mmol) in 86% yield as pale yellow solid, mp 165–166 °C. ^1^H NMR (DMSO-*d*_6_, 400 MHz) *δ* 11.24 (s, 1H, N*H* in indole), 8.38 (d, *J* = 5.1 Hz, 1H), 8.27 (d, *J* = 7.8 Hz, 1H), 8.08 (d, *J* = 5.1 Hz, 1H), 7.78 (d, *J* = 8.2 Hz, 1H), 7.61 (dd, *J*_1_ = 7.8 Hz, *J*_2_ = 8.0 Hz, 1H), 7.29 (dd, *J*_1_ = 8.0 Hz, *J*_2_ = 8.2 Hz, 1H), 7.23 (d, *J* = 3.3 Hz, 1H), 6.60 (d, *J* = 3.3 Hz, 1H), 5.50 (t, *J* = 6.0 Hz, 1H, O*H*), 4.68 (d, *J* = 6.0 Hz, 2H). ^13^C NMR (DMSO-*d*_6_, 100 MHz) *δ* 156.73, 152.14, 140.92, 138.16, 133.14, 130.47, 129.42, 128.39, 121.59, 120.61, 119.68, 113.60, 112.40, 109.62, 109.04, 55.95. HRMS (ESI) calcd for C_16_H_13_N_2_O_2_ [M + H]^+^: 265.0977, found: 265.0974. IR (KBr, film) 3370, 2920, 1629, 1568, 1426, 1317, 1234, 1014, 799, 743, 634 cm^−1^.

#### Methyl1-(5-(hydroxymethyl)furan-2-yl)-9*H*-pyrido[3,4-*b*]indole-3-carboxylate 9

Compound 9 was prepared from compound 8 according to the same procedure as above for perlolyrine 3. Compound 9 was obtained in 85% yield as white solid, mp 205–206 °C. ^1^H NMR (DMSO-*d*_6_, 400 MHz) *δ* 11.63 (s, 1H, N*H* in indole), 8.87 (s, 1H), 8.44 (d, *J* = 7.9 Hz, 1H), 7.82 (d, *J* = 8.2 Hz, 1H), 7.66 (dd, *J*_1_ = 7.9 Hz, *J*_2_ = 8.0 Hz, 1H), 7.35 (dd, *J*_1_ = 8.0 Hz, *J*_2_ = 8.2 Hz, 1H), 7.29 (d, *J* = 3.4 Hz, 1H), 6.63 (d, *J* = 3.4 Hz, 1H), 5.50 (t, *J* = 6.1 Hz, 1H, O*H*), 4.68 (d, *J* = 6.1 Hz, 2H), 3.94 (s, 3H). ^13^C NMR (DMSO-*d*_6_, 100 MHz) *δ* 165.92, 157.42, 151.21, 141.45, 136.65, 132.99, 132.14, 129.65, 129.01, 122.11, 121.08, 120.71, 116.43, 112.94, 110.99, 109.36, 56.07, 52.23. HRMS (ESI) *m*/*z* calcd for C_18_H_15_N_2_O_4_ [M + H]^+^: 323.1032, found: 323.1033. IR (KBr, film) 3406, 3297, 2922, 1727, 1565, 1435, 1351, 1251, 1118, 1005, 746 cm^−1^.

#### 1-(5-(Hydroxymethyl)furan-2-yl)-9*H*-pyrido[3,4-*b*]indole-3-carboxylic acid (flazin 4)

Compound 9 (0.323 g, 1.002 mmol) was dissolved in methanol (5 mL), and then an aqueous solution of NaOH (3 mol L^−1^, 1 mL) was added. The mixture was then heated and stirred at 50 °C for 5 h. After the reaction was complete (checked by TLC, eluent: EtOAc/CH_2_Cl_2_ = 1 : 1), ethyl acetate (25 mL) and water (20 mL) were added. The mixture was vigorously stirred, and pH value of the aqueous solution was adjusted to 4–5 by adding citric acid. Two phases were separated, and the aqueous solution was extracted twice with ethyl acetate (2 × 25 mL). Organic extracts were combined and washed twice with brine (2 × 10 mL). After having been dried over anhydrous MgSO_4_, the organic solution was concentrated under vacuum to give a crude solid product, which was purified by flash chromatography (eluent: MeOH/CH_2_Cl_2_ = 1 : 5) to afford pure flazin 4 (0.284 g, 0.921 mmol) in 92% yield as pale yellow solid, mp 239–240 °C. ^1^H NMR (DMSO-*d*_6_, 400 MHz) *δ* 11.69 (s, 1H, N*H* in indole), 8.84 (s, 1H), 8.42 (d, *J* = 7.8 Hz, 1H), 7.87 (d, *J* = 8.2 Hz, 1H), 7.65 (dd, *J*_1_ = 7.8 Hz, *J*_2_ = 8.0 Hz, 1H), 7.42 (d, *J* = 3.2 Hz, 1H), 7.34 (dd, *J*_1_ = 8.0 Hz, *J*_2_ = 8.2 Hz, 1H), 6.62 (d, *J* = 3.2 Hz, 1H), 4.69 (s, 2H). ^13^C NMR (DMSO-*d*_6_, 100 MHz) *δ* 166.64, 157.45, 151.40, 141.53, 137.13, 132.63, 132.06, 129.98, 129.05, 122.17, 121.11, 120.69, 115.85, 112.94, 111.22, 109.37, 56.11. HRMS (ESI) *m*/*z* calcd for C_17_H_12_N_2_O_4_Na [M + Na]^+^: 331.0695, found: 331.0697. IR (KBr, film) 3242, 2922, 1735, 1624, 1612, 1576, 1360, 1321, 1018, 742 cm^−1^.

## Conflicts of interest

There are no conflicts to declare.

## Supplementary Material

RA-008-C7RA13434G-s001
