# Supplementary material for: Cu-catalyzed mild and efficient oxidation of THβCs using air: application in practical total syntheses of perlolyrine and flazin
Source: RSC Adv. 2018 Feb 12;8(13):6834–9. doi: 10.1039/c7ra13434g (PMC9078326; doi:10.1039/c7ra13434g)

Electronic Supplementary Material (ESI) for RSC Advances.
This journal is © The Royal Society of Chemistry 2018

Supporting Information

Cu-Catalyzed Mild and Efficient Oxidation of THβCs Using Air: Application in Practical Total Syntheses of Perlolyrine and Flazin

Bo Zheng, Tien Ha Trieu, Tian-Zhuo Meng, Xia Lu, Jing Dong, Qiang Zhang, and Xiao-Xin Shi*

Shanghai Key Laboratory of Chemical Biology, School of Pharmacy, East China University of Science and Technology, 130 Mei-Long Road, Shanghai 200237, P. R. China.

E-mail: xxshi@ecust.edu.cn

Table of Contents

1. Characterization Data of β-Carbolines **2a-2x** (S2)
2. ^{1}H and ^{13}C NMR Spectra of All Compounds (S11)

1. Characterization Data of β-Carbolines 2a-2x:

1-Phenyl-9*H*-pyrido[3,4-b]indole 2a:

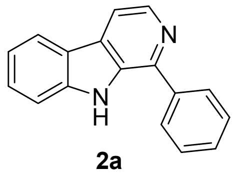

2a

Pale yellow solid, mp 240-241 °C. ^{1}H NMR (DMSO-d_6, 400 MHz) δ 11.54 (s, 1H, N*H* in indole), 8.48 (d, J = 5.2 Hz, 1H), 8.27 (d, J = 7.9 Hz, 1H), 8.13 (d, J = 5.2 Hz, 1H), 8.05 (d, J = 7.8 Hz, 2H), 7.47-7.72 (m, 5H), 7.28 (t, J = 7.6 Hz, 1H). ^{13}C NMR (DMSO-d_6, 100 MHz) δ 142.51, 141.44, 138.75, 138.69, 133,34, 129.49, 129.03 (2C), 128.80, 128.71 (2C), 128.46, 121.88, 121.16, 119.80, 114.17, 112.75. HRMS (ESI) *m/z* calcd for $C_{17}H_{13}N_2$ $[M+H]^+$: 245.1079, found: 245.1075. IR (KBr film) 3061, 2954, 2877, 1623, 1560, 1496, 1415, 1322, 1234, 737, 698 cm^{-1}.

1-(4-Nitrophenyl)-9*H*-pyrido[3,4-*b*]indole 2b:

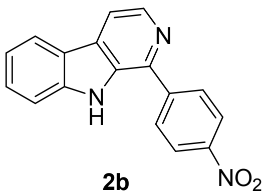

2b

Pale yellow solid, mp. 242-243 °C. ^{1}H NMR (DMSO-d_6, 400 MHz) δ 11.74 (s, 1H, N*H* in indole), 8.53 (d, J = 5.1 Hz, 1H), 8.44 (d, J = 8.5 Hz, 2H), 8.28-8.35 (m, 3H), 8.23 (d, J = 5.1 Hz, 1H), 7.66 (d, J = 8.0 Hz, 1H), 7.59 (dd, J_1 = 8.0 Hz, J_2 = 7.9 Hz, 1H), 7.30 (dd, J_1 = 7.8 Hz, J_2 = 7.9 Hz, 1H). ^{13}C NMR (DMSO-d_6, 100 MHz) δ 147.02, 144.54, 141.20, 139.37, 138.63, 133.35, 129.86, 129.50 (2C), 128.53, 123.74 (2C), 121.70, 120.65, 119.77, 115.09, 112.35. HRMS (ESI) *m/z* calcd for $C_{17}H_{12}N_3O_2$ $[M+H]^+$: 290.0930, found: 290.0942. IR (KBr film) 3368, 3060, 2922, 2847, 1624, 1599, 1510, 1455, 1425, 1348, 1226, 1103, 855, 831, 757, 740, 531 cm^{-1}.

1-(2-Nitrophenyl)-9*H*-pyrido[3,4-*b*]indole 2c:

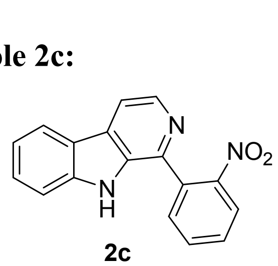

2c

Pale yellow solid, mp. 241-242 °C. ^{1}H NMR ($CDCl_3$, 400 MHz) δ 8.47 (s, 1H, N*H* in indole), 8.45 (d, J = 5.2 Hz, 1H), 8.13 (d, J = 8.0 Hz, 1H), 8.02 (d, J = 8.1 Hz, 1H), 7.95 (d, J = 5.2 Hz, 1H), 7.72 (d, J = 8.0 Hz, 1H), 7.68 (dd, J_1 = 8.1 Hz, J_2 = 8.0 Hz, 1H), 7.57 (dd, J_1 = 8.1 Hz, J_2 = 8.0 Hz, 1H), 7.52 (dd, J_1 = 8.1 Hz, J_2 = 8.0 Hz, 1H), 7.38 (d, J = 8.1 Hz, 1H), 7.30 (dd, J_1 = 8.1 Hz, J_2 = 8.0 Hz, 1H). ^{13}C NMR (DMSO-

d_6, 100 MHz) δ 149.16, 141.04, 139.34, 138.08, 133.51, 133.17, 132.59, 131.58, 129.76, 128.97, 128.43, 124.70, 121.81, 120.79, 119.65, 114.59, 112.21. HRMS (ESI) *m/z* calcd for $C_{17}H_{12}N_3O_2$ $[M+H]^+$: 290.0930, found: 290.0938. IR (KBr film) 3359, 3068, 2923, 2855, 1626, 1566, 1524, 1420, 1320, 1235, 738 cm^{-1}.

1-(4-Methoxyphenyl)-9*H*-pyrido[3,4-*b*]indole 2d:

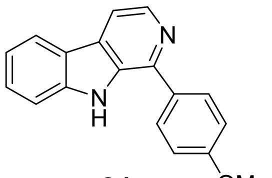

2d

White solid, mp 158-159°C. ^1H NMR (DMSO-d_6, 400 MHz) δ 11.48 (s, 1H, N*H* in indole), 8.43 (d, J = 5.2 Hz, 1H), 8.23 (d, J = 7.9 Hz, 1H), 8.05 (d, J = 5.2 Hz, 1H), 8.02 (d, J = 8.2 Hz, 2H), 7.67 (d, J = 8.1 Hz, 1H), 7.55 (dd, J_1 = 8.1 Hz, J_2 = 8.0 Hz,, 1H), 7.25 (dd, J_1 = 7.9 Hz, J_2 = 8.0 Hz, 1H), 7.17 (d, J = 8.2 Hz, 2H), 3.87 (s, 3H). ^{13}C NMR (DMSO-d_6, 100 MHz) δ 160.06 142.60, 141.53, 138.72, 133.24, 131.39, 130.14 (2C), 129.46, 128.46, 121.97, 121.37, 119.88, 114.57 (2C), 113.74, 112.88, 55.73. HRMS (ESI) *m/z* calcd for $C_{18}H_{15}N_2O$ $[M+H]^+$: 275.1184, found: 275.1180. IR (KBr film) 3141, 3064, 2980, 1626, 1610, 1514, 1470, 1430, 1322, 1254, 1238, 1175, 1038, 742 cm^{-1}.

1-(3-(Trifluoromethyl)-phenyl)-9*H*-pyrido[3,4-*b*]indole 2e:

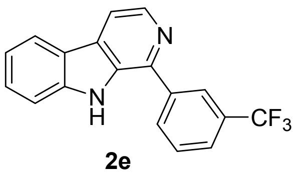

2e

White solid, mp. 140-141°C. ^1H NMR ($CDCl_3$, 400 MHz) δ 8.76 (s, 1H, N*H* in indole), 8.58 (d, J = 5.2 Hz, 1H), 8.21 (s, 1H), 8.17 (d, J = 7.9 Hz, 1H), 8.10 (d, J = 7.7 Hz, 1H), 7.98 (d, J = 5.2 Hz, 1H), 7.70 (d, J = 7.8 Hz, 1H), 7.64 (dd, J_1 = 7.9 Hz, J_2 = 7.8 Hz, 1H), 7.57 (dd, J_1 = 7.8 Hz, J_2 = 7.7 Hz, 1H), 7.51 (d, J = 8.0 Hz, 1H), 7.33 (dd, J_1 = 7.8 Hz, J_2 = 8.0 Hz, 1H). ^{13}C NMR ($CDCl_3$, 100 MHz) δ 141.18, 140.82, 139.18, 139.10, 133.64, 131.33, 131.28 (q, $^2J_{CF}$ = 32.5 Hz), 130.45, 129.37, 128.76, 125.30 (q, $^3J_{CF}$ = 4.1 Hz), 124.98 (q, $^3J_{CF}$ = 4.1 Hz), 123.95 (q, $^1J_{CF}$ = 272.6 Hz), 121.79, 121.68, 120.40, 114.49, 111.77. HRMS (ESI) *m/z* calcd for $C_{18}H_{12}F_3N_2$ $[M+H]^+$: 313.0953, found: 313.0950. IR (KBr film) 3127, 3062, 2955, 2876, 1626, 1566, 1499, 1456, 1338, 1276, 1234, 1167, 1125, 1071, 807, 744, 699 cm^{-1}.

1-(3,4-Dimethoxyphenyl)-9*H*-pyrido[3,4-*b*]indole 2f:

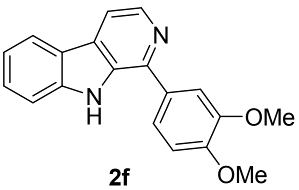

2f

White solid, mp 106-107°C. ^{1}H NMR ($CDCl_3$, 400 MHz) δ 9.07 (s, 1H, N*H* in indole), 8.53 (d, J = 5.2 Hz, 1H), 8.15 (d, J = 8.0 Hz, 1H), 7.91 (d, J = 5.2 Hz, 1H), 7.43-7.57 (m, 5H), 7.30 (dd, J_1 = 7.9 Hz, J_2 = 8.0 Hz, 1H), 6.89 (d, J = 8.0 Hz, 1H), 3.88 (s, 3H), 3.85 (s, 3H). ^{13}C NMR (DMSO-d_6, 100 MHz) δ 149.76, 149.38, 142.77, 141.51, 138.65, 133.31, 131.58, 129.44, 128.48, 122.00, 121.37, 121.24, 119.89, 113.81, 112.90, 112.29, 112.23, 56.14, 55.89. HRMS (ESI) *m/z* calcd for $C_{19}H_{17}N_2O_2$ $[M+H]^+$: 305.1290, found: 305.1282. IR (KBr film) 3363, 2935, 1626, 1516, 1457, 1409, 1319, 1260, 1144, 1025, 747 cm^{-1}.

1-(Naphthalen-1-yl)-9*H*-pyrido[3,4-*b*]indole 2g:

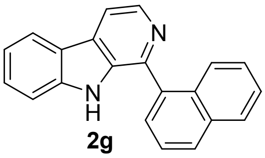

2g

White solid, mp. 114-115 °C. ^{1}H NMR (DMSO-d_6, 400 MHz) δ 11.16 (s, 1H, N*H* in indole), 8.52 (d, J = 5.2 Hz, 1H), 8.30 (d, J = 7.9 Hz, 1H), 8.21 (d, J = 5.2 Hz, 1H), 8.12 (d, J = 7.8 Hz, 1H), 8.07 (d, J = 8.0 Hz, 1H), 7.68-7.77 (m, 3H), 7.48-7.58 (m, 3H), 7.44 (dd, J_1 = 7.8 Hz, J_2 = 7.9 Hz, 1H), 7.26 (dd, J_1 = 7.9 Hz, J_2 = 8.0 Hz, 1H). ^{13}C NMR (DMSO-d_6, 100 MHz) δ 143.22, 141.40, 138.47, 136.09, 135.15, 134.11, 131.69, 129.15, 128.98, 128.77, 128.60, 127.98, 126.82, 126.52, 126.13, 126.04, 122.19, 121.34, 119.86, 114.48, 112.72. HRMS (ESI) *m/z* calcd for $C_{21}H_{15}N_2$ $[M+H]^+$: 295.1235, found: 295.1242. IR (KBr film) 3409, 3130, 3054, 2924, 2853, 1624, 1563, 1454, 1419, 1319, 1235, 777, 744 cm^{-1}.

1-(Thiophen-2-yl)-9*H*-pyrido[3,4-*b*]indole 2h:

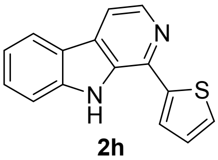

2h

Pale yellow solid, mp. 165-166 °C. ^{1}H NMR ($CDCl_3$, 400 MHz) δ 8.90 (s, 1H, N*H* in indole), 8.48 (d, J = 5.2 Hz, 1H), 8.11 (d, J = 7.9 Hz, 1H), 7.86 (d, J = 5.2 Hz, 1H), 7.74 (d, J = 3.6 Hz, 1H), 7.50-7.58 (m, 2H), 7.46 (d, J = 5.0 Hz, 1H), 7.30 (dd, J_1 = 7.8 Hz, J_2 = 7.9 Hz, 1H), 7.18 (dd, J_1 = 5.0 Hz, J_2 = 3.6 Hz, 1H). ^{13}C NMR ($CDCl_3$, 100 MHz) δ 142.83, 140.64, 139.18, 137.10, 132.27, 130.40, 128.64, 128.04, 127.20, 125.17, 121.80, 121.71, 120.49, 113.81, 111.83. HRMS (ESI) *m/z* calcd for $C_{15}H_{11}N_2S$ $[M+H]^+$ 251.0643, found: 251.0642. IR (KBr film) 3204, 3057, 2924, 2853, 1623, 1562, 1495, 1453, 1421, 1319, 1232, 743, 702 cm^{-1}.

1-(Pyridin-2-yl)-9*H*-pyrido[3,4-*b*]indole 2i:

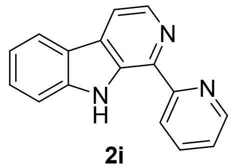

2i

Pale yellow solid, mp 179-180 °C. ^{1}H NMR ($CDCl_3$, 400 MHz) δ 11.33 (s, 1H, N*H* in indole), 8.78 (d, J = 5.0 Hz, 1H), 8.75 (d, J = 8.0 Hz, 1H), 8.55 (d, J = 5.1 Hz, 1H), 8.16 (d, J = 7.9 Hz, 1H), 8.00 (d, J = 5.1 Hz, 1H), 7.89 (dd, J_1 = 7.9 Hz, J_2 = 8.0 Hz, 1H), 7.55-7.63 (m, 2H), 7.27-7.36 (m, 2H). ^{13}C NMR ($CDCl_3$, 100 MHz) δ 157.98, 148.26, 140.63, 138.14, 138.09, 136.85, 134.77, 130.48, 128.44, 122.92, 121.69, 121.30, 121.15, 119.80, 115.47, 111.88. HRMS (ESI) *m/z* calcd for $C_{16}H_{12}N_3$ $[M+H]^+$: 246.1031, found: 246.1032. IR (KBr film) 3397, 3296, 3060, 2922, 1627, 1590, 1566, 1438, 1448, 1417, 1318, 1236, 1149, 799, 743 cm^{-1}.

1-Methyl-9*H*-pyrido[3,4-*b*]indole 2j:

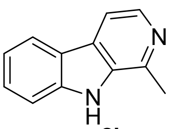

2j

Pale yellow solid, mp 221-222 °C. ^{1}H NMR ($CDCl_3$, 400 MHz) δ 9.66 (s, 1H, N*H* in indole), 8.38 (d, J = 5.4 Hz, 1H), 8.12 (d, J = 7.9 Hz, 1H), 7.84 (d, J = 5.4 Hz, 1H), 7.46-7.54 (m, 2H), 7.25-7.30 (m, 1H), 2.83 (s, 3H). ^{13}C NMR ($CDCl_3$, 100 MHz) δ 141.82, 140.24, 138.51, 134.68, 128.34, 128.23, 122.02, 121.84, 120.07, 112.96, 111.62, 20.31. HRMS (ESI) *m/z* calcd for $C_{12}H_{11}N_2$ $[M+H]^+$: 183.0922, found: 183.0926. IR (KBr film) 3128, 3063, 2954, 2854, 1625, 1565, 1507, 1255, 1237, 741 cm^{-1}.

1-Ethyl-9*H*-pyrido[3,4-*b*]indole 2k:

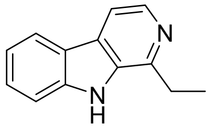

2k

White solid, mp 195-196 °C. ^{1}H NMR (DMSO-d_6, 400 MHz) δ 11.62 (s, 1H, N*H* in indole), 8.27 (d, J = 5.3 Hz, 1H), 8.16 (d, J = 7.9 Hz, 1H), 7.90 (d, J = 5.3 Hz, 1H), 7.62 (d, J = 8.2 Hz, 1H), 7.53 (dd, J_1 = 8.2 Hz, J_2 = 8.0 Hz, 1H), 7.21 (dd, J_1 = 7.9 Hz, J_2 = 8.0 Hz, 1H), 3.17 (q, J = 7.5 Hz, 2H), 1.39 (t, J = 7.5 Hz, 3H). ^{13}C NMR (DMSO-d_6, 100 MHz) δ 147.36, 140.86, 138.00, 134.26, 128.20, 127.59, 122.03, 121.61, 119.57, 113.05, 112.38, 27.14, 13.13. HRMS (ESI) *m/z* calcd for $C_{13}H_{13}N_2$ $[M+H]^+$: 197.1079, found: 197.1082. IR (KBr film) 3171, 2926, 2855, 1626, 1458, 1430, 1401, 1324, 1240, 1155, 745 cm^{-1}.

3-Methyl-1-phenyl-9*H*-pyrido[3,4-*b*]indole 2l:

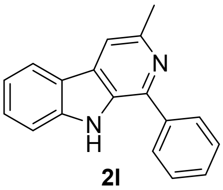

2l

Pale yellow solid, mp. 128-129 °C. ^{1}H NMR ($CDCl_3$, 400 MHz) δ 8.51 (s, 1H, N*H* in indole), 8.08 (d, *J* = 7.9 Hz, 1H), 7.88 (d, *J* = 7.2 Hz, 2H), 7.74 (s, 1H), 7.45-7.55 (m, 3H), 7.36-7.42 (m, 2H), 7.23 (t, *J* = 7.2 Hz, 1H), 2.76 (s, 3H). ^{13}C NMR ($CDCl_3$, 100 MHz) δ 147.85, 141.98, 140.83, 138.60, 131.91, 130.87, 129.15 (2C), 128.66, 128.38, 128.23 (2C), 121.76, 119.96, 112.71, 111.49, 24.39. HRMS (ESI) *m/z* calcd for $C_{18}H_{15}N_2$ $[M+H]^+$: 259.1235, found: 259.1242. IR (KBr film) 3132, 3064, 2916, 1625, 1561, 1496, 1452, 1440, 1320, 1241, 742, 703 cm^{-1}.

1-(4-Methoxyphenyl)-3-methyl-9*H*-pyrido[3,4-*b*]indole 2m:

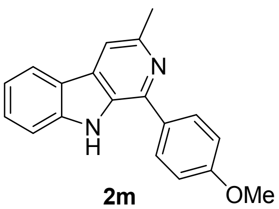

2m

Pale yellow solid, mp. 131-132 °C. ^{1}H NMR ($CDCl_3$, 400 MHz) δ 8.82 (s, 1H, N*H* in indole), 8.05 (d, *J* = 7.9 Hz, 1H), 7.76 (d, *J* = 8.2 Hz, 2H), 7.68 (s, 1H), 7.46 (dd, J_1 = 7.9 Hz, J_2 = 8.0 Hz, 1H), 7.38 (d, *J* = 8.1 Hz, 1H), 7.22 (dd, J_1 = 8.0 Hz, J_2 = 8.1 Hz, 1H), 6.90 (d, *J* = 8.2 Hz, 2H), 3.72 (s, 3H), 2.73 (s, 3H). ^{13}C NMR ($CDCl_3$, 100 MHz) δ 159.85, 147.56, 141.97, 140.96, 131.87, 131.15, 130.69, 129.46 (2C), 128.19, 121.80, 121.67, 119.78, 114.34 (2C), 112.21, 111.55, 55.29, 24.31. HRMS (EI) *m/z* calcd for $C_{19}H_{17}N_2O$ $[M+H]^+$: 289.1341, found: 289.1340. IR (KBr film) 3126, 3055, 2918, 1624, 1611, 1563, 1513, 1251, 1237, 744, 586 cm^{-1}.

3-Methyl-1-(thiophen-2-yl)-9*H*-pyrido[3,4-*b*]indole 2n:

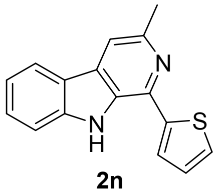

2n

Pale yellow solid, mp. 161-162 °C. ^{1}H NMR ($CDCl_3$, 400 MHz) δ 8.54 (s, 1H, N*H* in indole), 8.08 (d, *J* = 7.9 Hz, 1H), 7.72 (s, 1H), 7.71 (d, *J* = 4.8 Hz, 1H), 7.45-7.55 (m, 3H), 7.29 (d, *J* = 7.8 Hz, 1H), 7.20 (dd, J_1 = 4.8 Hz, J_2 = 3.7 Hz, 1H), 2.75 (s, 3H). ^{13}C NMR ($CDCl_3$, 100 MHz) δ 147.89, 142.84, 140.94, 135.89, 131.44, 130.76, 128.52, 127.98, 126.95, 125.11, 121.79, 121.71, 120.27, 112.82, 111.69, 24.29.

HRMS (ESI) *m/z* calcd for $C_{16}H_{13}N_2S$ $[M+H]^+$: 265.0799, found: 265.0796. IR (KBr film) 3344, 3062, 2911, 1624, 1558, 1493, 1438, 1323, 1236, 740, 711 cm^{-1}.

Methyl 1-phenyl-9*H*-pyrido[3,4-*b*]indole-3-carboxylate 2o:

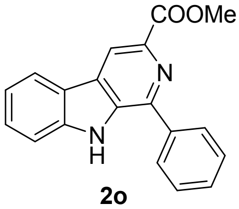

2o

White solid, mp 256-257 °C. ^1H NMR (DMSO-d_6, 400 MHz) δ 11.92 (s, 1H, N*H* in indole), 8.92 (s, 1H), 8.42 (d, J = 8.0 Hz, 1H), 8.05 (d, J = 7.9 Hz, 2H), 7.70 (d, J = 8.0 Hz, 1H), 7.52-7.66 (m, 4H), 7.33 (dd, J_1 = 8.0 Hz, J_2 = 7.9 Hz, 1H), 3.94 (s, 3H). ^{13}C NMR (DMSO-d_6, 100 MHz) δ 166.57, 142.56, 142.21, 138.08, 136.98, 135.29, 129.65, 129.38, 129.20 (2C), 129.11 (2C), 129.03, 122.39, 121.64, 120.75, 117.13, 113.35, 52.48. HRMS (ESI) *m/z* calcd for $C_{19}H_{15}N_2O_2$ $[M+H]^+$: 303.1134, found: 303.1136. IR (KBr film) 3319, 2952, 1723, 1626, 1352, 1253, 1217, 1103, 1049, 740 cm^{-1}.

Methyl 1-(4-methoxyphenyl)-9*H*-pyrido[3,4-*b*]indole-3-carboxylate 2p:

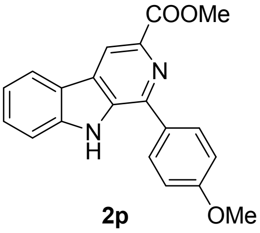

2p

White solid, mp 229-230 °C. ^1H NMR (DMSO-d_6, 400 MHz) δ 11.89 (s, 1H, N*H* in indole), 8.88 (s, 1H), 8.41 (d, J = 7.6 Hz, 1H), 7.99 (d, J = 8.6 Hz, 2H), 7.70 (d, J = 7.9 Hz, 1H), 7.60 (dd, J_1 = 7.9 Hz, J_2 = 7.8 Hz, 1H), 7.32 (dd, J_1 = 7.6 Hz, J_2 = 7.8 Hz, 1H), 7.20 (d, J = 8.6 Hz, 2H), 3.93 (s, 3H), 3.88 (s, 3H). ^{13}C NMR (DMSO-d_6, 100 MHz) δ 166.59, 160.41, 142.46, 141.90, 137.07, 134.84, 130.46, 130.43 (2C), 129.44, 128.99, 122.38, 121.66, 120.79, 116.64, 114.63 (2C), 113.24, 55.80, 52.46. HRMS (ESI) *m/z* calcd for $C_{20}H_{17}N_2O_3$ $[M+H]^+$: 333.1239, found: 333.1241. IR (KBr film) 3254, 2951, 2837, 1714, 1611, 1514, 1434, 1353, 1251, 1177, 1116, 1032, 746 cm^{-1}.

Ethyl 1-(3,4-dimethoxyphenyl)-9*H*-pyrido[3,4-*b*]indole-3-carboxylate 2q:

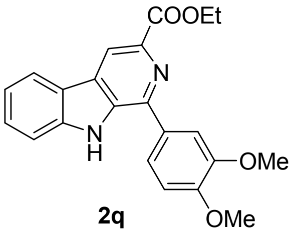

2q

White solid, mp 217-218 °C. ^{1}H NMR (DMSO-d_6, 400 MHz) δ 11.89 (s, 1H, N*H* in indole), 8.87 (s, 1H), 8.41 (d, J = 7.9 Hz, 1H), 7.70 (d, J = 8.1 Hz, 1H), 7.54-7.64 (m, 3H), 7.32 (dd, J_1 = 7.9 Hz, J_2 = 8.0 Hz, 1H), 7.21 (d, J = 7.9 Hz, 1H), 4.41 (q, J = 7.1 Hz, 2H), 3.91 (s, 3H), 3.89 (s, 3H), 1.39 (t, J = 7.1 Hz, 3H). ^{13}C NMR (DMSO-d_6, 100 MHz) δ 166.04, 150.10, 149.30, 142.73, 141.89, 137.28, 134.94, 130.69, 129.35, 128.95, 122.37, 121.70, 121.71, 120.76, 116.59, 113.24, 112.55, 112.31, 61.06, 56.16, 55.95, 14.83. HRMS (ESI) *m/z* calcd for $C_{22}H_{21}N_2O_4$ [M+H]$^+$: 377.1501, found: 377.1502. IR (KBr film) 3423, 2937, 1709, 1626, 1516, 1371, 1348, 1257, 1143, 1025, 746 cm^{-1}.

Ethyl 1-(3-(trifluoromethyl)phenyl)-9*H*-pyrido[3,4-*b*]indole-3-carboxylate 2r:

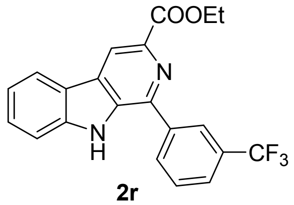

2r

White solid, mp 209-210 °C. ^{1}H NMR (DMSO-d_6, 400 MHz) δ 12.06 (s, 1H, N*H* in indole), 8.96 (s, 1H), 8.45 (d, J = 7.9 Hz, 1H), 8.32 (d, J = 7.6 Hz, 1H), 8.27 (s, 1H), 7.86-7.94 (m, 2H), 7.70 (d, J = 8.0 Hz, 1H), 7.63 (dd, J_1 = 7.8 Hz, J_2 = 7.9 Hz, 1H), 7.35 (dd, J_1 = 7.9 Hz, J_2 = 8.0 Hz, 1H), 4.42 (q, J = 7.1 Hz, 2H), 1.39 (t, J = 7.1 Hz, 3H). ^{13}C NMR (DMSO-d_6, 100 MHz) δ 165.77, 142.03, 140.77, 138.92, 137.47, 135.10, 132.99, 130.34, 130.07 (q, $^2J_{CF}$ = 31.0 Hz), 130.05, 129.31, 126.00 (q, $^3J_{CF}$ = 4.1 Hz), 125.61 (q, $^3J_{CF}$ = 4.1 Hz), 124.68 (q, $^1J_{CF}$ = 271.0 Hz), 122.51, 121.56, 120.97, 117.55, 113.17, 61.17, 14.78. HRMS (ESI) *m/z* calcd for $C_{21}H_{16}F_3N_2O_2$ [M+H]$^+$: 385.1164, found: 385.1160. IR (KBr film) 3260, 3058, 2988, 2908, 1724, 1624, 1567, 1454, 1373, 1330, 1255, 1160, 1120, 1095, 742, 541 cm^{-1}.

Ethyl 1-(naphthalen-1-yl)-9*H*-pyrido[3,4-*b*]indole-3-carboxylate 2s:

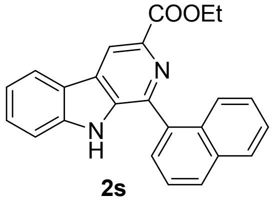

2s

White solid, mp 275-276 °C. ^{1}H NMR (DMSO-d_6, 400 MHz) δ 11.63 (s, 1H, N*H* in indole), 9.03 (s, 1H), 8.47 (d, J = 7.9 Hz, 1H), 8.16 (d, J = 7.8 Hz, 1H), 8.10 (d, J = 8.0 Hz, 1H), 7.72-7.78 (m, 2H), 7.53-7.60 (m, 4H), 7.44 (dd, J_1 = 7.8 Hz, J_2 = 7.9 Hz, 1H), 7.32 (dd, J_1 = 7.9 Hz, J_2 = 8.0 Hz, 1H), 4.39 (q, J = 7.1 Hz, 2H), 1.35 (t, J = 7.1 Hz, 3H). ^{13}C NMR (DMSO-d_6, 100 MHz) δ 166.12, 143.02, 141.89, 137.13, 136.84, 135.31, 134.02, 131.70, 129.50, 129.10, 128.95, 128.83, 128.16, 127.05, 126.62, 126.13, 125.80, 122.59, 121.67, 120.76, 117.44, 113.12, 61.12, 14.85. HRMS (ESI) *m/z* calcd for $C_{24}H_{19}N_2O_2$ [M+H]$^+$: 367.1447, found: 367.1445. IR (KBr film) 3408, 3234, 2982, 1712, 1626, 1502, 1345, 1226, 752 cm^{-1}.

Ethyl 1-(thiophen-2-yl)-9*H*-pyrido[3,4-*b*]indole-3-carboxylate 2t:

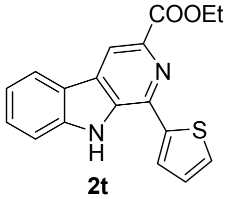

2t

White solid, mp 162-163 °C. ^{1}H NMR (DMSO-d_6, 400 MHz) δ 11.90 (s, 1H, N*H* in indole), 8.85 (s, 1H), 8.42 (d, J = 7.9 Hz, 1H), 8.15 (d, J = 3.6 Hz, 1H), 7.75-7.81 (m, 2H), 7.63 (dd, J_1 = 7.9 Hz, J_2 = 8.0 Hz, 1H), 7.32-7.38 (m, 2H), 4.41 (q, J = 7.1 Hz, 2H), 1.40 (t, J = 7.1 Hz, 3H). ^{13}C NMR (DMSO-d_6, 100 MHz) δ 165.55, 142.91, 142.01, 137.05, 136.80, 132.89, 130.20, 129.29, 129.28, 128.87, 127.09, 122.38, 121.54, 121.14, 116.81, 113.38, 61.18, 14.82. HRMS (ESI) *m/z* calcd for $C_{18}H_{15}N_2O_2S$ [M+H]$^+$: 323.0854, found: 323.0853. IR (KBr film) 3345, 3094, 2987, 2925, 1708, 1672, 1624, 1563, 1368, 1330, 1254, 1097, 1024, 740, 524 cm^{-1}.

Ethyl 1-methyl-9*H*-pyrido[3,4-*b*]indole-3-carboxylate 2u:

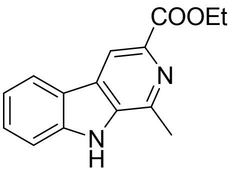

2u

White solid, mp 217-218 °C. ^{1}H NMR (DMSO-d_6, 400 MHz) δ 12.04 (s, 1H, N*H* in indole), 8.77 (s, 1H), 8.36 (d, J = 7.9 Hz, 1H), 7.67 (d, J = 8.1 Hz, 1H), 7.60 (dd, J_1 = 7.9 Hz, J_2 = 8.0 Hz, 1H), 7.31 (dd, J_1 = 8.0 Hz, J_2 = 8.1 Hz, 1H), 4.38 (q, J = 7.1 Hz, 2H), 2.83 (s, 3H), 1.38 (t, J = 7.1 Hz, 3H). ^{13}C NMR (DMSO-d_6, 100 MHz) δ 166.13, 142.59, 141.21, 136.68, 136.61, 128.75, 127.22, 122.48, 121.82, 120.54, 116.30, 112.75, 60.89, 20.83, 14.84. HRMS (ESI) *m/z* calcd for $C_{15}H_{15}N_2O_2$ [M+H]$^+$: 255.1134, found: 255.1142. IR (KBr film) 3317, 3041, 2978, 1709, 1624, 1596, 1568, 1500, 1345, 1256, 1239, 741 cm^{-1}.

Ethyl 1-ethyl-9*H*-pyrido[3,4-*b*]indole-3-carboxylate 2v:

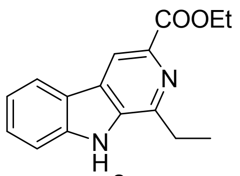

2v

White solid, mp 210-211°C. ^{1}H NMR (DMSO-d_6, 400 MHz) δ 12.02 (s, 1H, N*H* in indole), 8.76 (s, 1H), 8.35 (d, J = 7.9 Hz, 1H), 7.65 (d, J = 8.1 Hz, 1H), 7.58 (dd, J_1 = 7.9 Hz, J_2 = 8.0 Hz, 1H), 7.29 (dd, J_1 = 8.1 Hz, J_2 = 8.0 Hz, 1H), 4.37 (q, J = 7.1 Hz, 2H), 3.17 (q, J = 7.5 Hz, 2H), 1.37 (t, J = 7.1 Hz, 3H), 1.36 (t, J = 7.5 Hz, 3H). ^{13}C NMR (DMSO-d_6, 100 MHz) δ 166.14, 147.40, 141.23, 136.80, 135.85, 128.75,

127.53, 122.42, 121.83, 120.51, 116.34, 112.77, 60.88, 27.27, 14.85, 13.19. HRMS (ESI) *m/z* calcd for $C_{16}H_{17}N_2O_2$ $[M+H]^+$: 269.1290, found: 269.1295. IR (KBr film) 3328, 2994, 2929, 1704, 1565, 1498, 1366, 1346, 1258, 1235, 1046, 749 cm^{-1}.

Ethyl 6-ethoxy-1-phenyl-9*H*-pyrido[3,4-b]indole-3-carboxylate 2w:

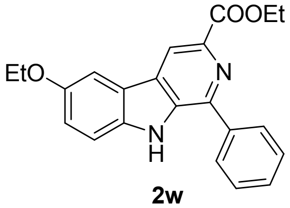

2w

Pale yellow solid, m.p.: 228-229 °C. ^1H NMR (400 MHz, DMSO-d_6) δ 11.75 (s, 1H, N*H* in indole), 8.92 (s, 1H), 8.06–7.96 (m, 3H), 7.64 (d, J = 7.4 Hz, 2H), 7.61–7.53 (m, 2H), 7.22 (d, J = 8.9 Hz, 1H), 4.41 (q, J = 7.1 Hz, 2H), 4.15 (q, J = 6.9 Hz, 2H), 1.40 (t, J = 6.9 Hz, 3H), 1.39 (t, J = 7.1 Hz, 3H). ^{13}C NMR (100 MHz, DMSO-d_6) δ 165.62, 153.35, 142.09, 137.69, 136.34, 136.21, 134.96, 129.00, 128.96, 128.81, 128.59, 121.65, 119.29, 116.94, 113.63, 104.29, 63.61, 60.61, 14.83, 14.45. IR (KBr film) 3448, 3227, 2980, 1712 (C=O), 1525, 1462, 1306, 1206, 1151, 1103, 1027, 725, 696 cm^{-1}. HRMS (ESI) *m/z* calcd for $C_{22}H_{21}N_2O_3$ $[M+H]^+$: 361.1552; found: 361.1549.

Ethyl 1-(3,4-dimethoxyphenyl)-6-methoxy-4,9-dihydro-3*H*-pyrido[3,4-b]indole-3-carboxylate 2x:

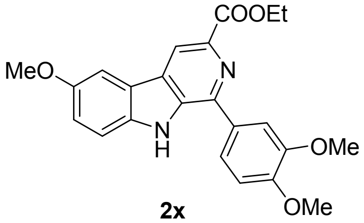

2x

Pale yellow solid, m.p.: 207-208 °C. ^1H NMR (400 MHz, DMSO-d_6) δ 11.72 (s, 1H, N*H* in indole), 8.88 (s, 1H), 8.00 (s, 1H), 7.69–7.42 (m, 3H), 7.23 (d, J = 8.9 Hz, 1H), 7.20 (d, J = 8.9 Hz, 1H), 4.40 (q, J = 7.1 Hz, 2H), 3.90 (s, 3H), 3.89 (s, 3H), 3.88 (s, 3H), 1.39 (t, J = 7.1 Hz, 3H). ^{13}C NMR (100 MHz, DMSO-d_6) δ 165.66, 154.14, 149.63, 148.84, 142.32, 136.22, 136.21, 134.90, 130.32, 128.75, 121.73, 121.20, 118.80, 116.48, 113.67, 112.02, 111.82, 103.50, 60.60, 55.71, 55.66, 55.51, 14.44. IR (KBr film) 3449, 3262, 2937, 1704 (C=O), 1514, 1495, 1463, 1303, 1266, 1240, 1219, 1104, 1033, 778, 759 cm^{-1}. HRMS (ESI) *m/z* calcd for $C_{23}H_{23}N_2O_5$ $[M+H]^+$: 407.1607; found: 407.1609.

2. ^{1}H and ^{13}C NMR Spectra of All Compounds:

^{1}H NMR (DMSO-d_6, 400 MHz) spectrum of compound **2a**:

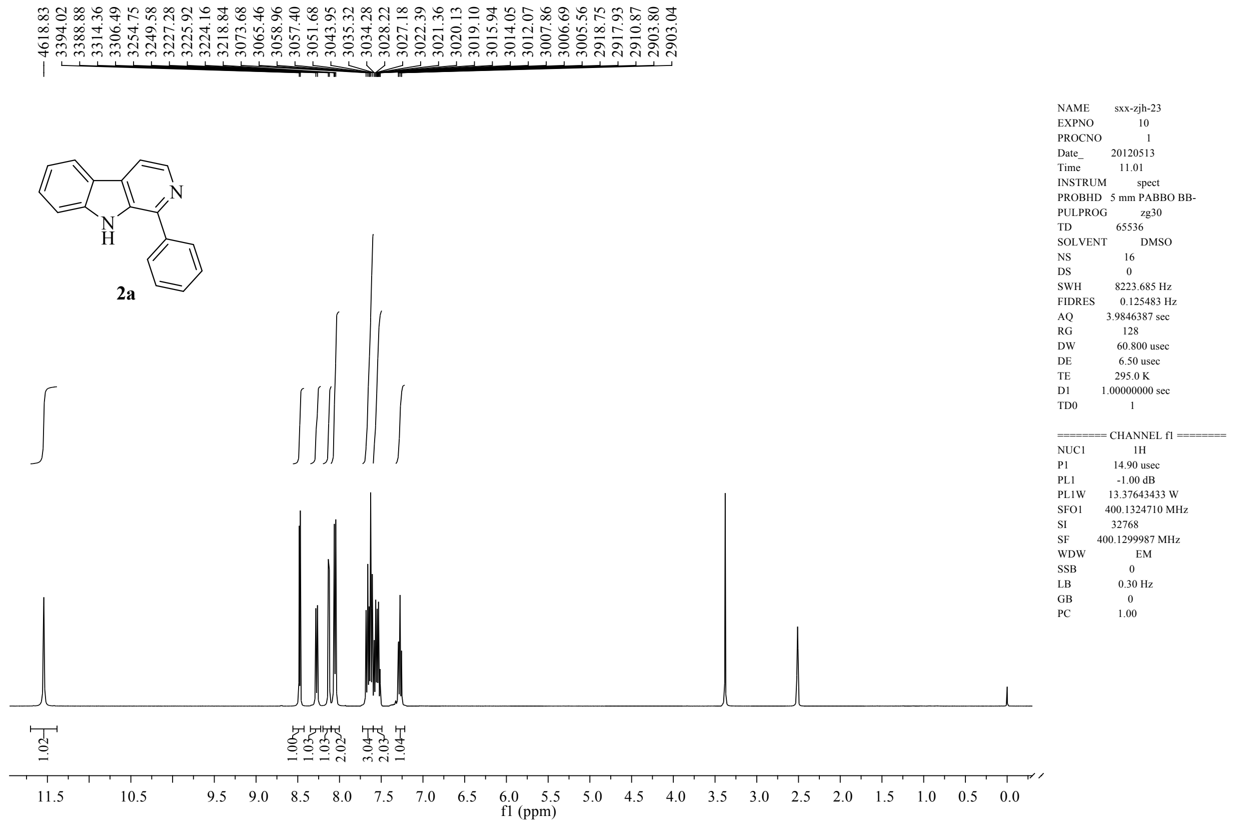

^{13}C NMR (DMSO-d_6, 100 MHz) spectrum of compound **2a**:

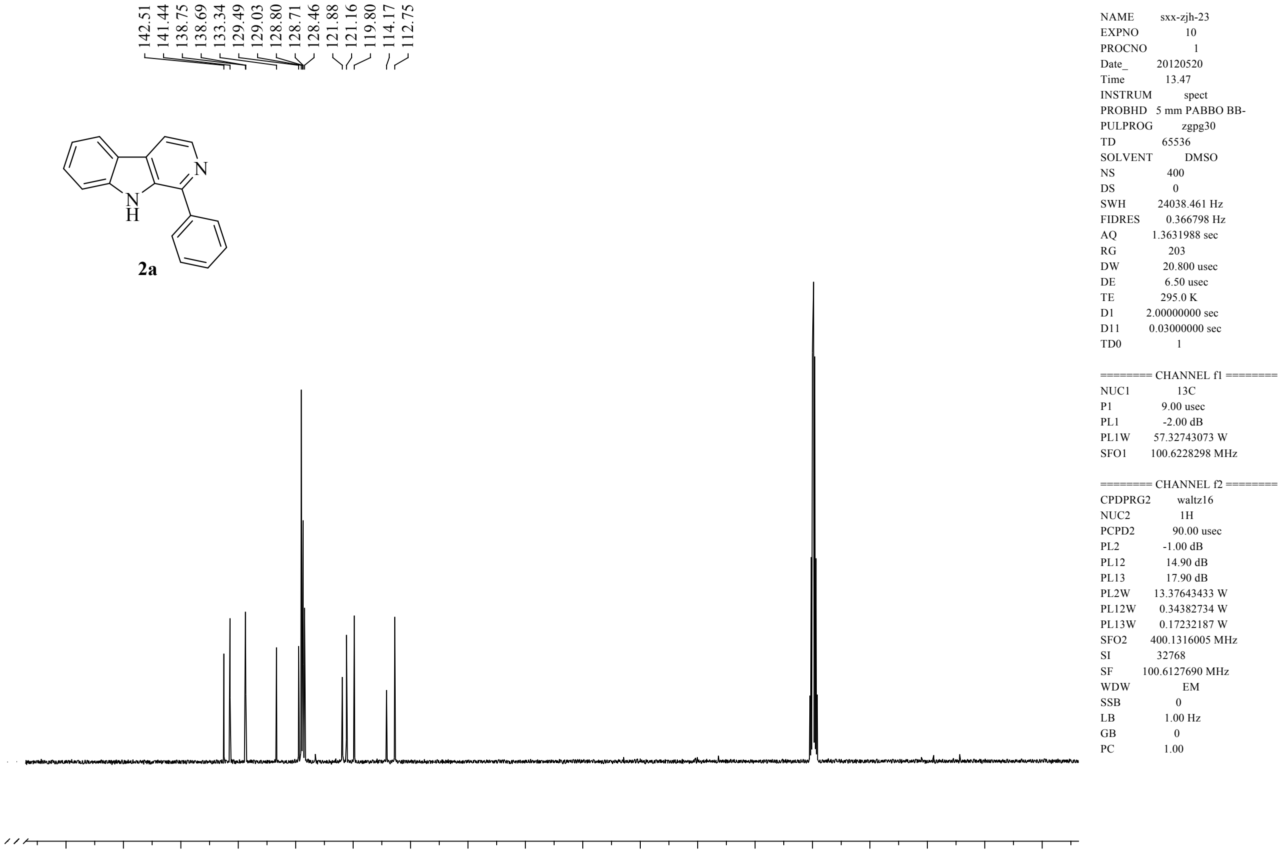

^1H NMR (DMSO-d_6, 400 MHz) spectrum of compound **2b**:

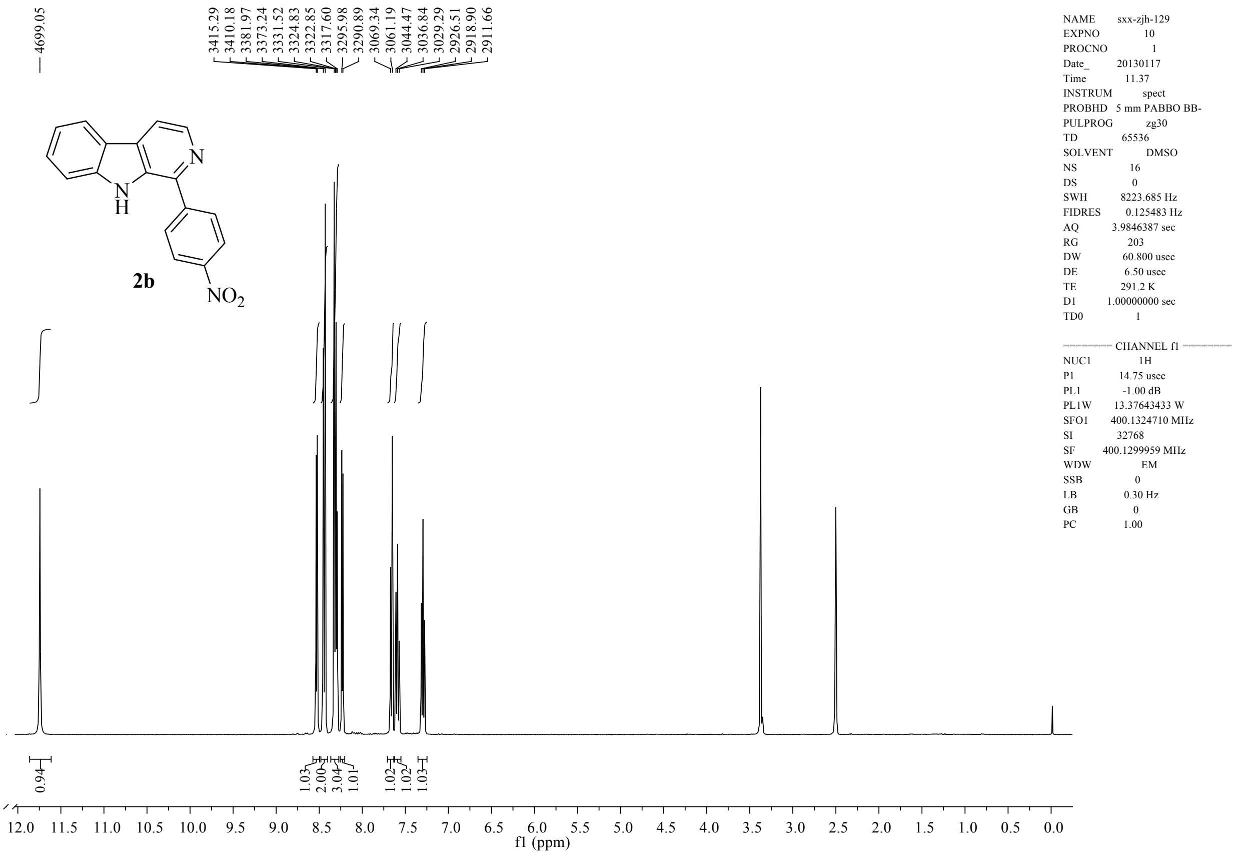

^{13}C NMR (DMSO-d_6, 100 MHz) spectrum of compound **2b**:

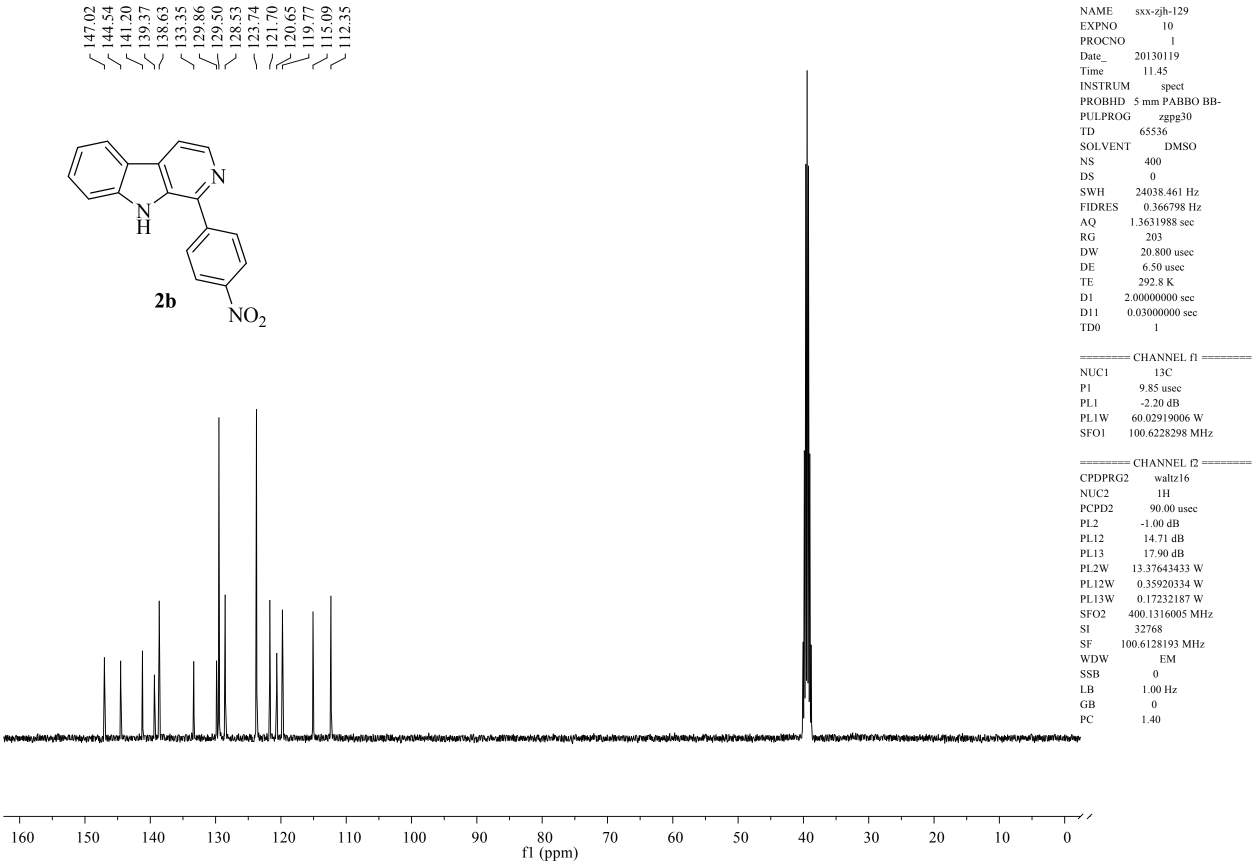

^{1}H NMR ($CDCl_3$, 400 MHz) spectrum of compound **2c**:

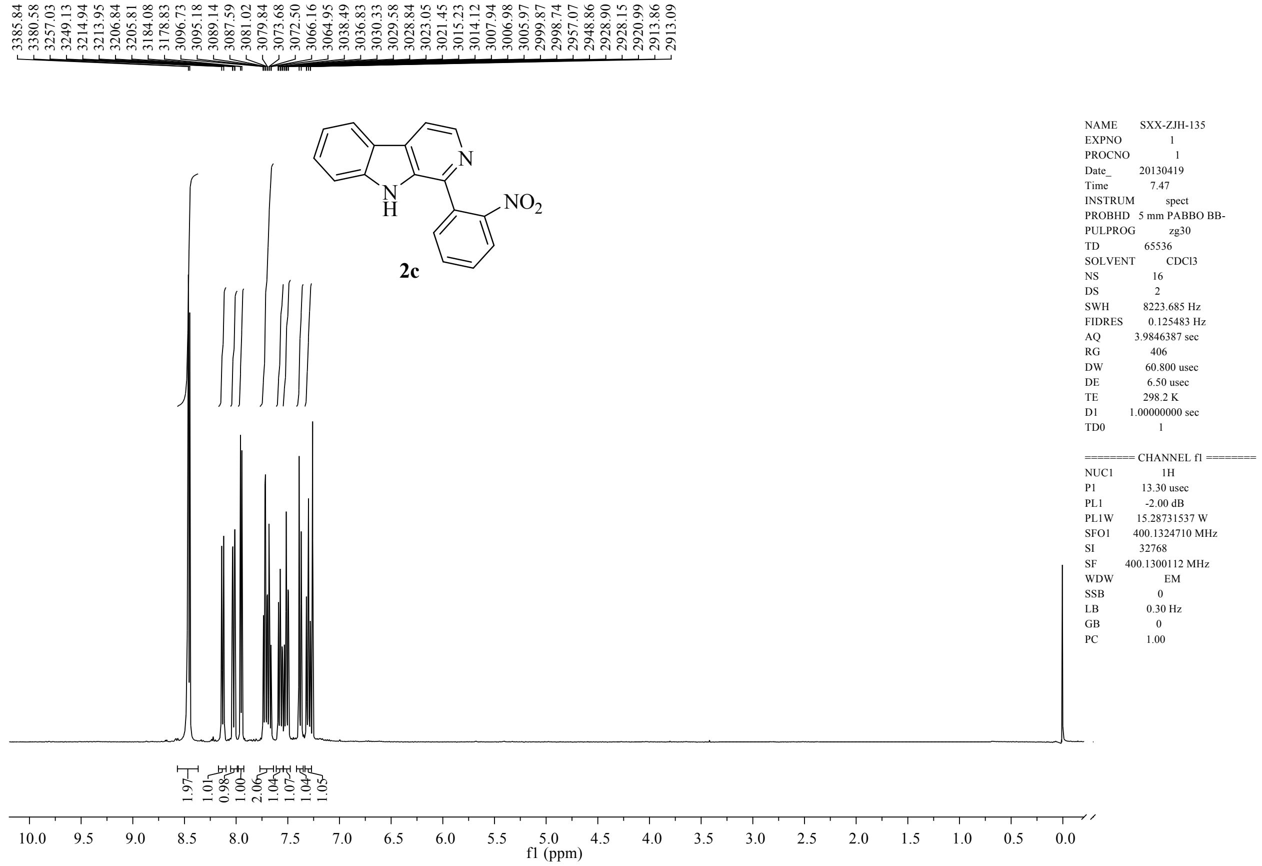

^{13}C NMR (DMSO-d_6, 100 MHz) spectrum of compound **2c**:

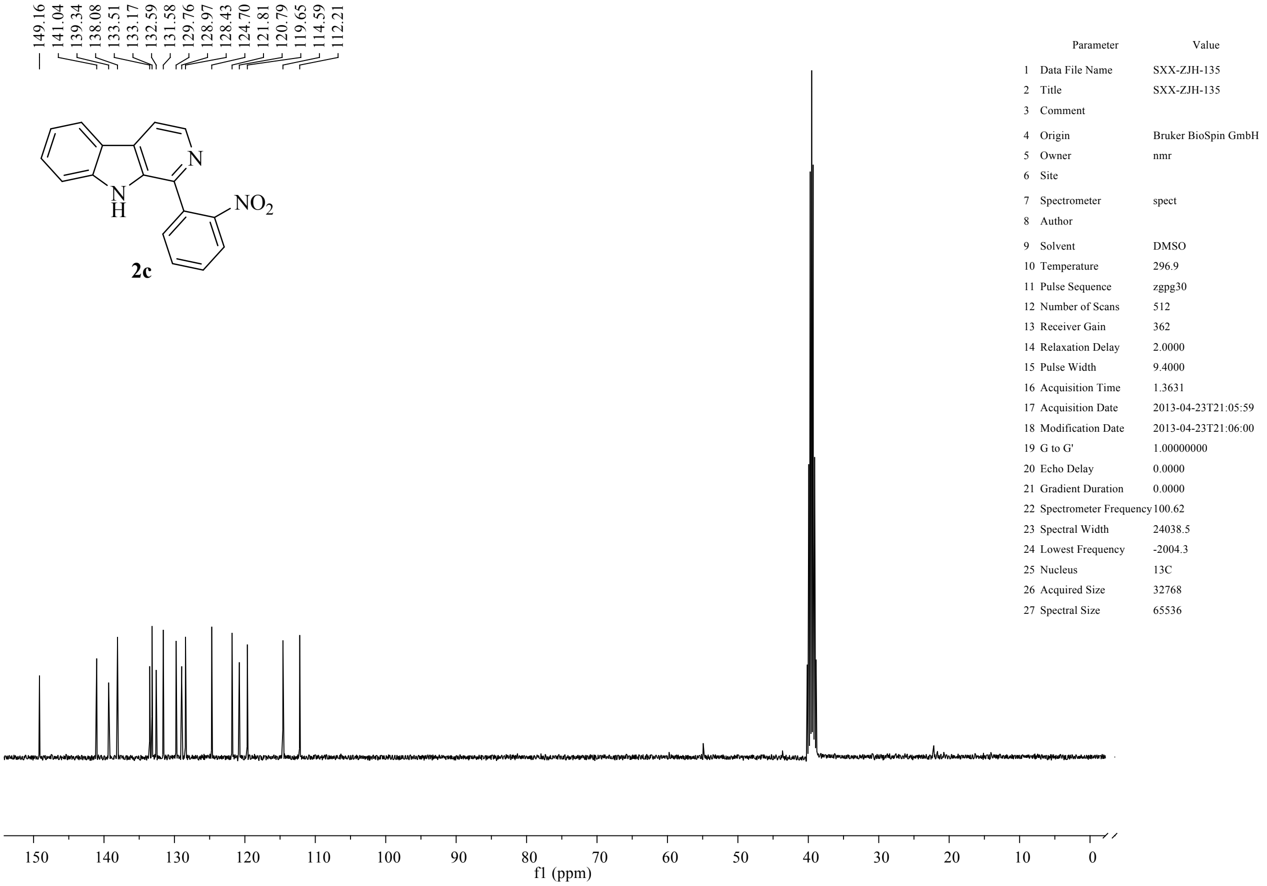

^{1}H NMR (DMSO-d_6, 400 MHz) spectrum of compound **2d**:

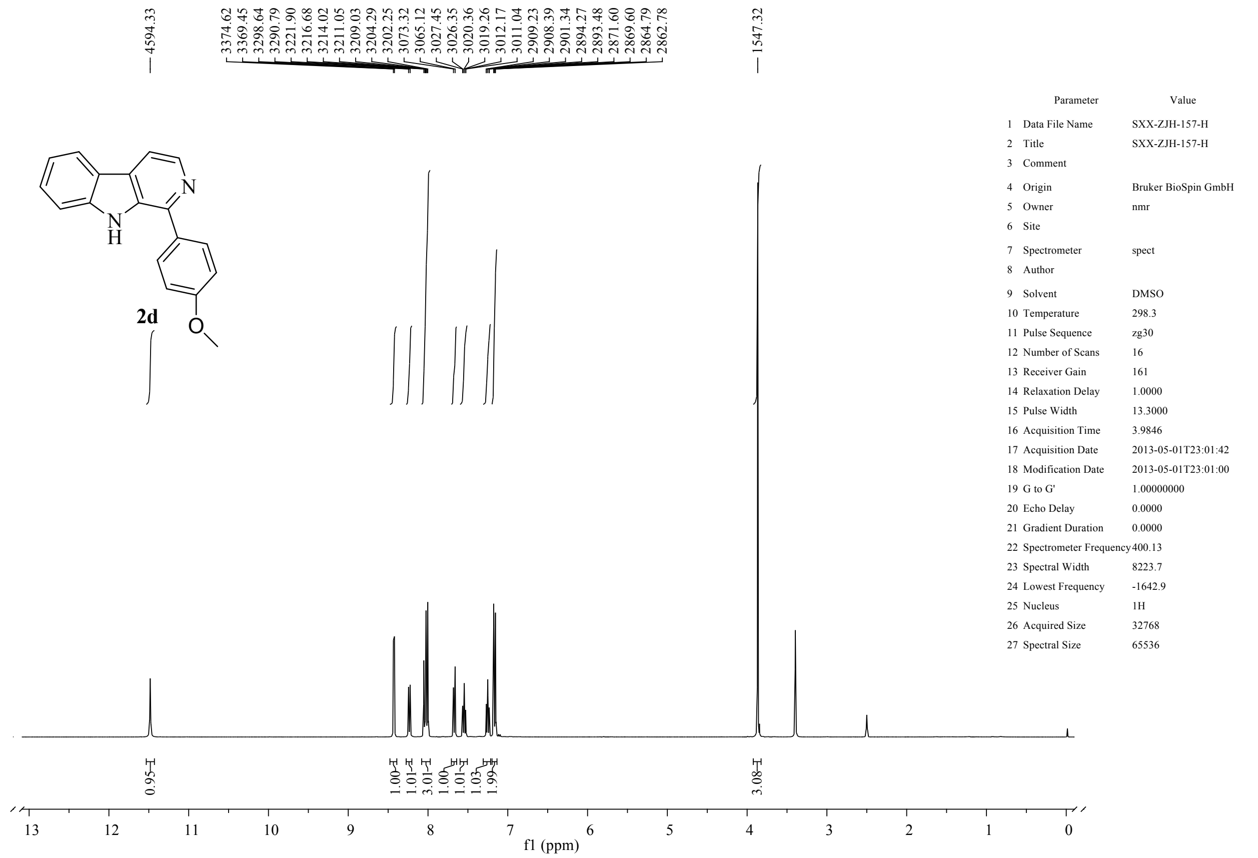

^{13}C NMR (DMSO-d_6, 100 MHz) spectrum of compound **2d**:

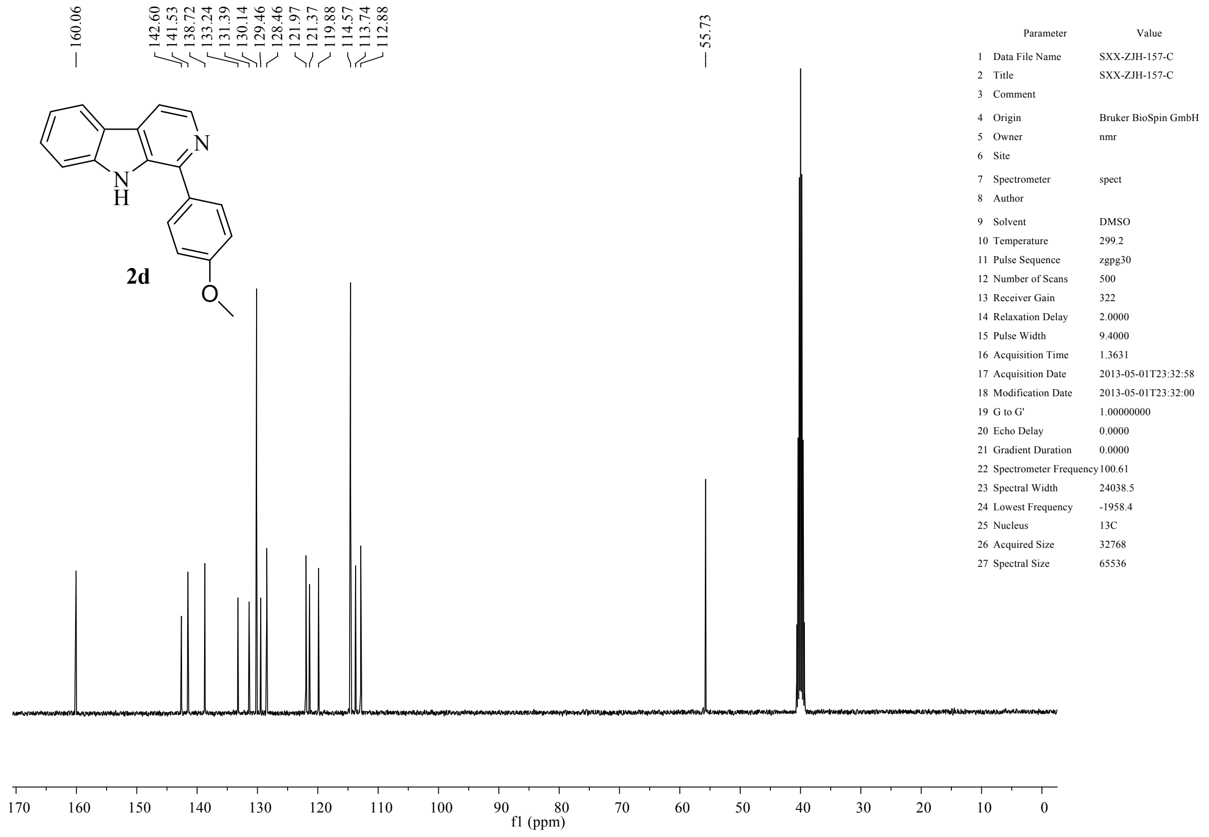

^{1}H NMR ($CDCl_3$, 400 MHz) spectrum of compound **2e**:

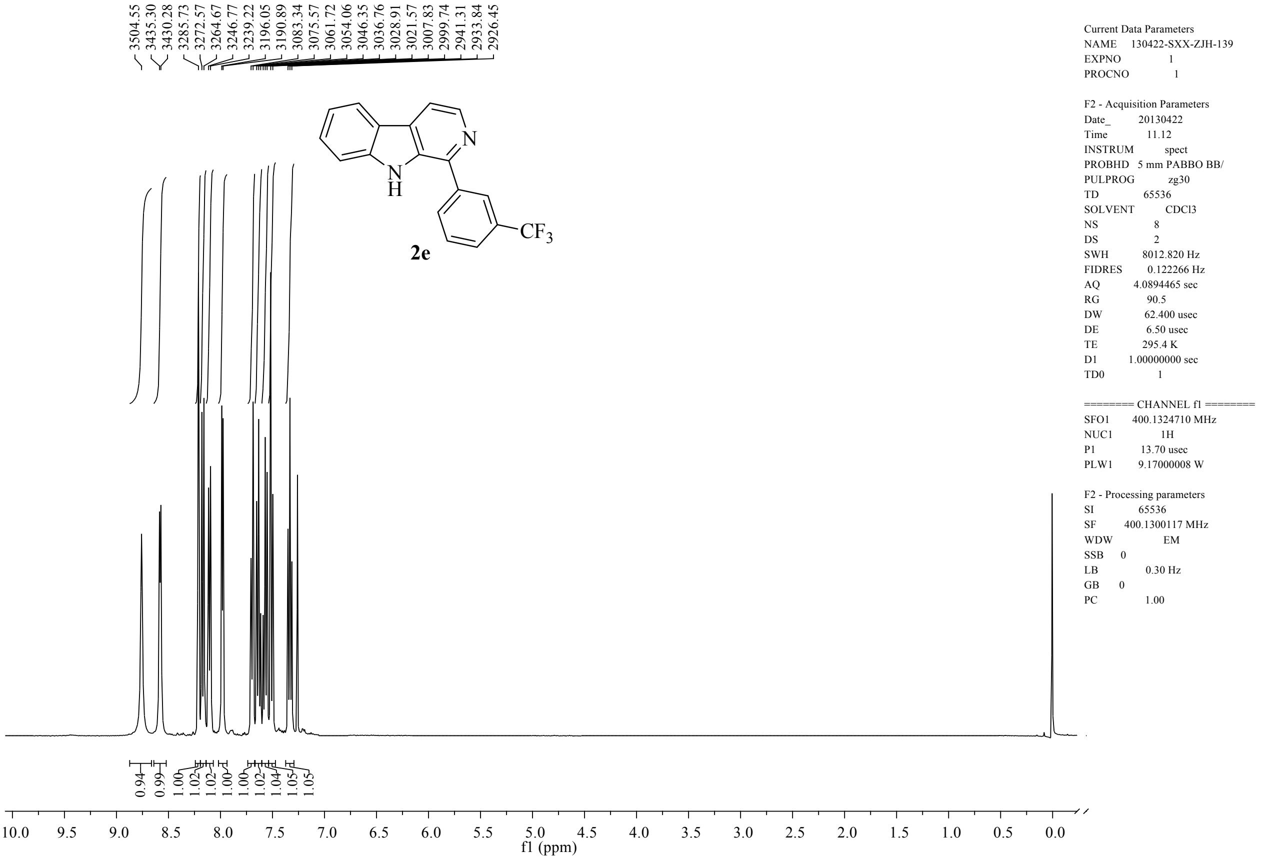

^{13}C NMR ($CDCl_3$, 100 MHz) spectrum of compound **2e**:

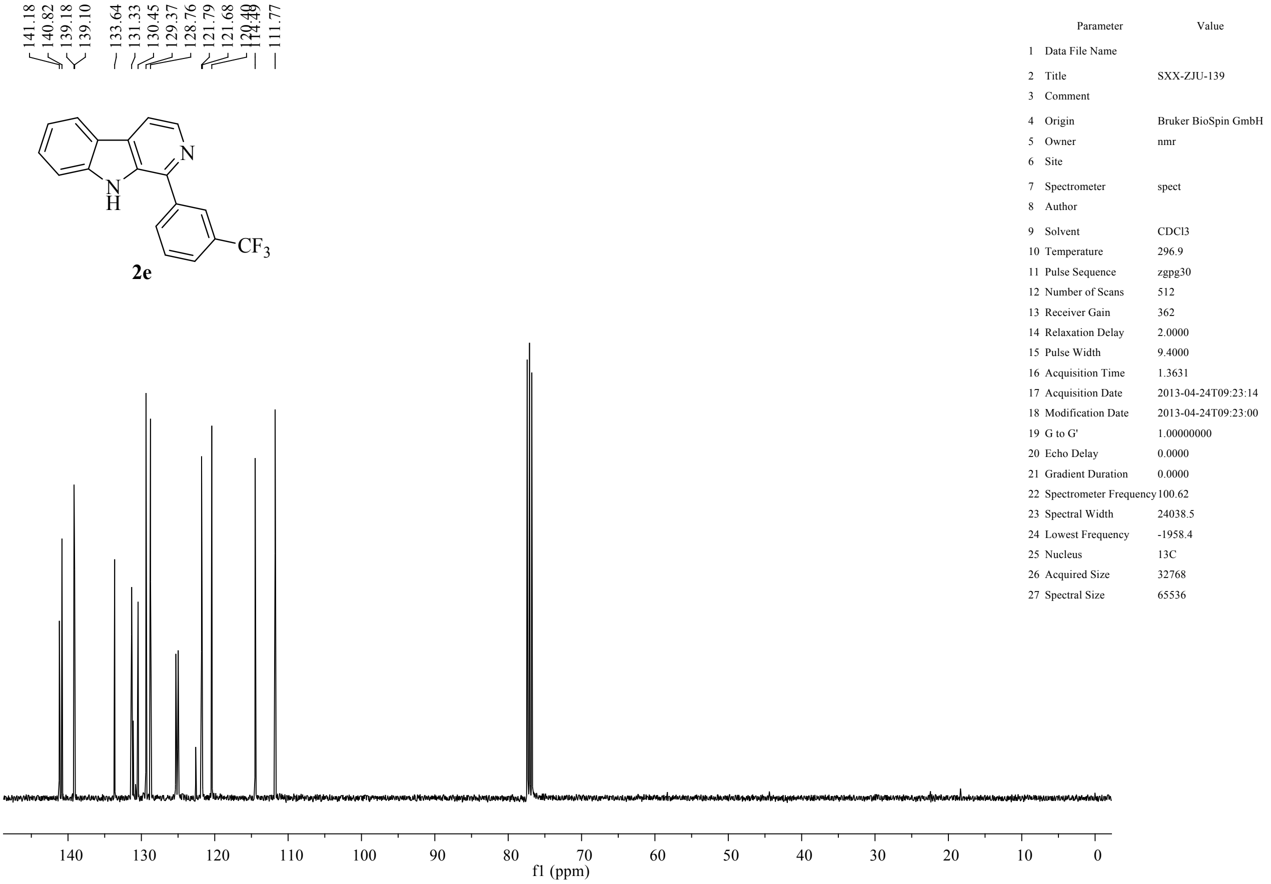

^{1}H NMR ($CDCl_3$, 400 MHz) spectrum of compound **2f**:

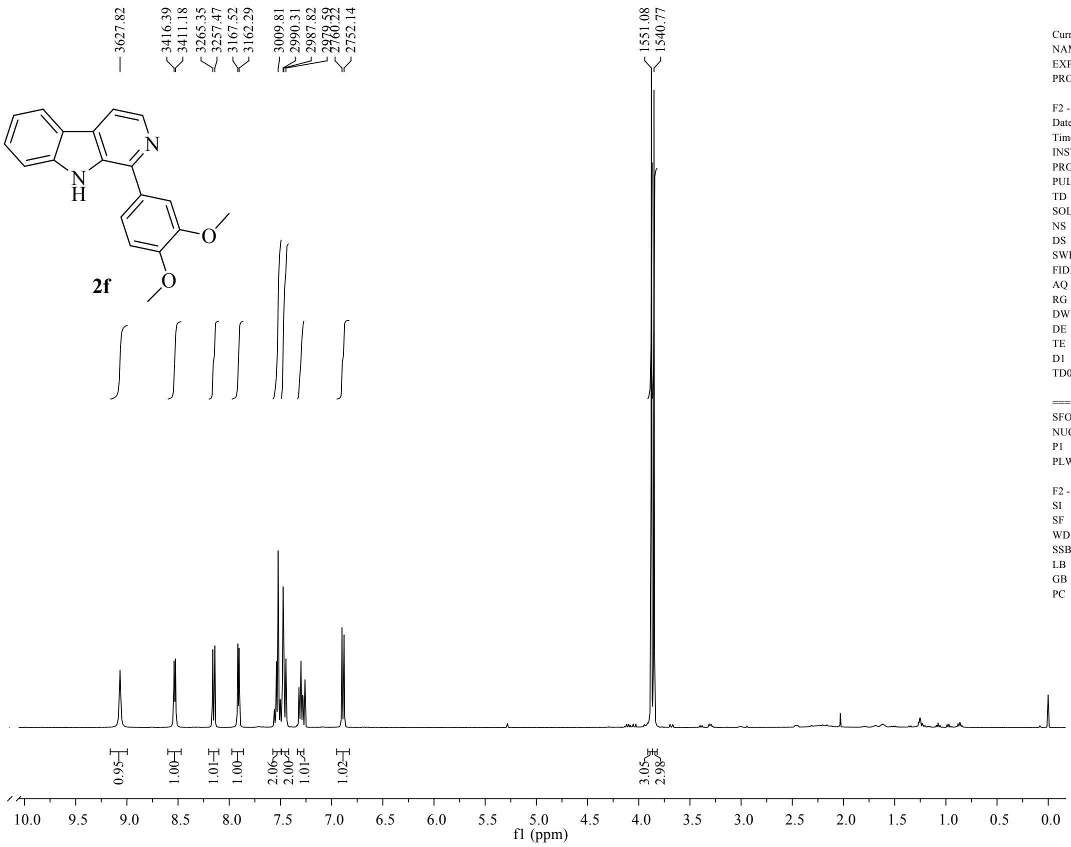

Current Data Parameters
NAME 130419-SXX-ZJH-136
EXPNO 1
PROCNO 1

F2 - Acquisition Parameters
Date_ 20130419
Time 11.17
INSTRUM spect
PROBHD 5 mm PABBO BB/
PULPROG zg30
TD 65536
SOLVENT CDCl3
NS 8
DS 2
SWH 8012.820 Hz
FIDRES 0.122266 Hz
AQ 4.0894465 sec
RG 64
DW 62.400 usec
DE 6.50 usec
TE 296.0 K
D1 1.00000000 sec
TD0 1

======== CHANNEL f1 ========
SFO1 400.1324710 MHz
NUC1 1H
P1 13.70 usec
PLW1 9.17000008 W

F2 - Processing parameters
SI 65536
SF 400.1300101 MHz
WDW EM
SSB 0
LB 0.30 Hz
GB 0
PC 1.00

^{13}C NMR (DMSO-d_6, 100 MHz) spectrum of compound **2f**:

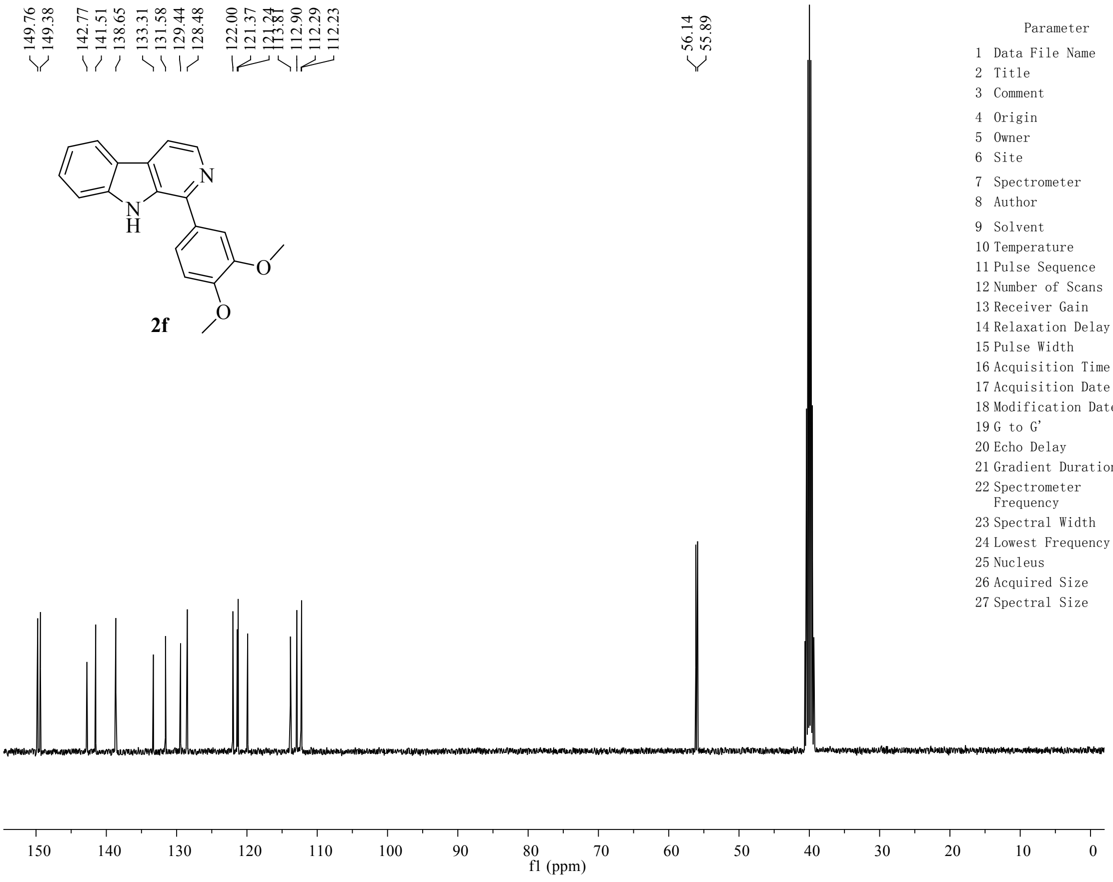

	Parameter	Value
1	Data File Name	SXX-ZJH-136
2	Title	SXX-ZJH-136
3	Comment	
4	Origin	Bruker BioSpin GmbH
5	Owner	nmr
6	Site	
7	Spectrometer	spect
8	Author	
9	Solvent	DMSO
10	Temperature	298.9
11	Pulse Sequence	zgpg30
12	Number of Scans	512
13	Receiver Gain	322
14	Relaxation Delay	2.0000
15	Pulse Width	9.4000
16	Acquisition Time	1.3631
17	Acquisition Date	2013-04-26T17:01:19
18	Modification Date	2013-04-26T17:01:00
19	G to G'	1.00000000
20	Echo Delay	0.0000
21	Gradient Duration	0.0000
22	Spectrometer Frequency	100.61
23	Spectral Width	24038.5
24	Lowest Frequency	-1958.4
25	Nucleus	13C
26	Acquired Size	32768
27	Spectral Size	65536

^{1}H NMR (DMSO-d_6, 400 MHz) spectrum of compound **2g**:

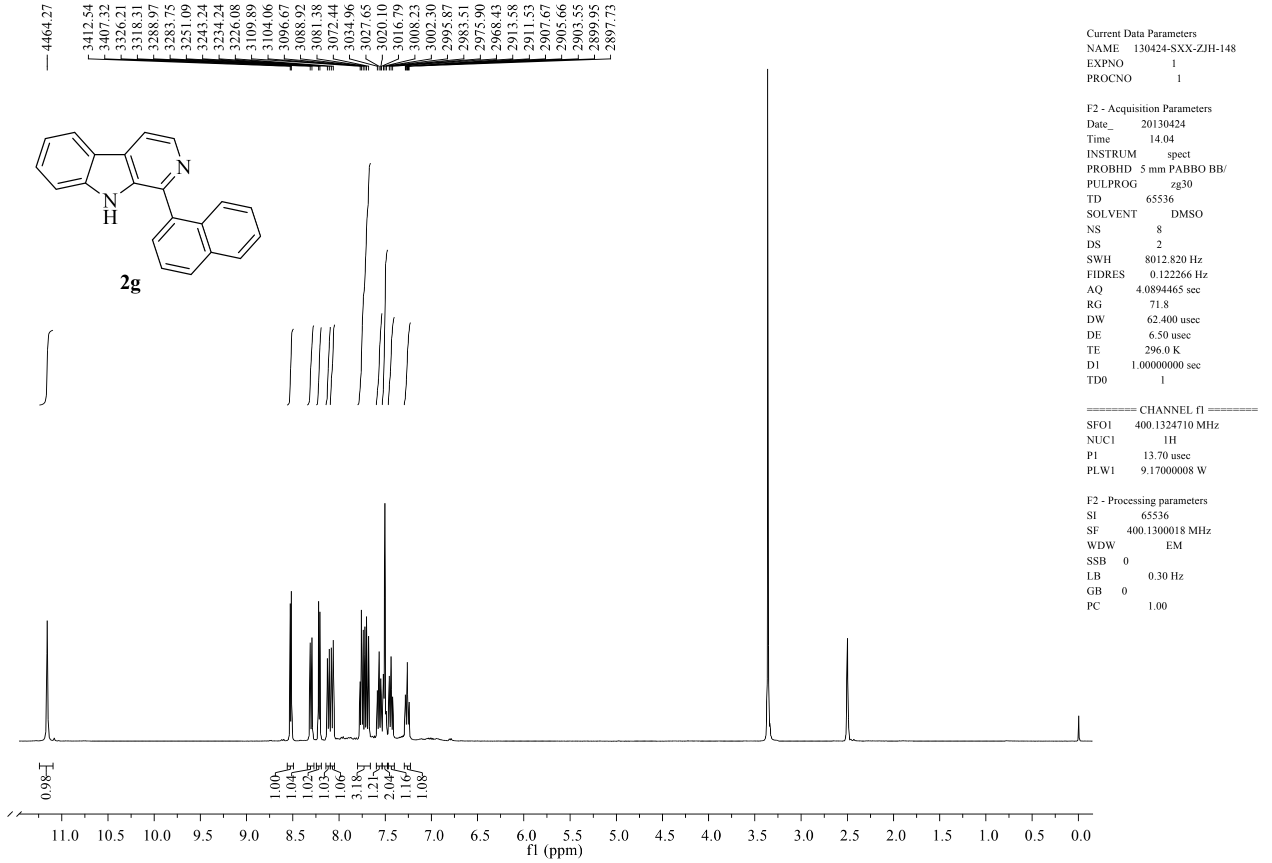

^{13}C NMR (DMSO-d_6, 100 MHz) spectrum of compound **2g**:

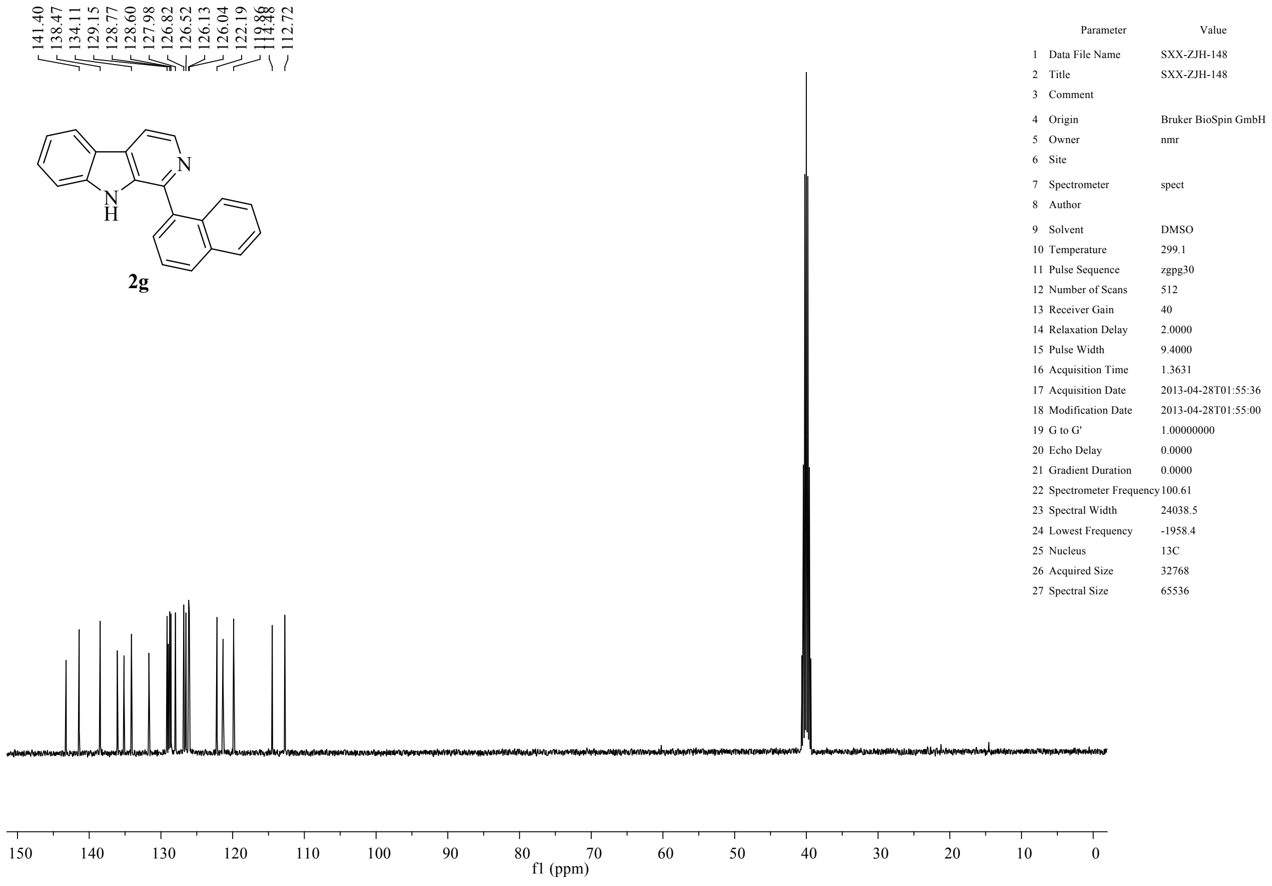

^{1}H NMR ($CDCl_3$, 400 MHz) spectrum of compound **2h**:

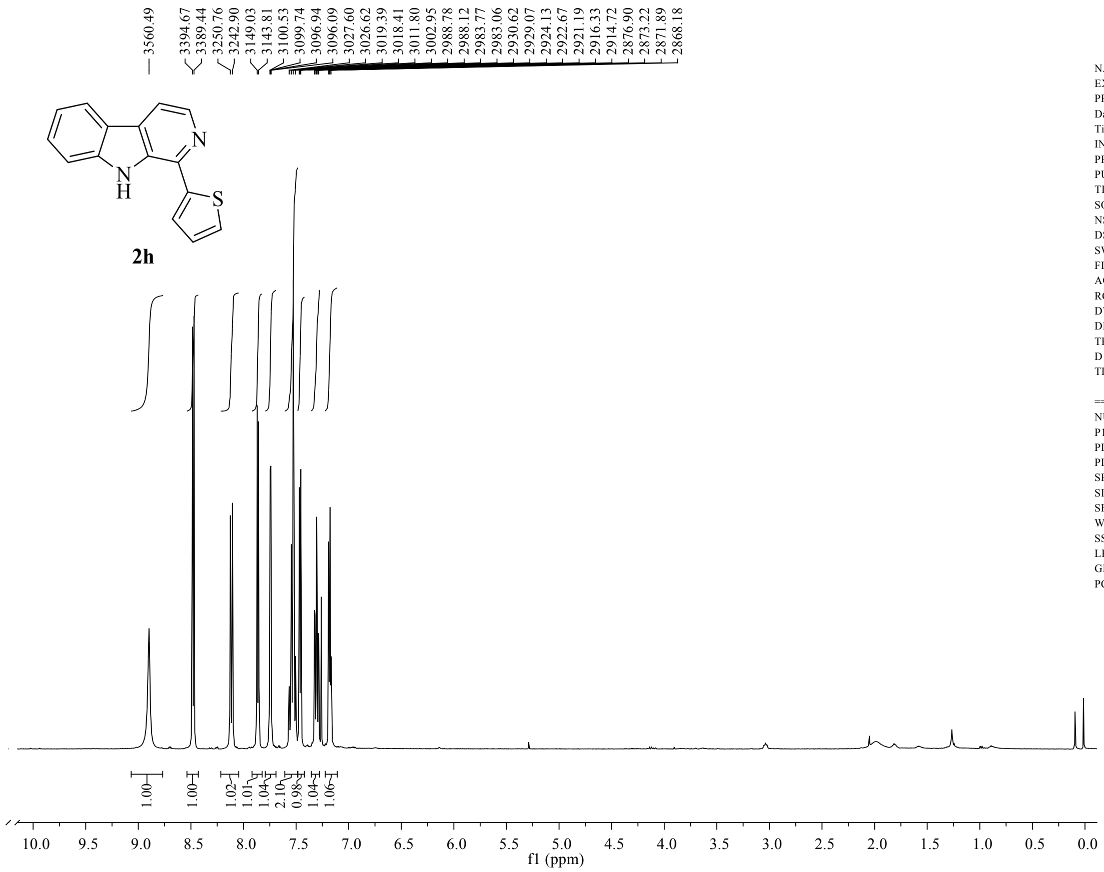

NAME SXX-ZJH-134
EXPNO 1
PROCNO 1
Date_ 20130419
Time 7.42
INSTRUM spect
PROBHD 5 mm PABBO BB-
PULPROG zg30
TD 65536
SOLVENT CDCl3
NS 16
DS 2
SWH 8223.685 Hz
FIDRES 0.125483 Hz
AQ 3.9846387 sec
RG 287
DW 60.800 usec
DE 6.50 usec
TE 298.3 K
D1 1.00000000 sec
TD0 1

======== CHANNEL f1 ========
NUC1 1H
P1 13.30 usec
PL1 -2.00 dB
PL1W 15.28731537 W
SFO1 400.1324710 MHz
SI 32768
SF 400.1300151 MHz
WDW EM
SSB 0
LB 0.30 Hz
GB 0
PC 1.00

^{13}C NMR ($CDCl_3$, 100 MHz) spectrum of compound **2h**:

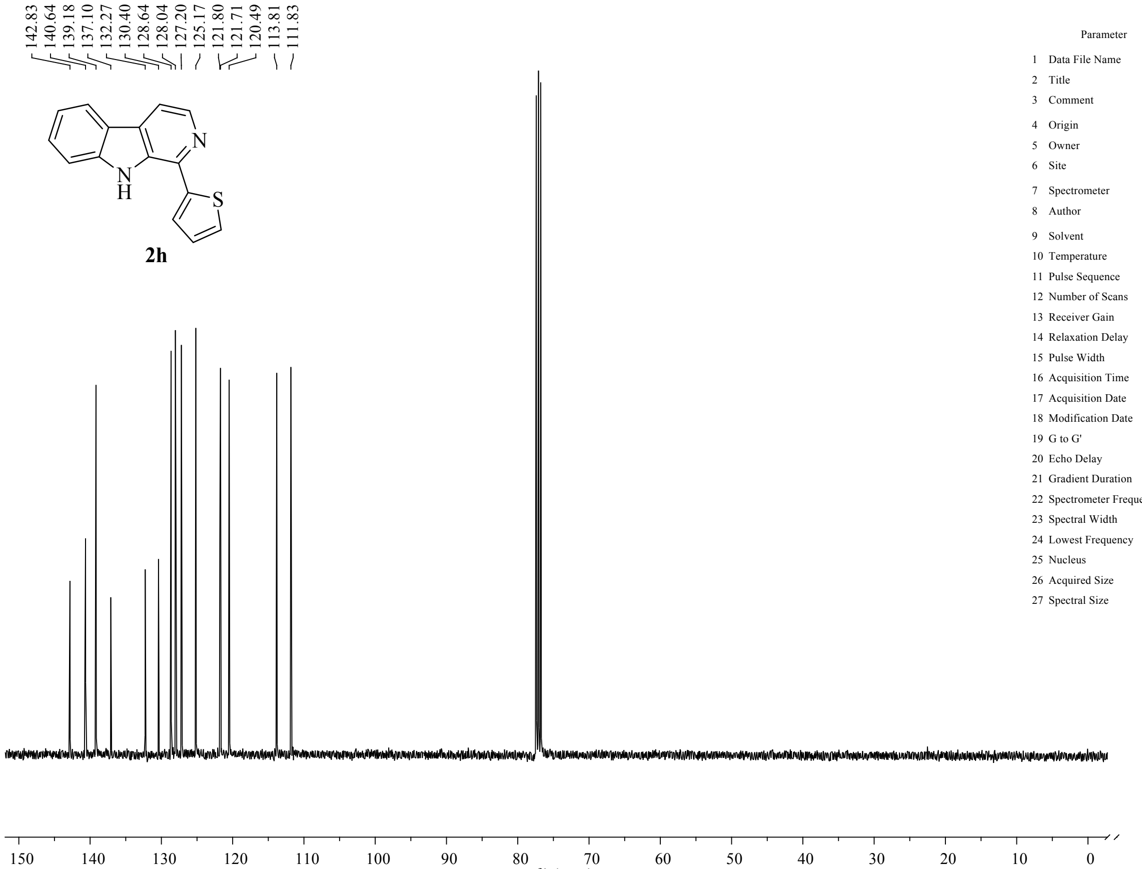

	Parameter	Value
1	Data File Name	SXX-ZJH-134
2	Title	SXX-ZJH-134
3	Comment	
4	Origin	Bruker BioSpin GmbH
5	Owner	nmr
6	Site	
7	Spectrometer	spect
8	Author	
9	Solvent	CDCl3
10	Temperature	296.5
11	Pulse Sequence	zgpg30
12	Number of Scans	500
13	Receiver Gain	362
14	Relaxation Delay	2.0000
15	Pulse Width	9.4000
16	Acquisition Time	1.3631
17	Acquisition Date	2013-04-23T08:58:57
18	Modification Date	2013-04-23T08:59:00
19	G to G'	1.00000000
20	Echo Delay	0.0000
21	Gradient Duration	0.0000
22	Spectrometer Frequency	100.62
23	Spectral Width	24038.5
24	Lowest Frequency	-1958.4
25	Nucleus	13C
26	Acquired Size	32768
27	Spectral Size	65536

^{1}H NMR ($CDCl_3$, 400 MHz) spectrum of compound **2i**:

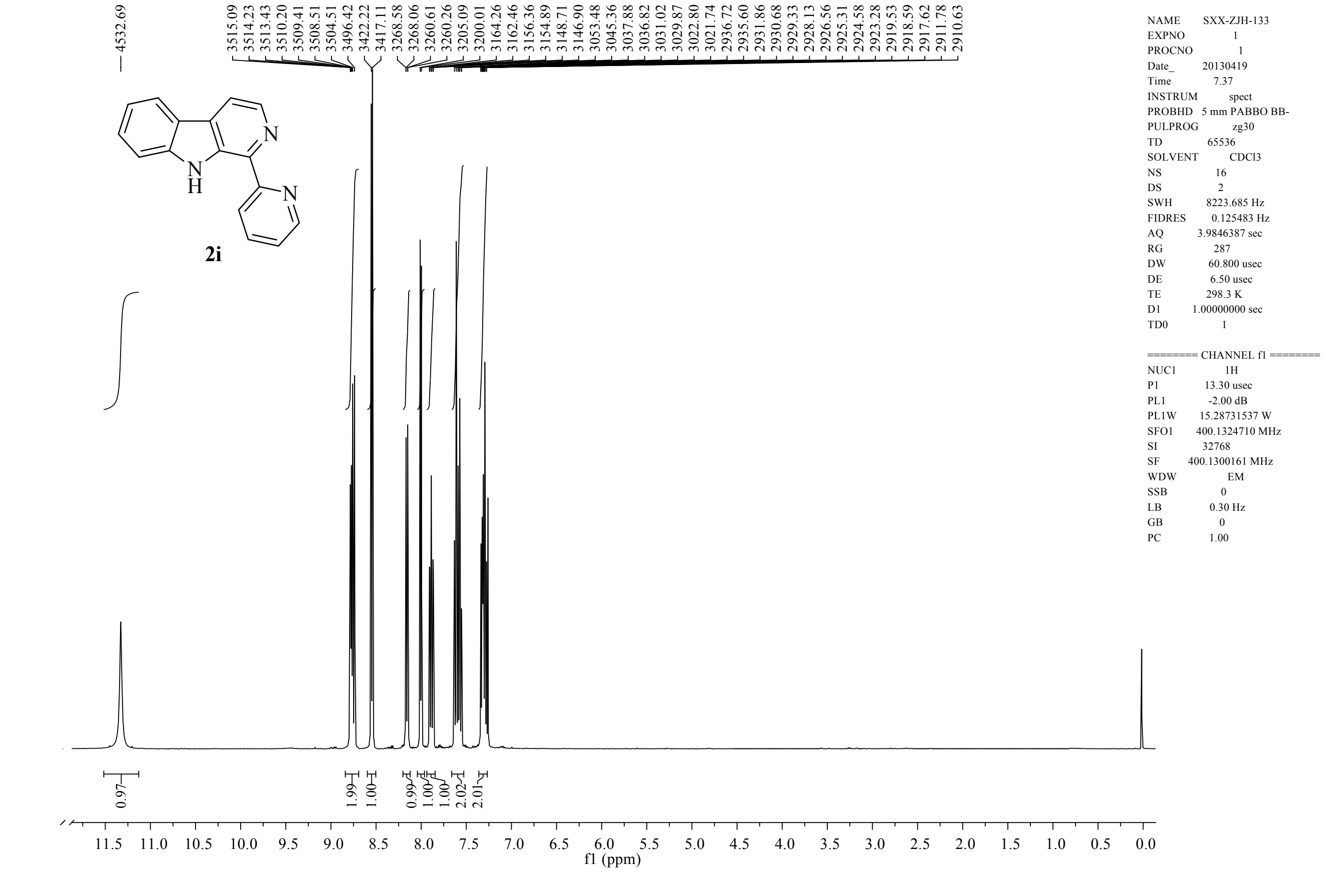

^{13}C NMR ($CDCl_3$, 100 MHz) spectrum of compound **2i**:

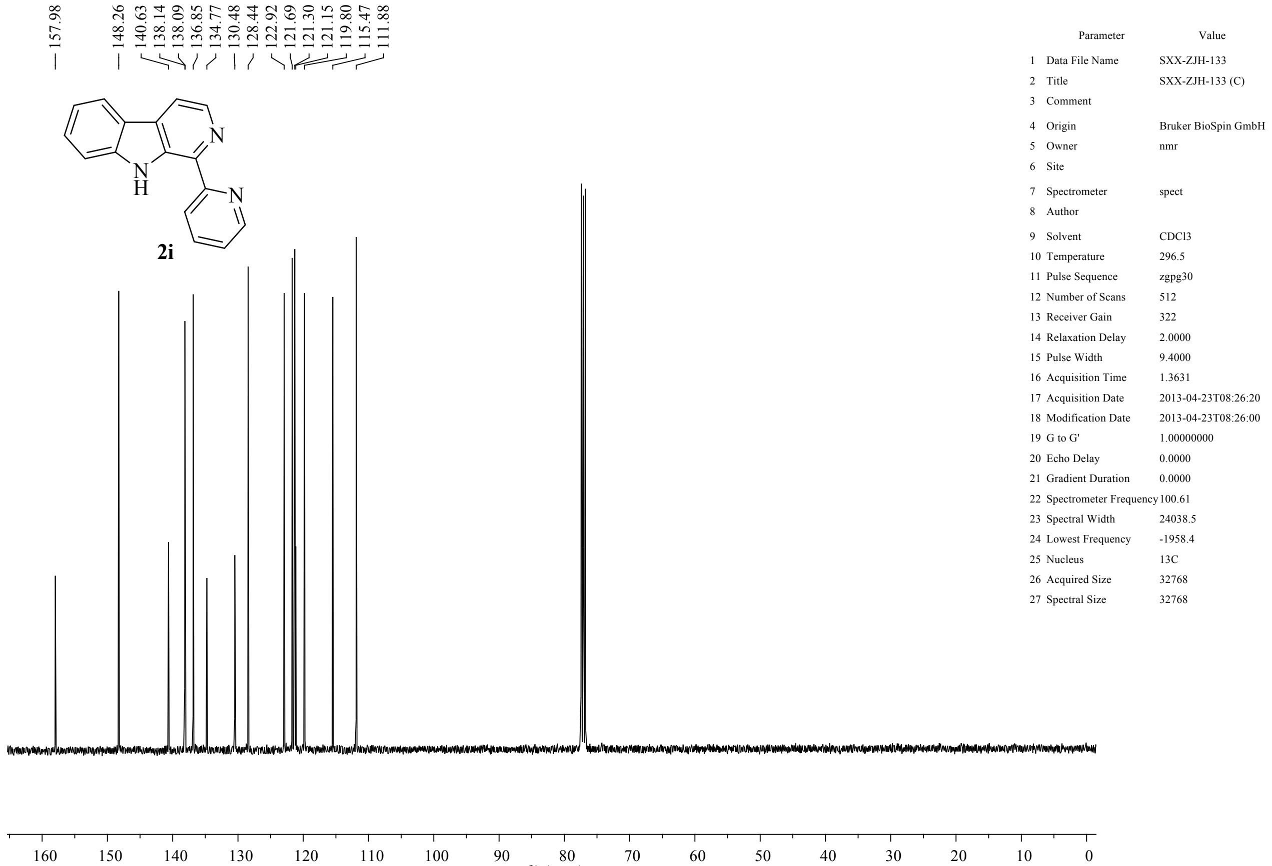

^{1}H NMR ($CDCl_3$, 400 MHz) spectrum of compound **2j**:

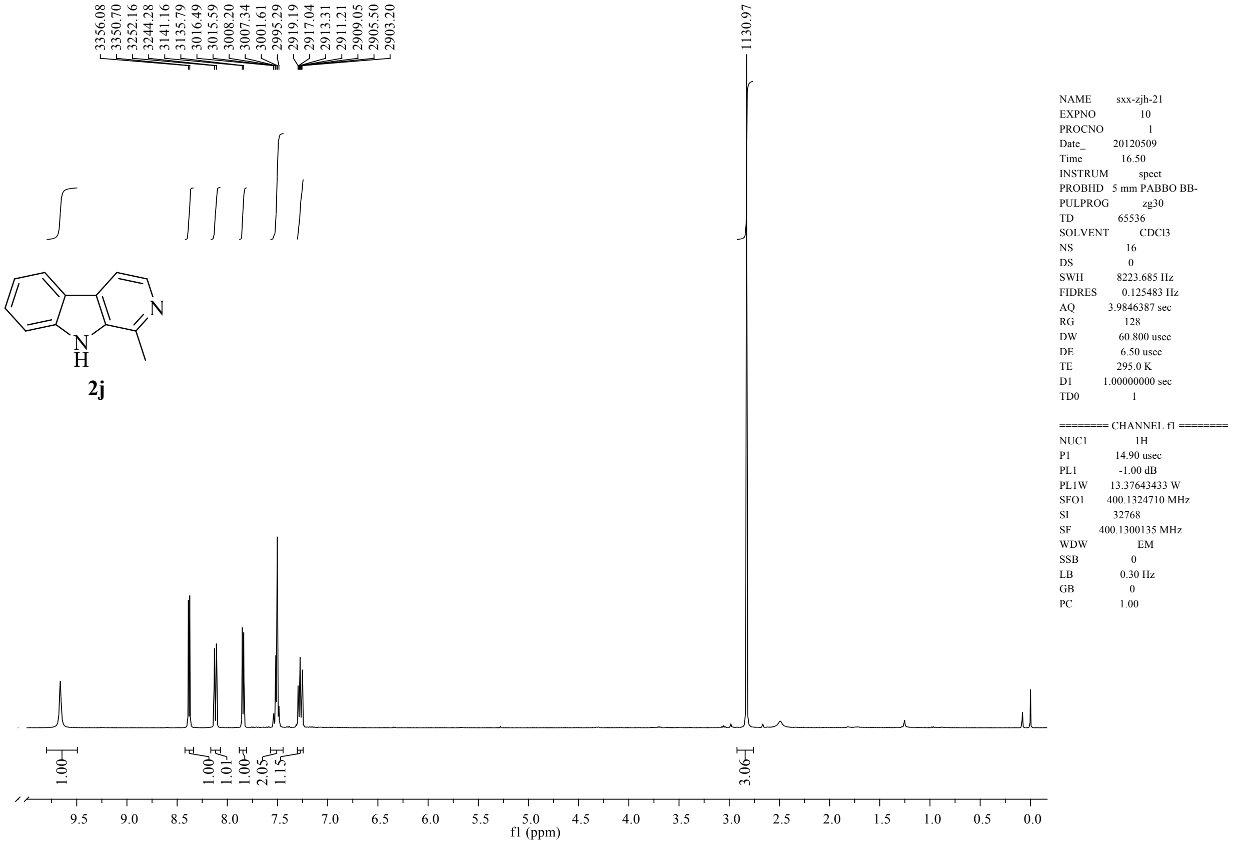

^{13}C NMR ($CDCl_3$, 100 MHz) spectrum of compound **2j**:

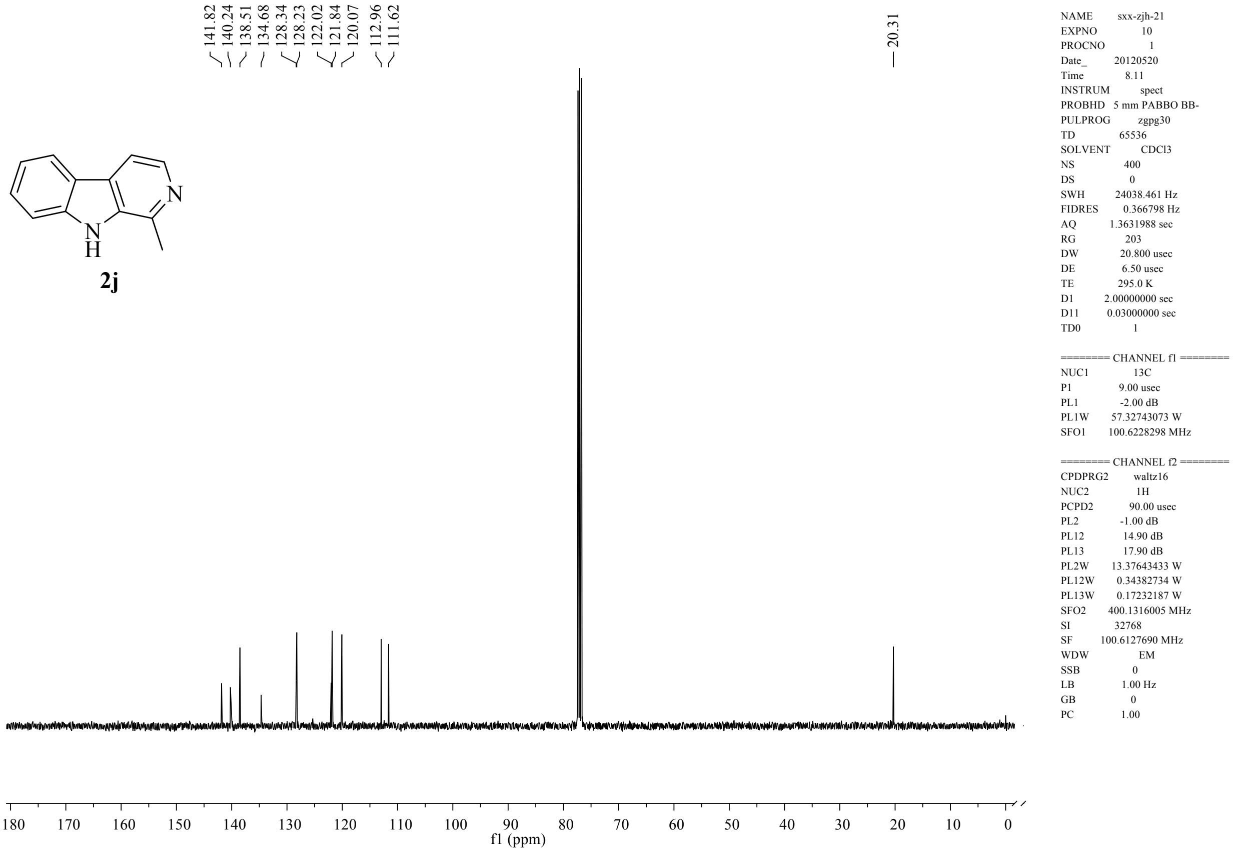

^{1}H NMR (DMSO-d_6, 400 MHz) spectrum of compound **2k**:

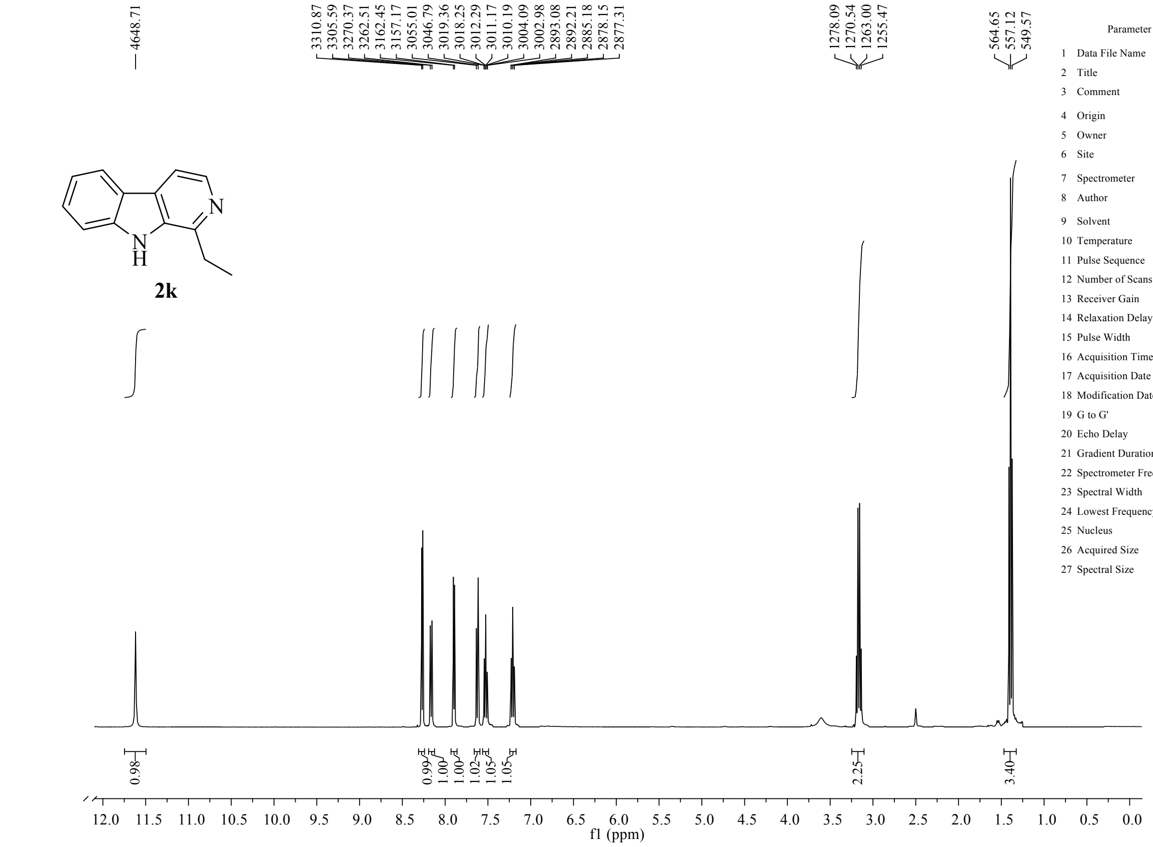

	Parameter	Value
1	Data File Name	SXX-ZJH-156-H
2	Title	SXX-ZJH-156-H
3	Comment	
4	Origin	Bruker BioSpin GmbH
5	Owner	nmr
6	Site	
7	Spectrometer	spect
8	Author	
9	Solvent	DMSO
10	Temperature	298.4
11	Pulse Sequence	zg30
12	Number of Scans	16
13	Receiver Gain	81
14	Relaxation Delay	1.0000
15	Pulse Width	13.3000
16	Acquisition Time	3.9846
17	Acquisition Date	2013-05-01T22:26:12
18	Modification Date	2013-05-01T22:26:00
19	G to G'	1.00000000
20	Echo Delay	0.0000
21	Gradient Duration	0.0000
22	Spectrometer Frequency	400.13
23	Spectral Width	8223.7
24	Lowest Frequency	-1642.9
25	Nucleus	1H
26	Acquired Size	32768
27	Spectral Size	65536

^{13}C NMR (DMSO-d_6, 100 MHz) spectrum of compound **2k**:

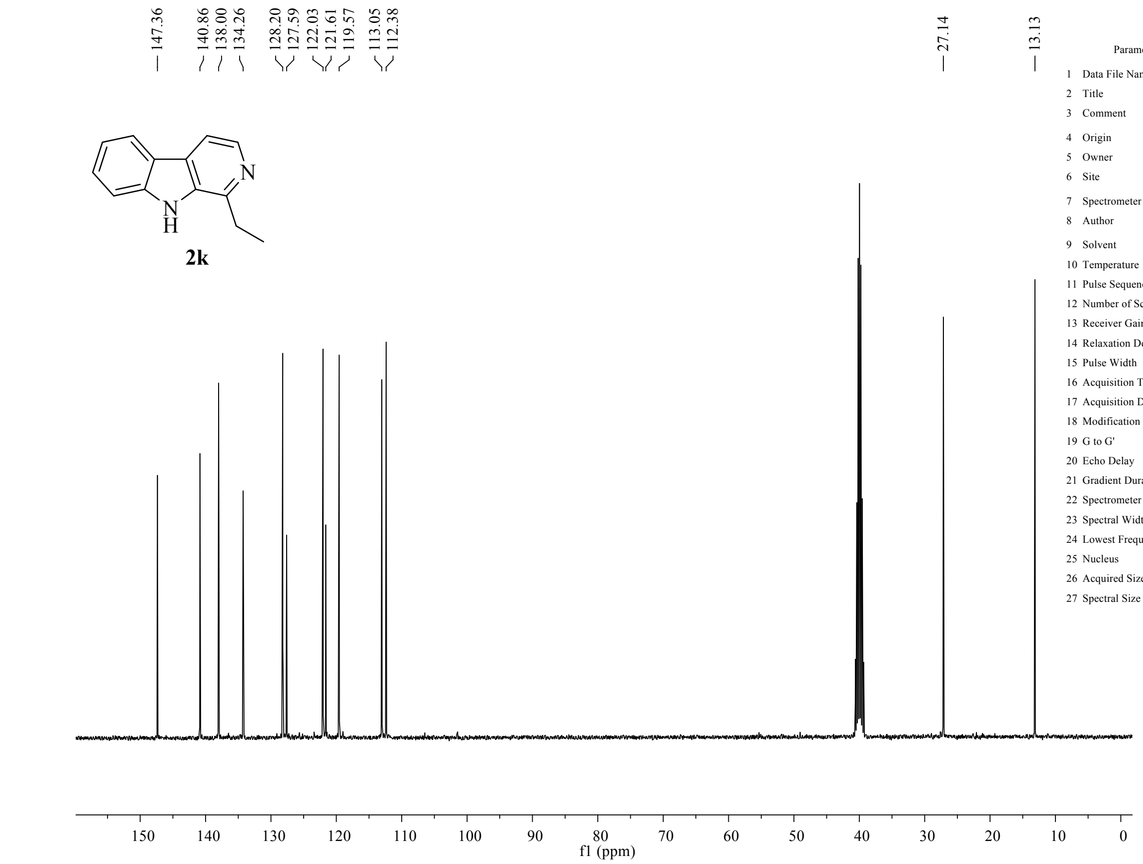

	Parameter	Value
1	Data File Name	SXX-ZJH-156-C
2	Title	SXX-ZJH-156-C
3	Comment	
4	Origin	Bruker BioSpin GmbH
5	Owner	nmr
6	Site	
7	Spectrometer	spect
8	Author	
9	Solvent	DMSO
10	Temperature	299.2
11	Pulse Sequence	zgpg30
12	Number of Scans	500
13	Receiver Gain	322
14	Relaxation Delay	2.0000
15	Pulse Width	9.4000
16	Acquisition Time	1.3631
17	Acquisition Date	2013-05-01T22:57:26
18	Modification Date	2013-05-01T22:57:00
19	G to G'	1.00000000
20	Echo Delay	0.0000
21	Gradient Duration	0.0000
22	Spectrometer Frequency	100.61
23	Spectral Width	24038.5
24	Lowest Frequency	-1958.4
25	Nucleus	13C
26	Acquired Size	32768
27	Spectral Size	65536

^{1}H NMR ($CDCl_3$, 400 MHz) spectrum of compound **2l**:

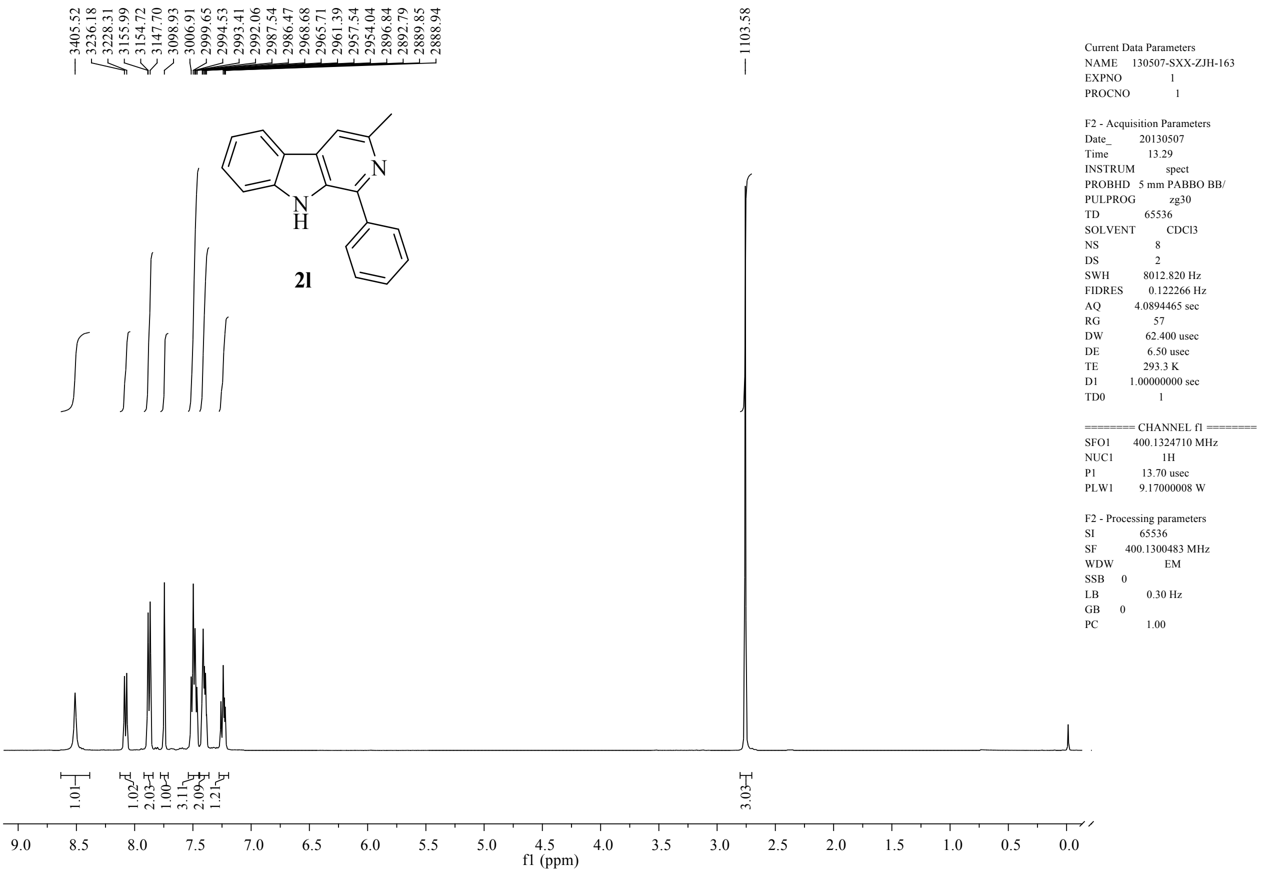

^{13}C NMR ($CDCl_3$, 100 MHz) spectrum of compound **2l**:

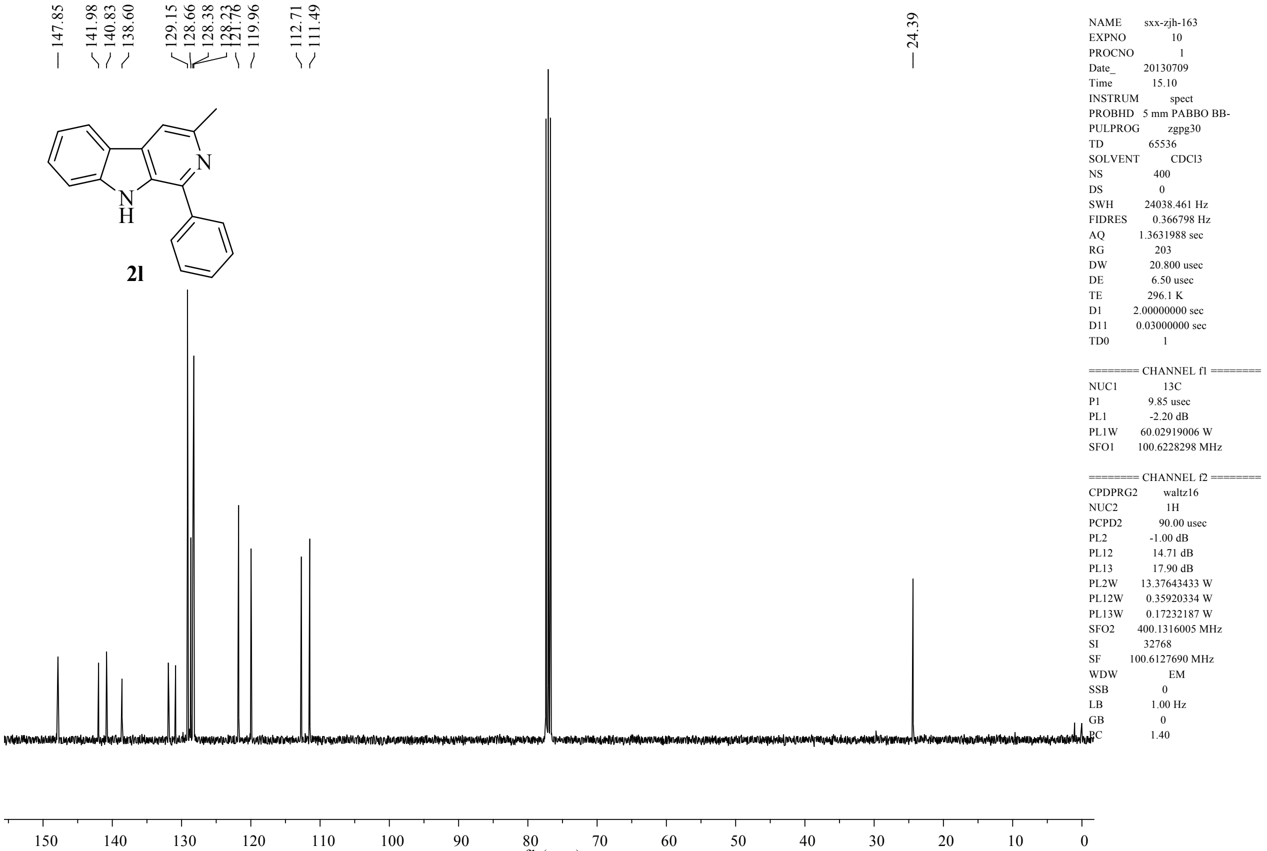

^{1}H NMR ($CDCl_3$, 400 MHz) spectrum of compound **2m**:

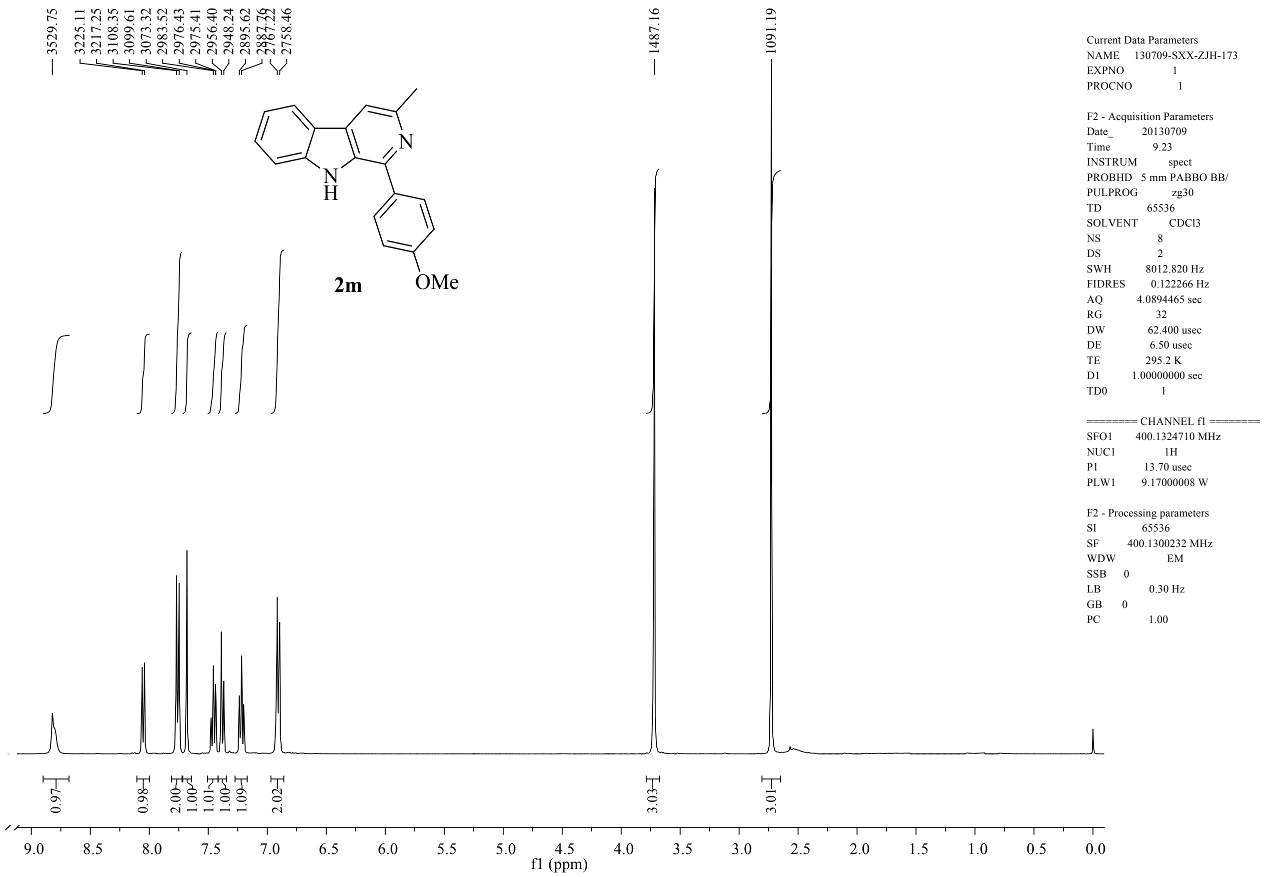

^{13}C NMR ($CDCl_3$, 100 MHz) spectrum of compound **2m**:

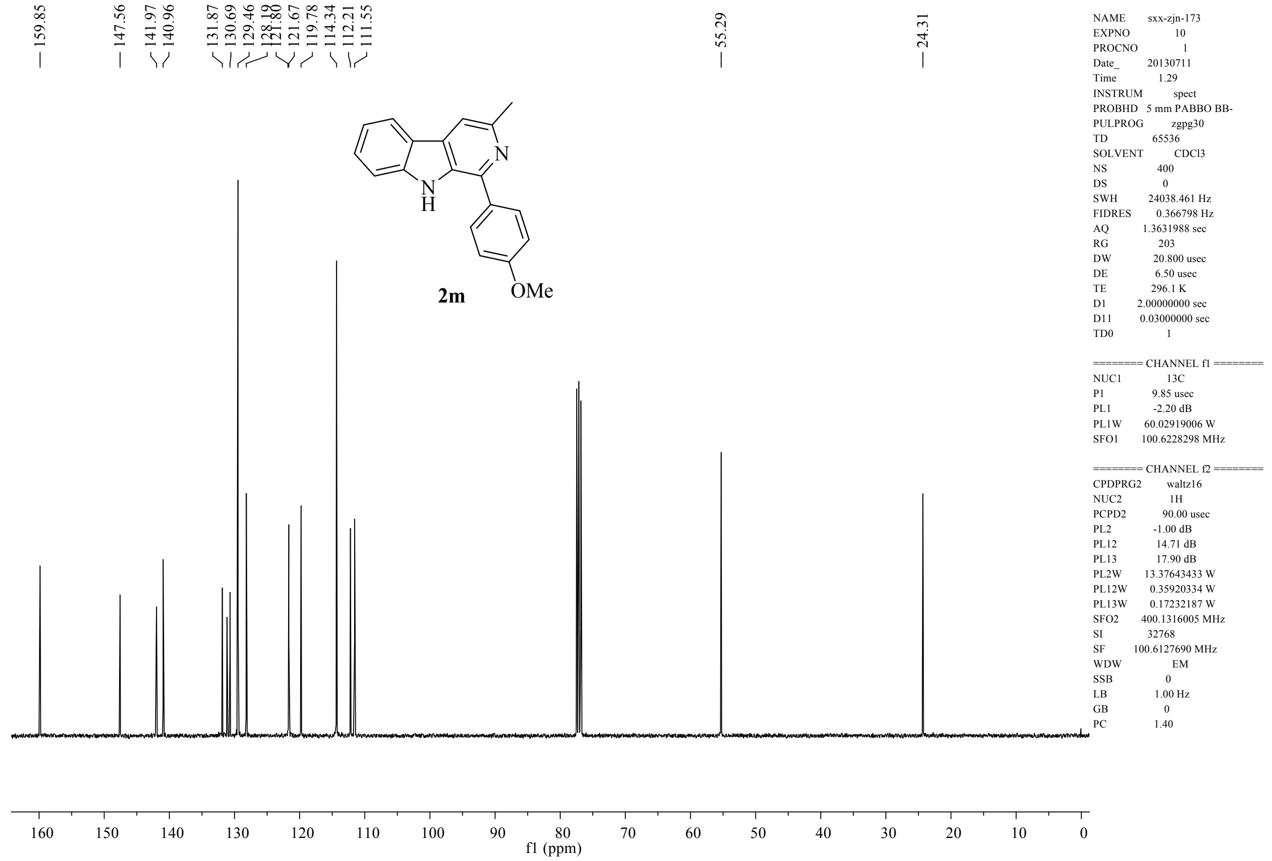

^{1}H NMR ($CDCl_3$, 400 MHz) spectrum of compound **2n**:

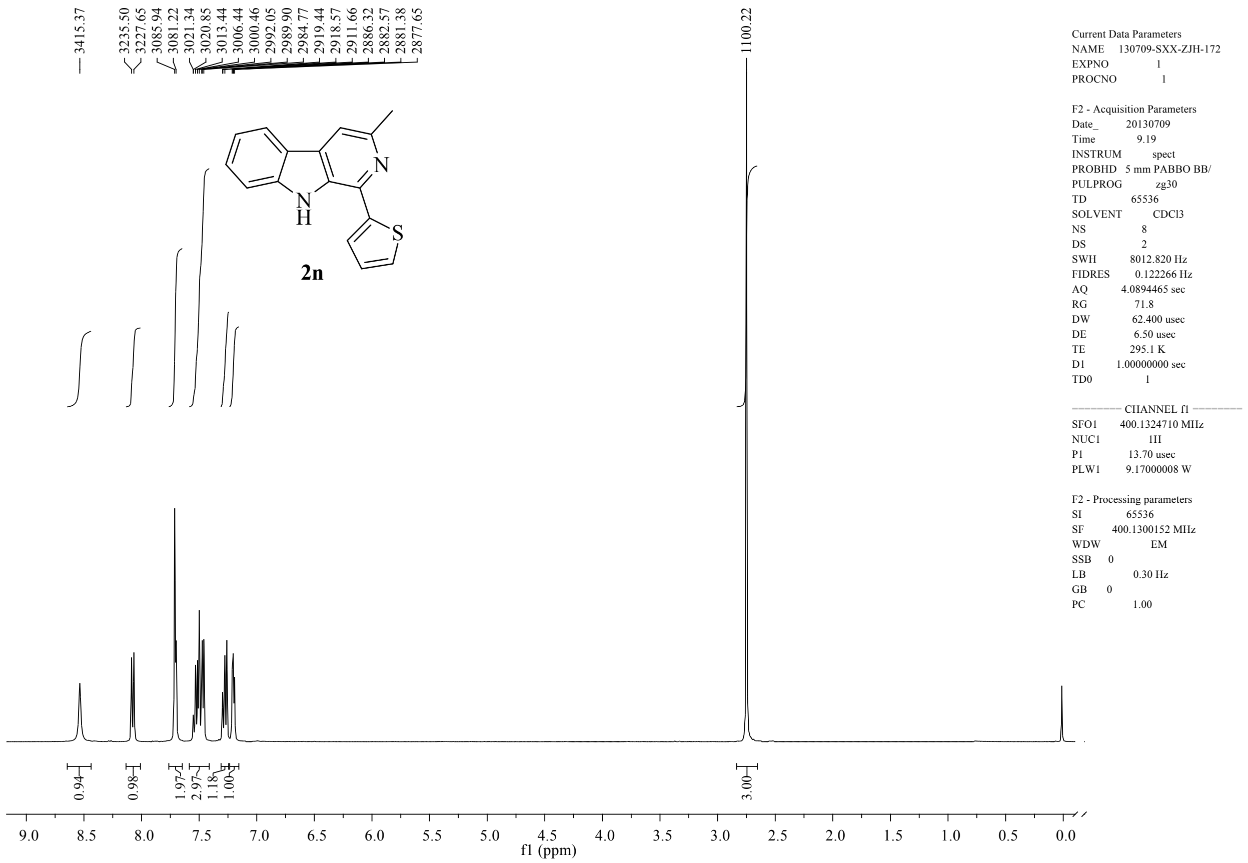

^{13}C NMR ($CDCl_3$, 100 MHz) spectrum of compound **2n**:

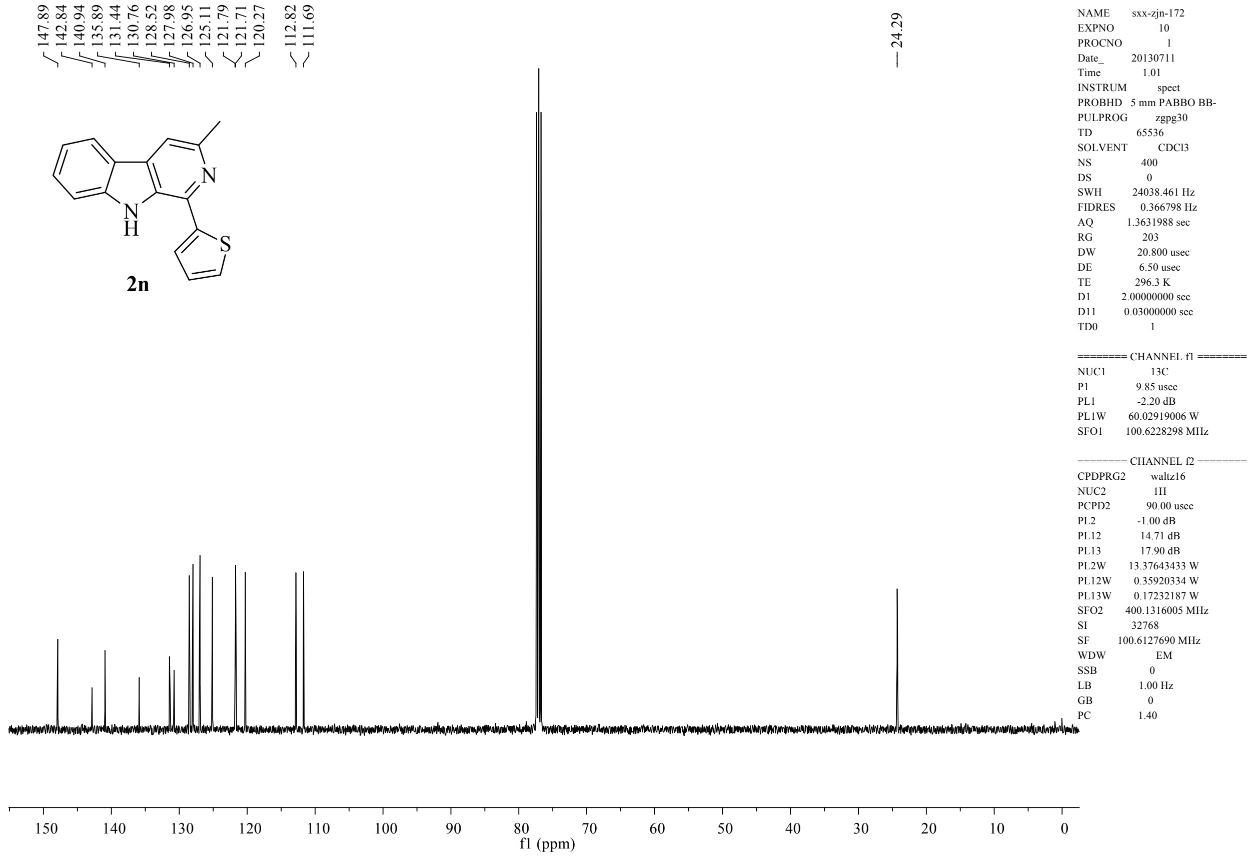

^{1}H NMR (DMSO-d_6, 400 MHz) spectrum of compound **2o**:

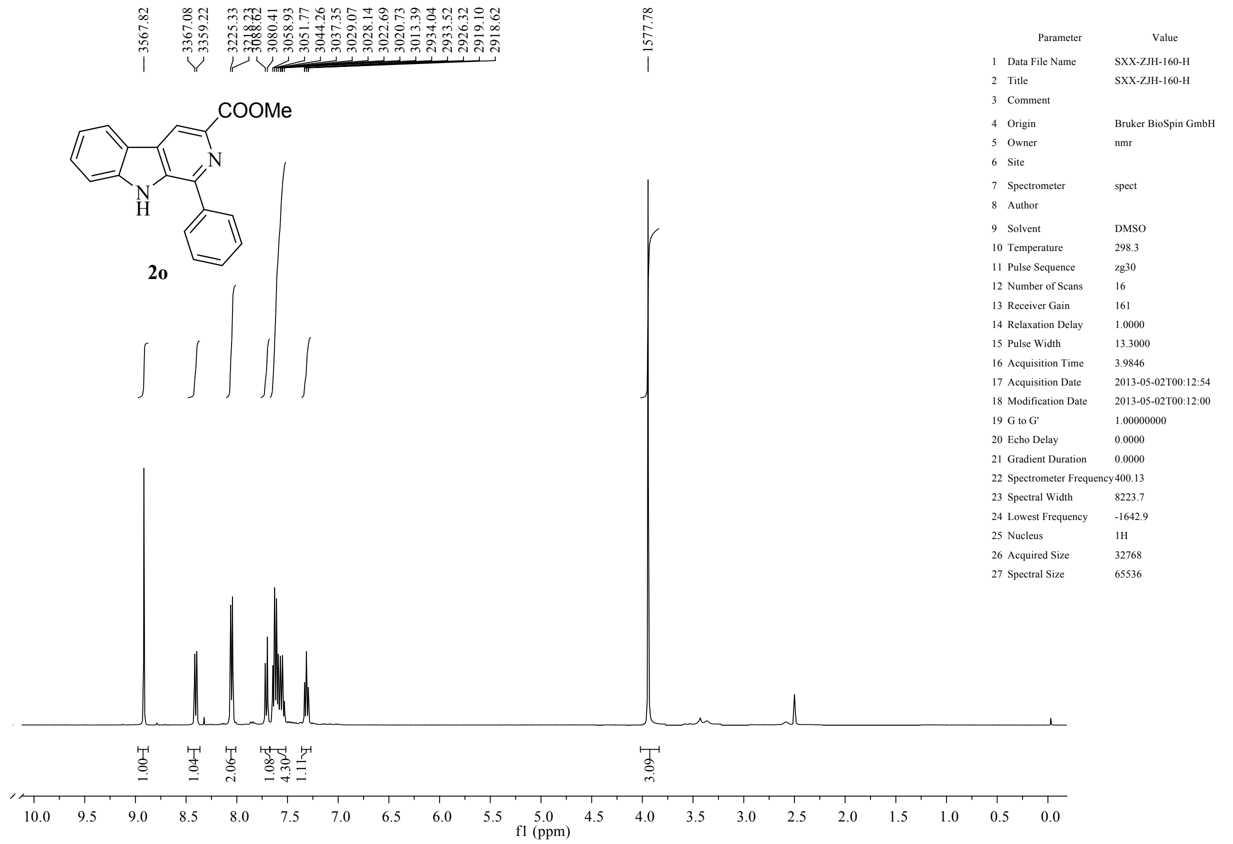

^{13}C NMR (DMSO-d_6, 100 MHz) spectrum of compound **2o**:

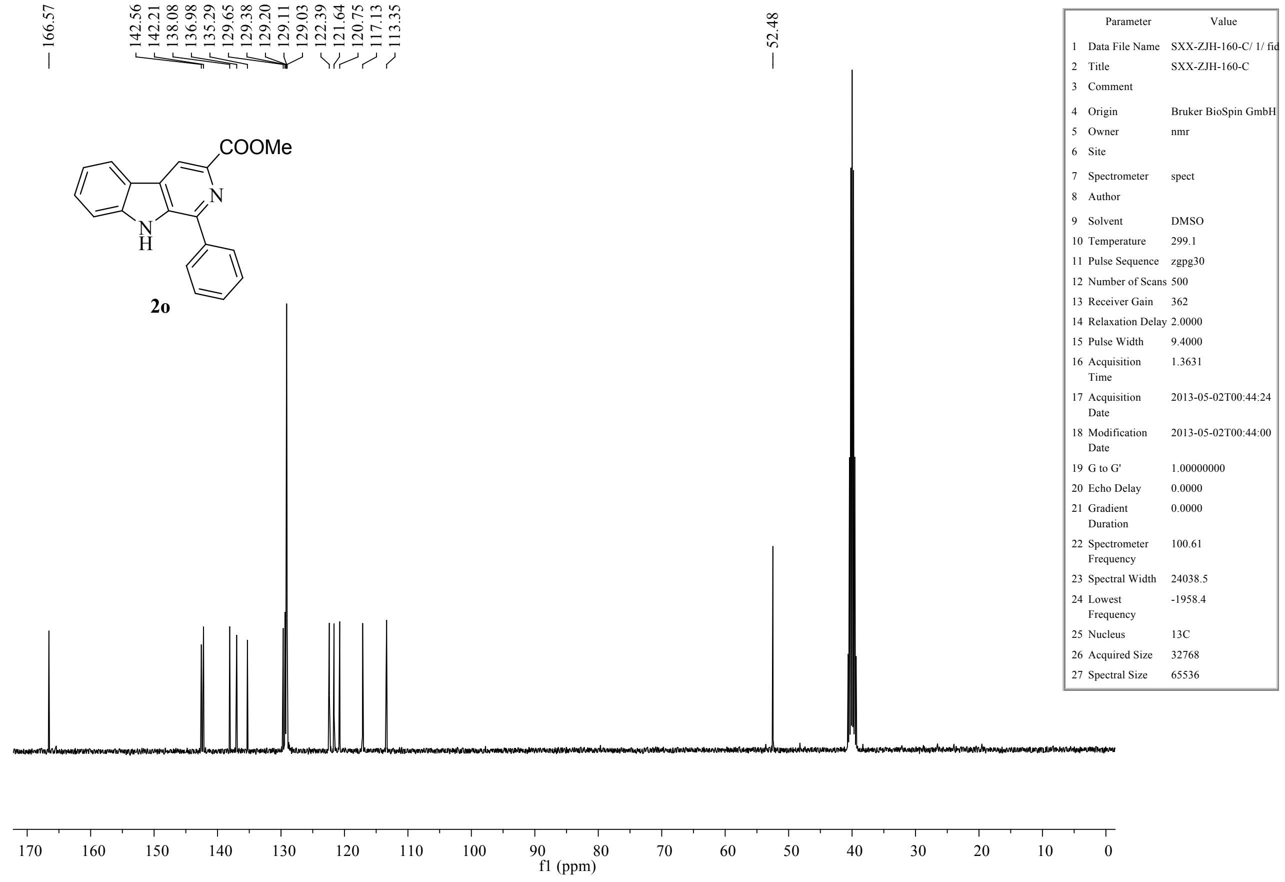

^{1}H NMR (DMSO-d_6, 400 MHz) spectrum of compound **2p**:

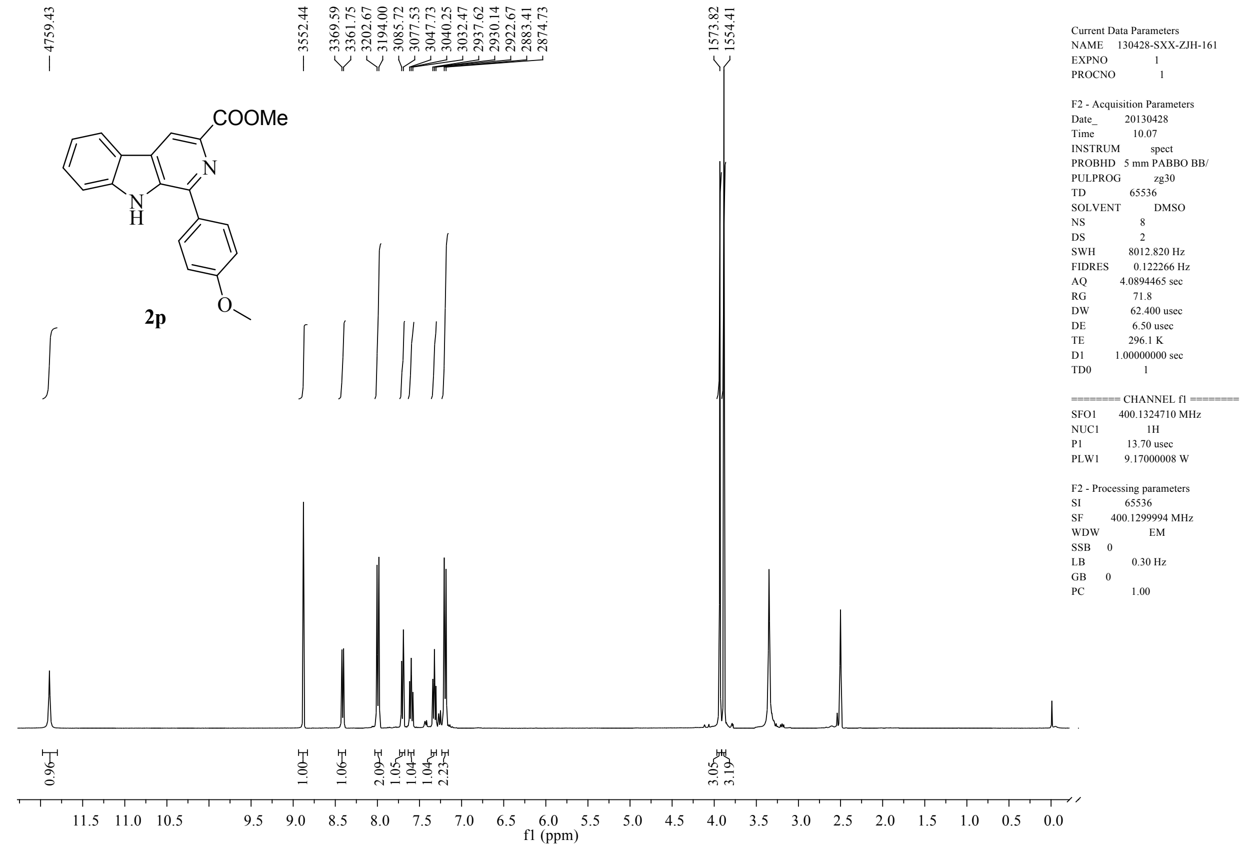

^{13}C NMR (DMSO-d_6, 100 MHz) spectrum of compound **2p**:

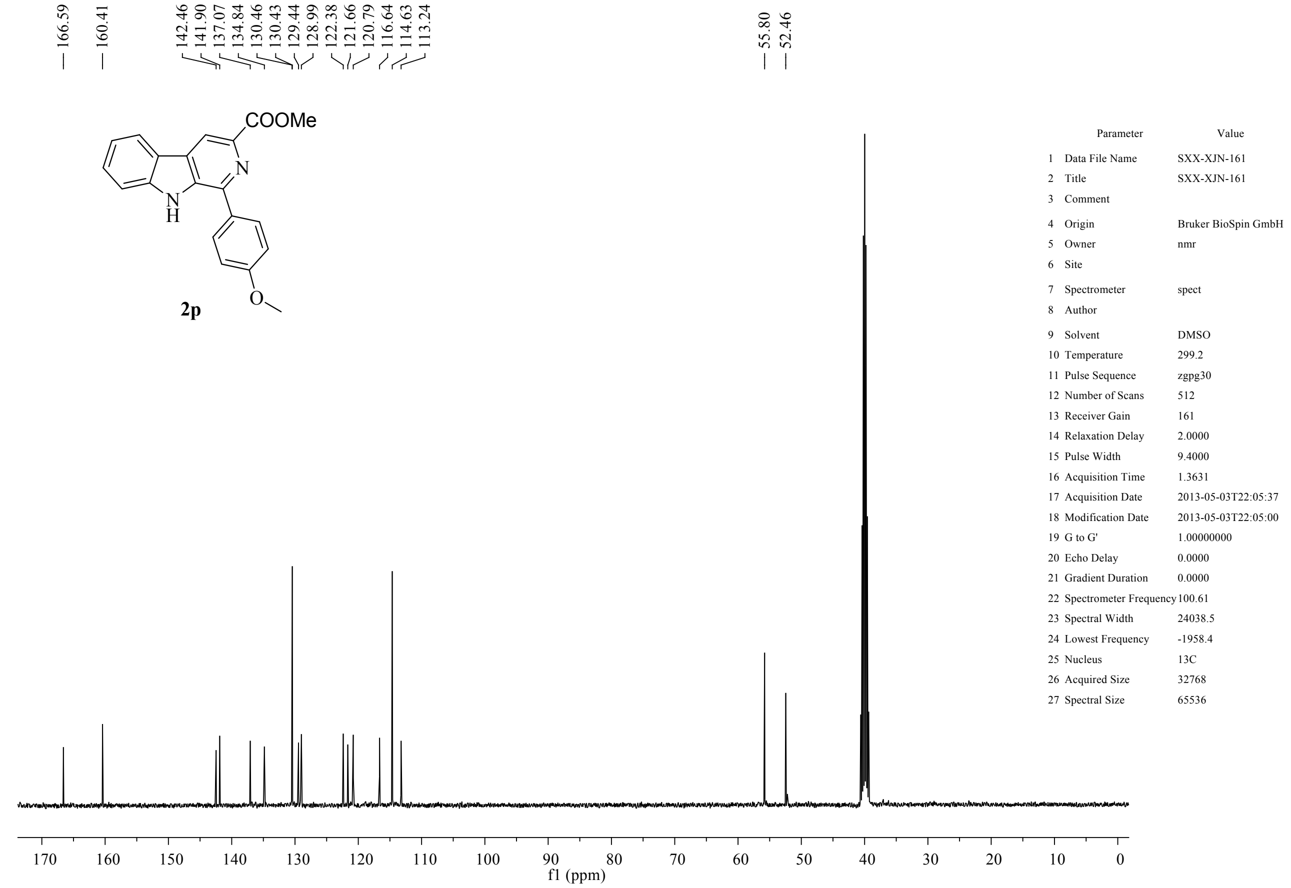

^{1}H NMR (DMSO-d_6, 400 MHz) spectrum of compound **2q**:

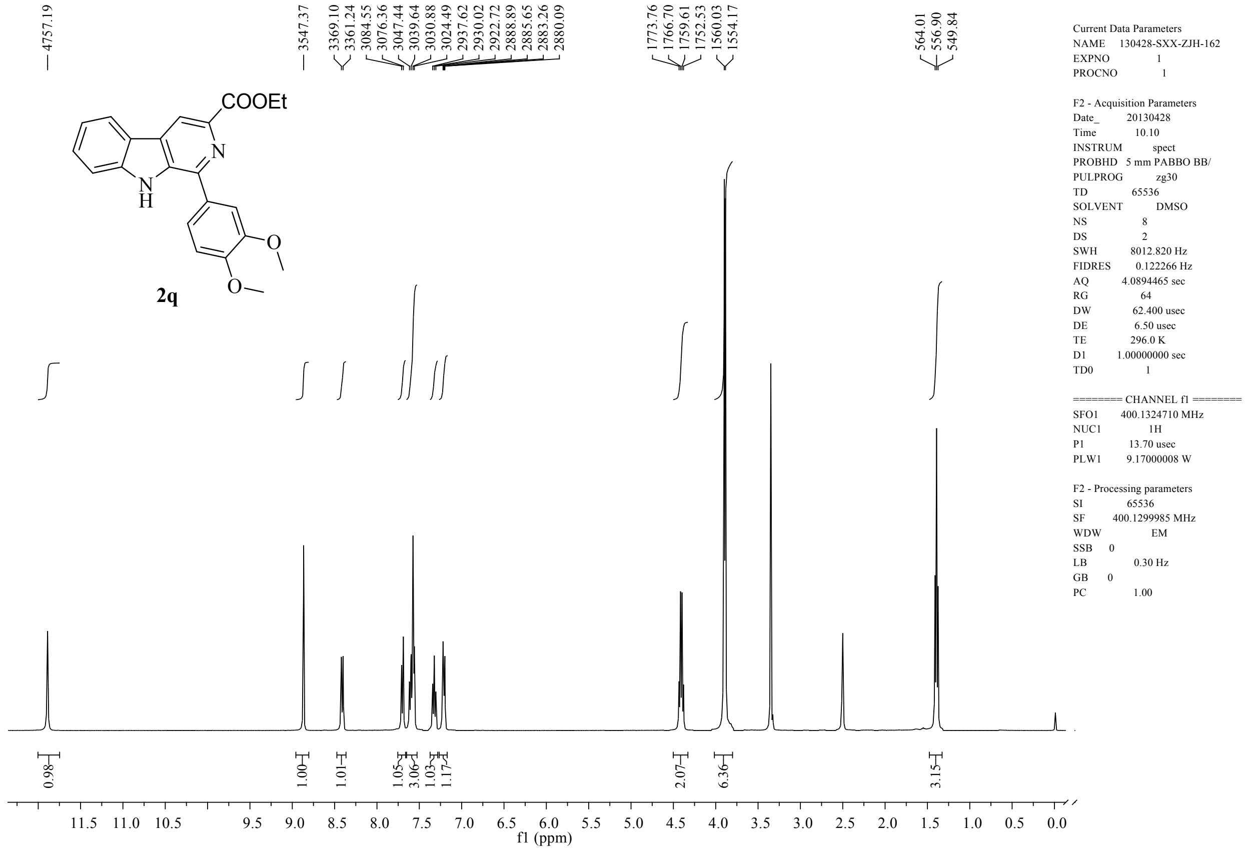

^{13}C NMR (DMSO-d_6, 100 MHz) spectrum of compound **2q**:

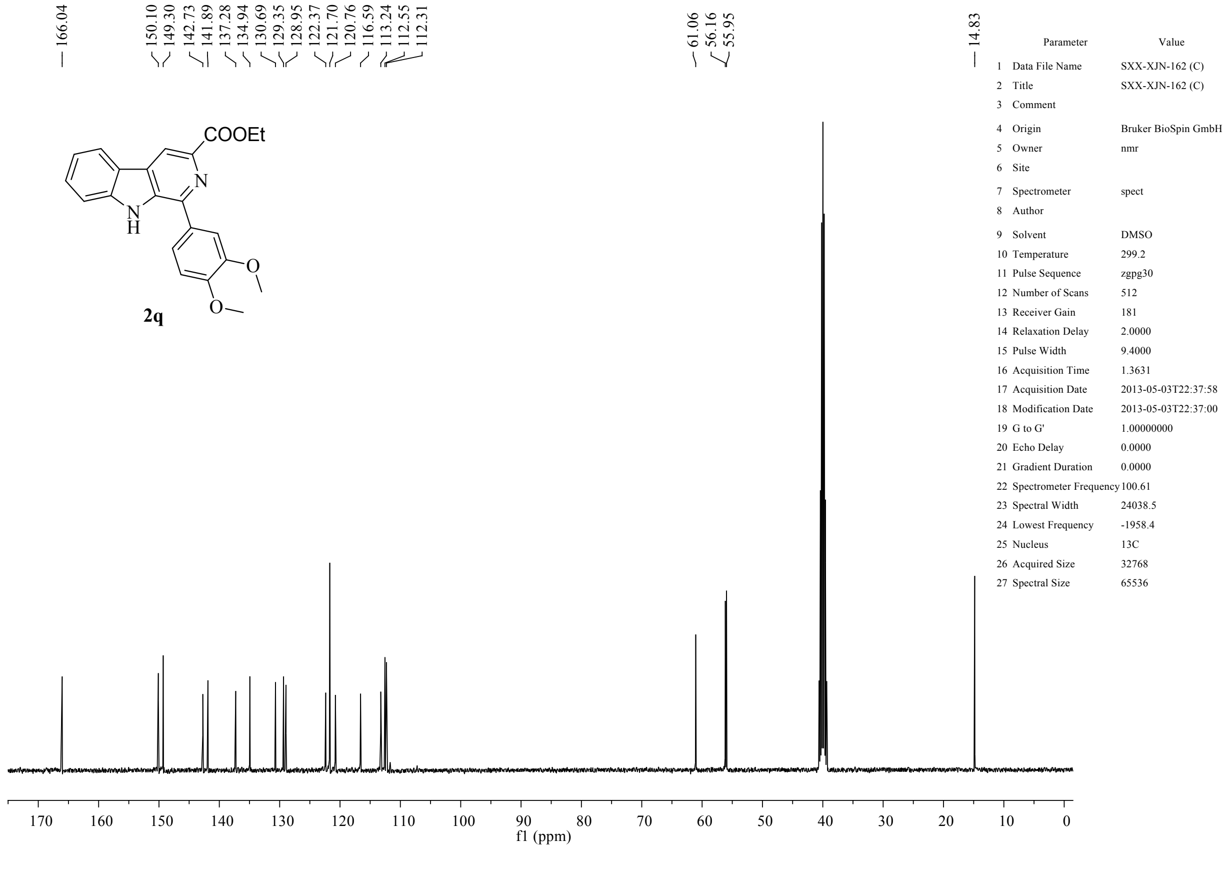

^{1}H NMR (DMSO-d_6, 400 MHz) spectrum of compound **2r**:

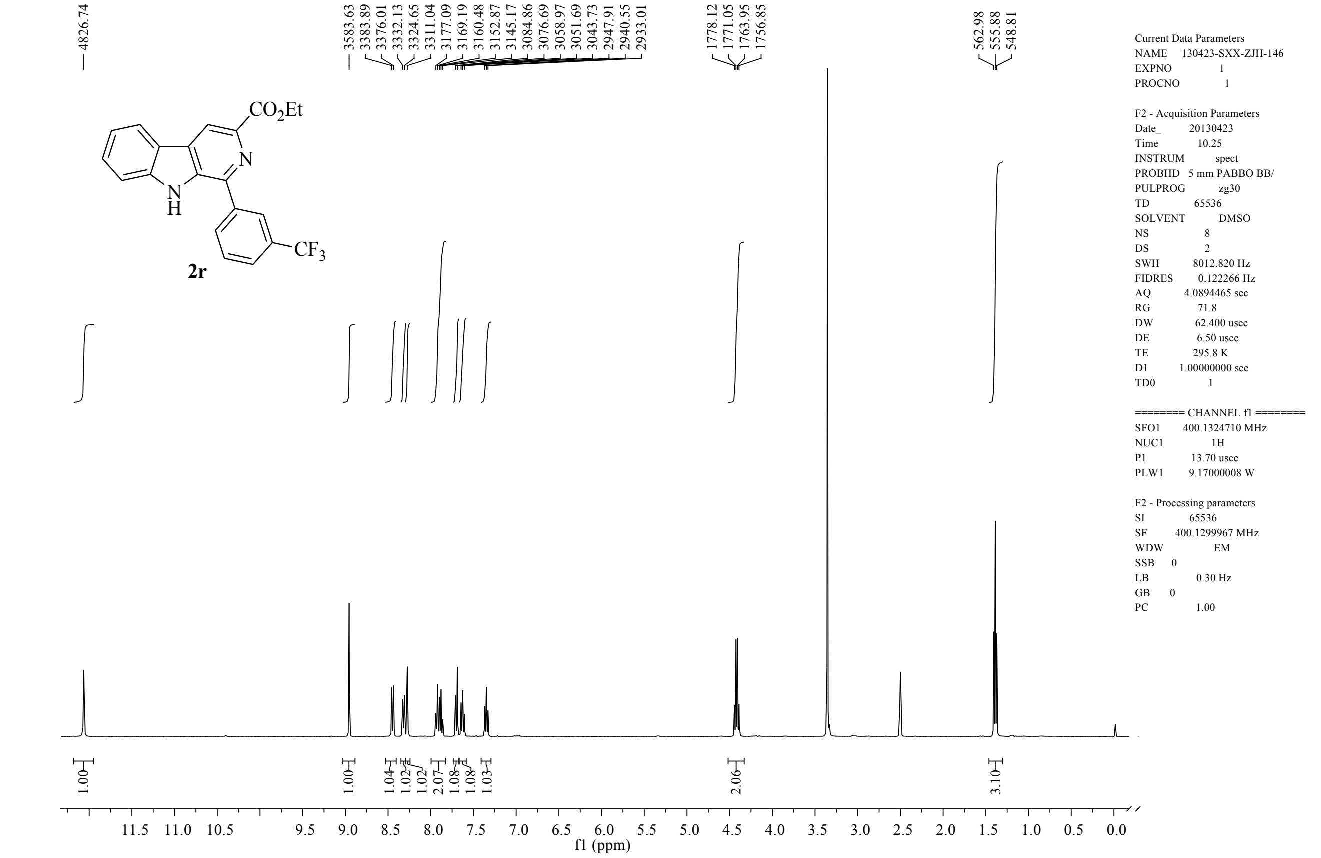

^{13}C NMR (DMSO-d_6, 100 MHz) spectrum of compound **2r**:

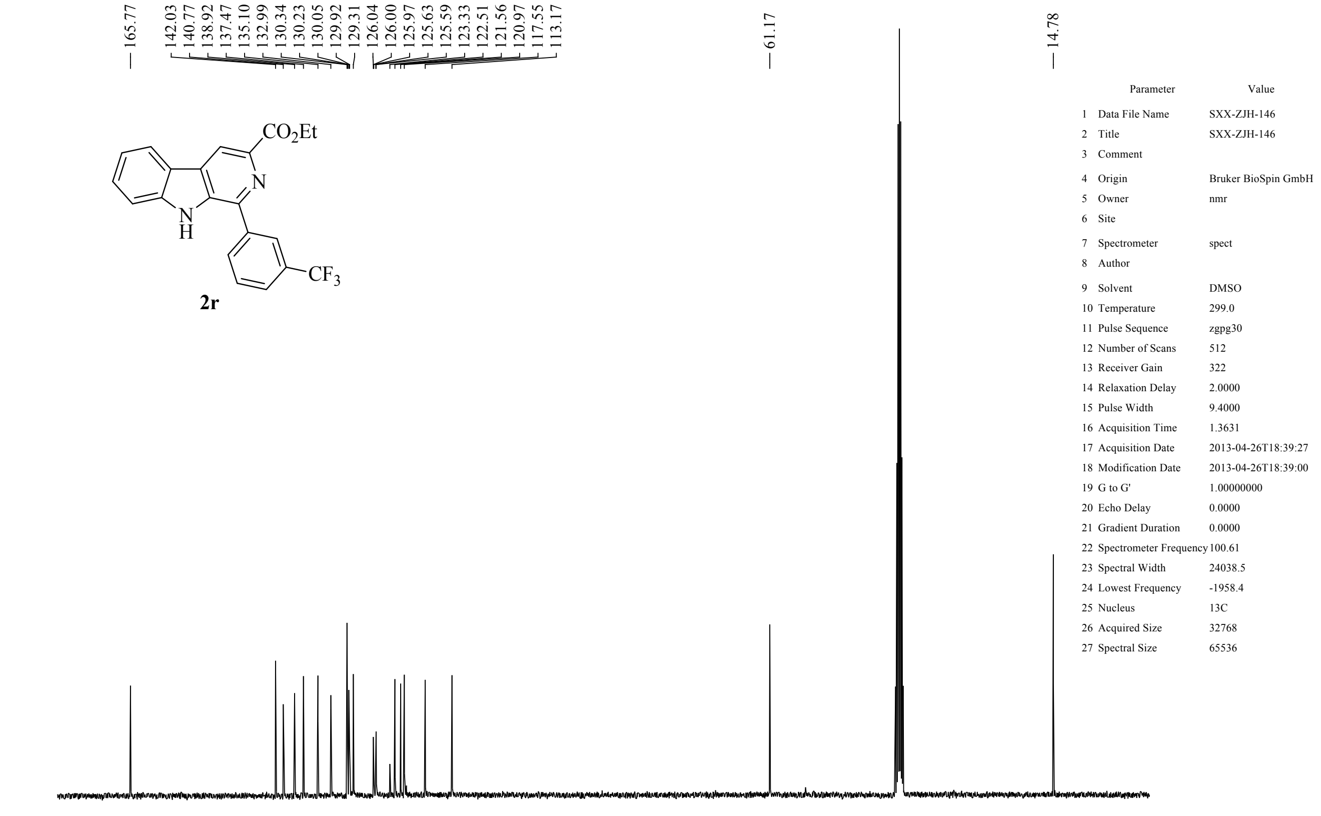

^{1}H NMR (DMSO-d_6, 400 MHz) spectrum of compound **2s**:

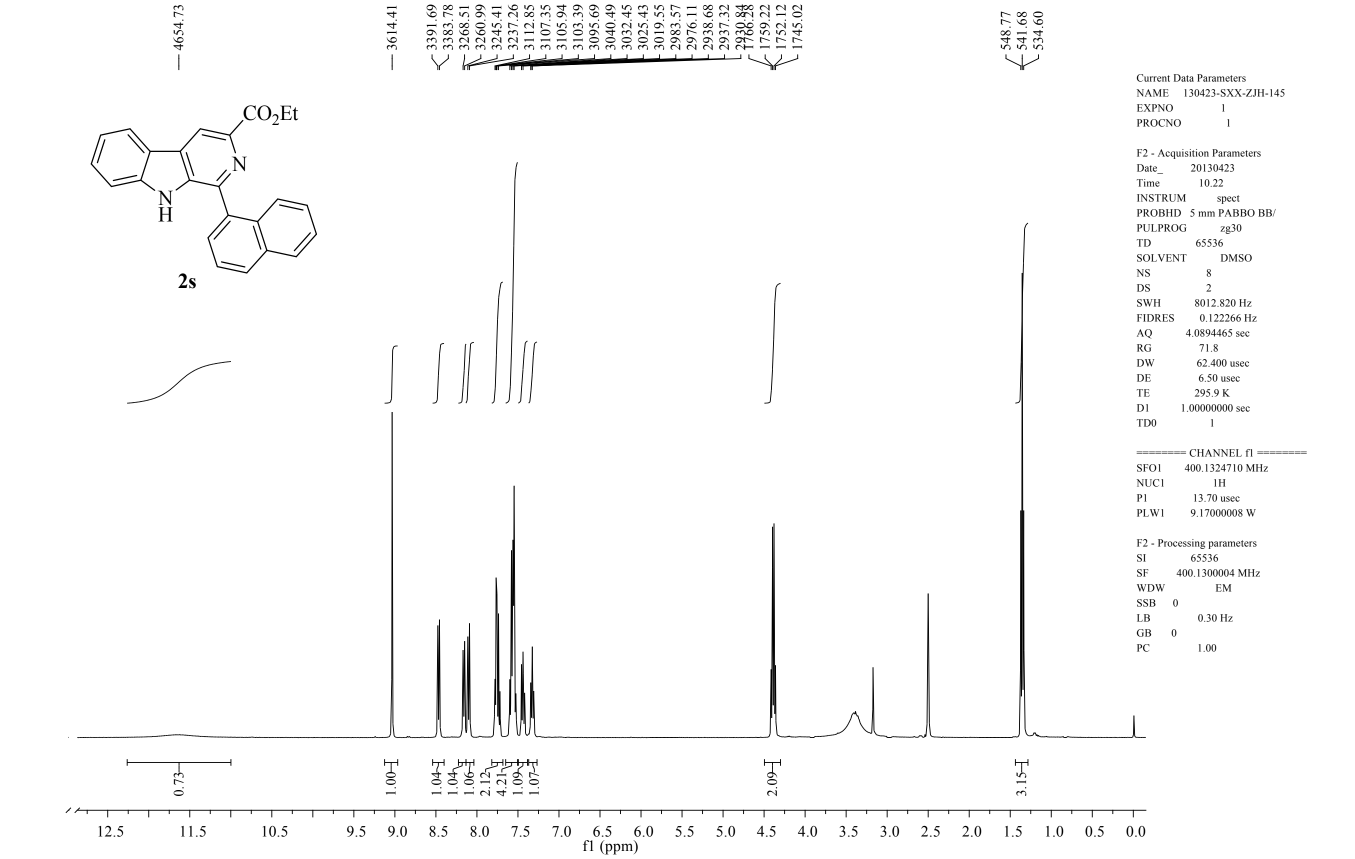

^{13}C NMR (DMSO-d_6, 100 MHz) spectrum of compound **2s**:

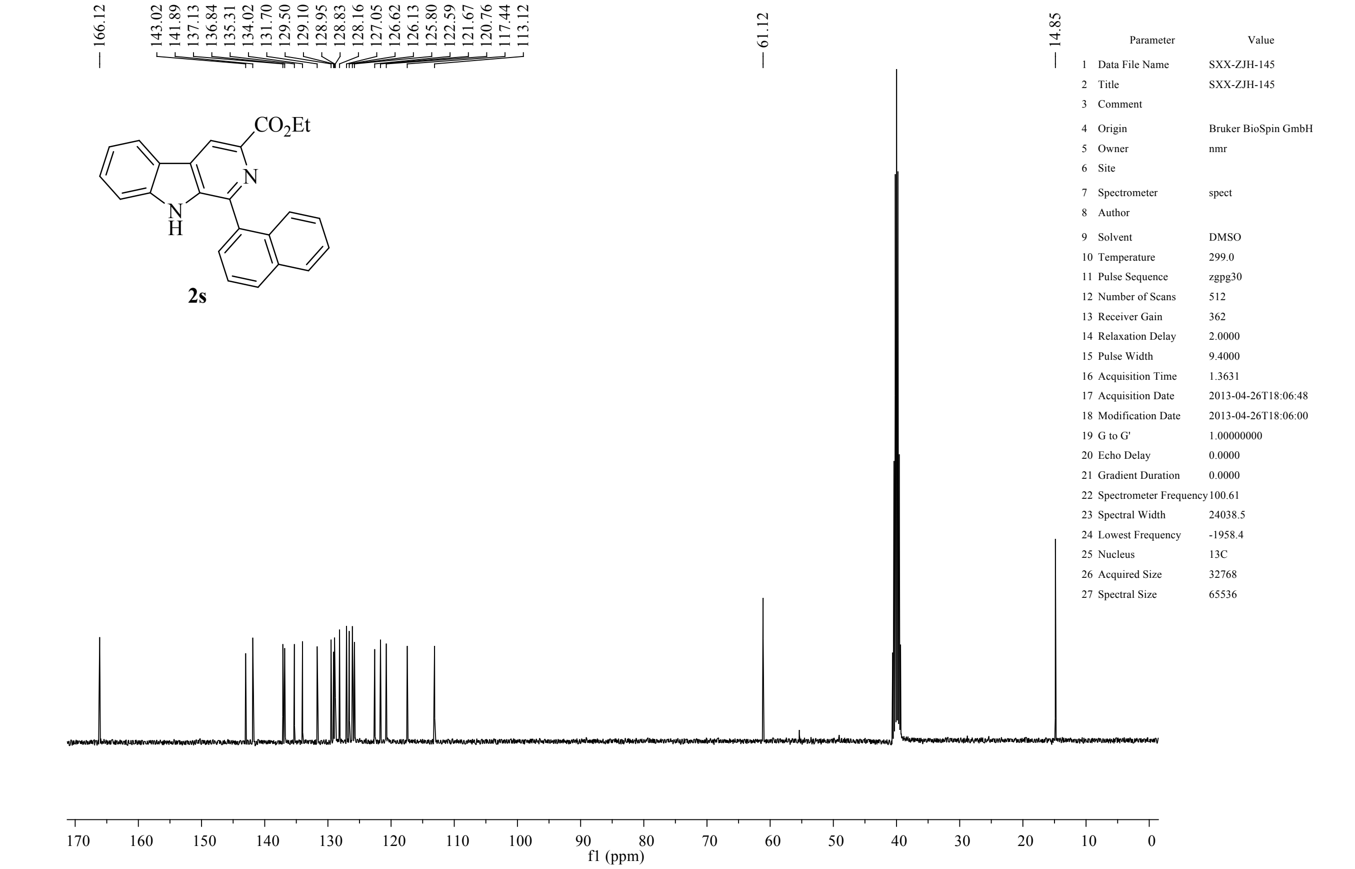

^{1}H NMR (DMSO-d_6, 400 MHz) spectrum of compound **2t**:

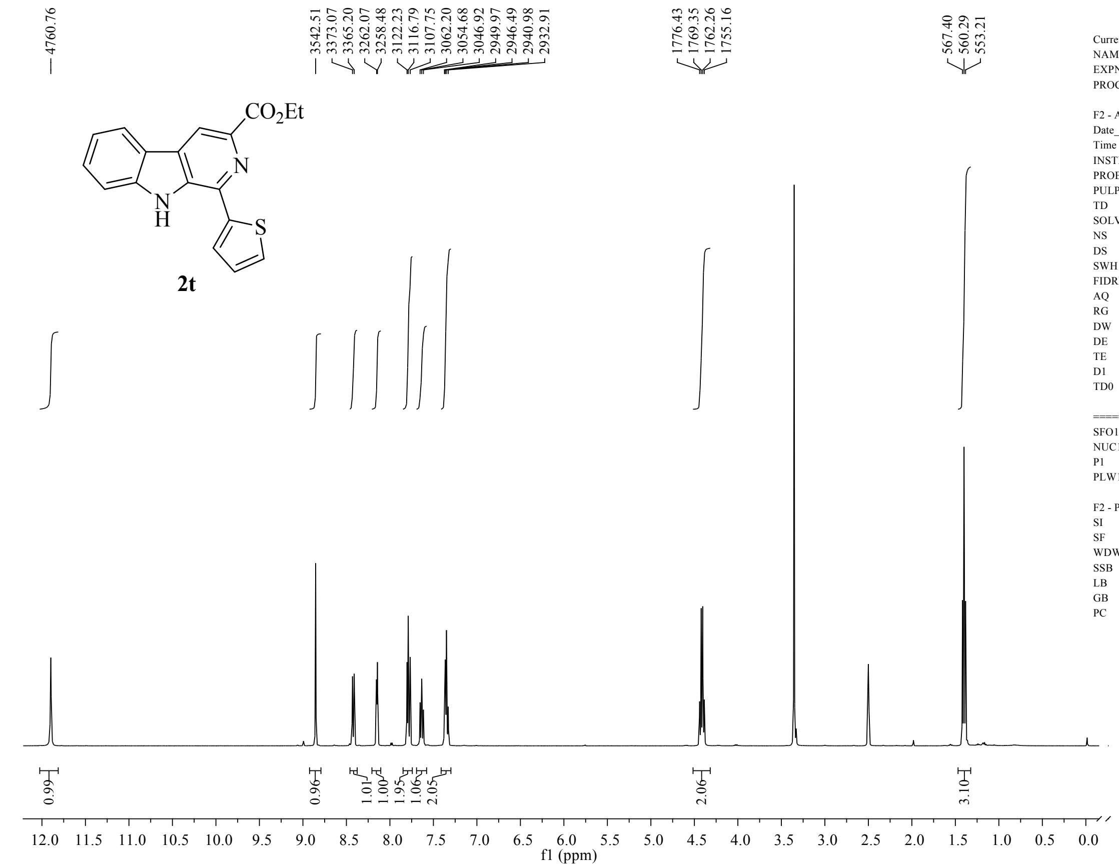

Current Data Parameters
NAME 130423-SXX-ZJH-143
EXPNO 1
PROCNO 1

F2 - Acquisition Parameters
Date_ 20130423
Time 10.29
INSTRUM spect
PROBHD 5 mm PABBO BB/
PULPROG zg30
TD 65536
SOLVENT DMSO
NS 16
DS 2
SWH 8012.820 Hz
FIDRES 0.122266 Hz
AQ 4.0894465 sec
RG 80.6
DW 62.400 usec
DE 6.50 usec
TE 295.8 K
D1 1.00000000 sec
TD0 1

======== CHANNEL f1 ========
SFO1 400.1324710 MHz
NUC1 1H
P1 13.70 usec
PLW1 9.17000008 W

F2 - Processing parameters
SI 65536
SF 400.1299971 MHz
WDW EM
SSB 0
LB 0.30 Hz
GB 0
PC 1.00

^{13}C NMR (DMSO-d_6, 100 MHz) spectrum of compound **2t**:

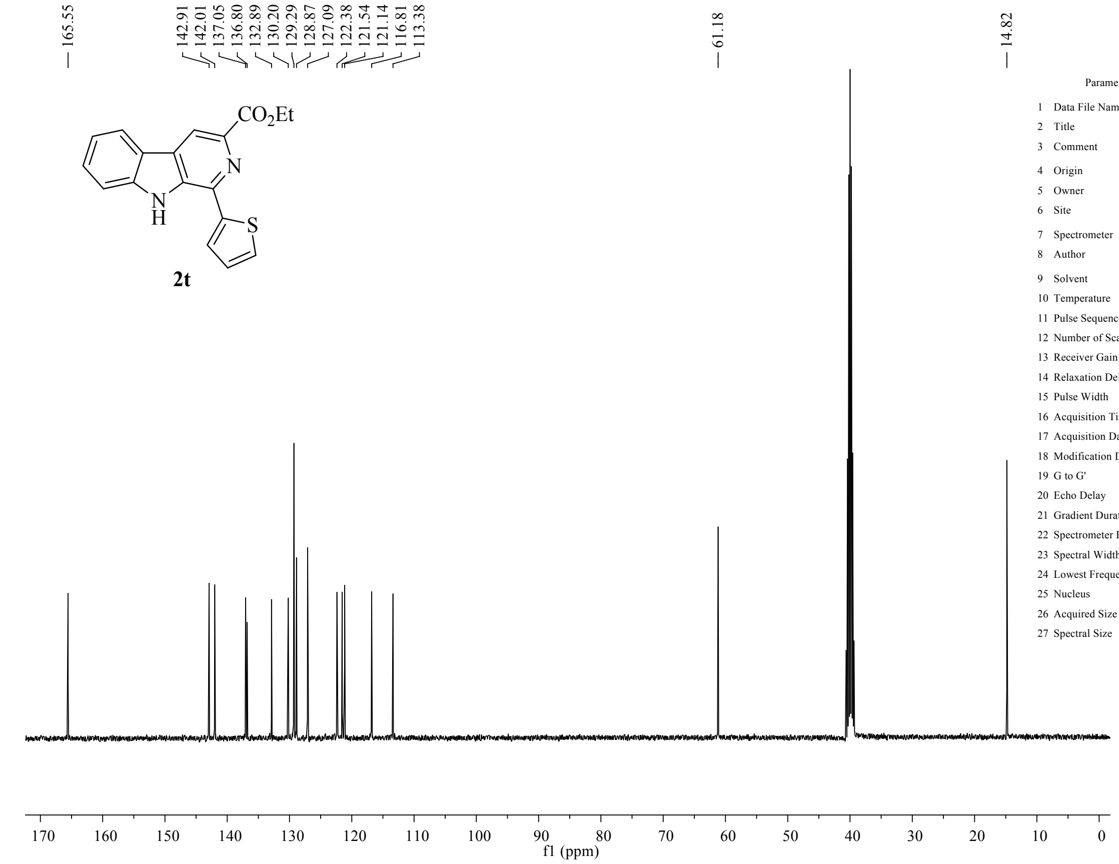

	Parameter	Value
1	Data File Name	SXX-ZJH-143
2	Title	SXX-ZJH-143
3	Comment	
4	Origin	Bruker BioSpin GmbH
5	Owner	nmr
6	Site	
7	Spectrometer	spect
8	Author	
9	Solvent	DMSO
10	Temperature	299.0
11	Pulse Sequence	zgpg30
12	Number of Scans	512
13	Receiver Gain	40
14	Relaxation Delay	2.0000
15	Pulse Width	9.4000
16	Acquisition Time	1.3631
17	Acquisition Date	2013-04-26T17:34:26
18	Modification Date	2013-04-26T17:34:00
19	G to G'	1.00000000
20	Echo Delay	0.0000
21	Gradient Duration	0.0000
22	Spectrometer Frequency	100.61
23	Spectral Width	24038.5
24	Lowest Frequency	-1958.4
25	Nucleus	13C
26	Acquired Size	32768
27	Spectral Size	65536

^{1}H NMR (DMSO-d_6, 400 MHz) spectrum of compound **2u**:

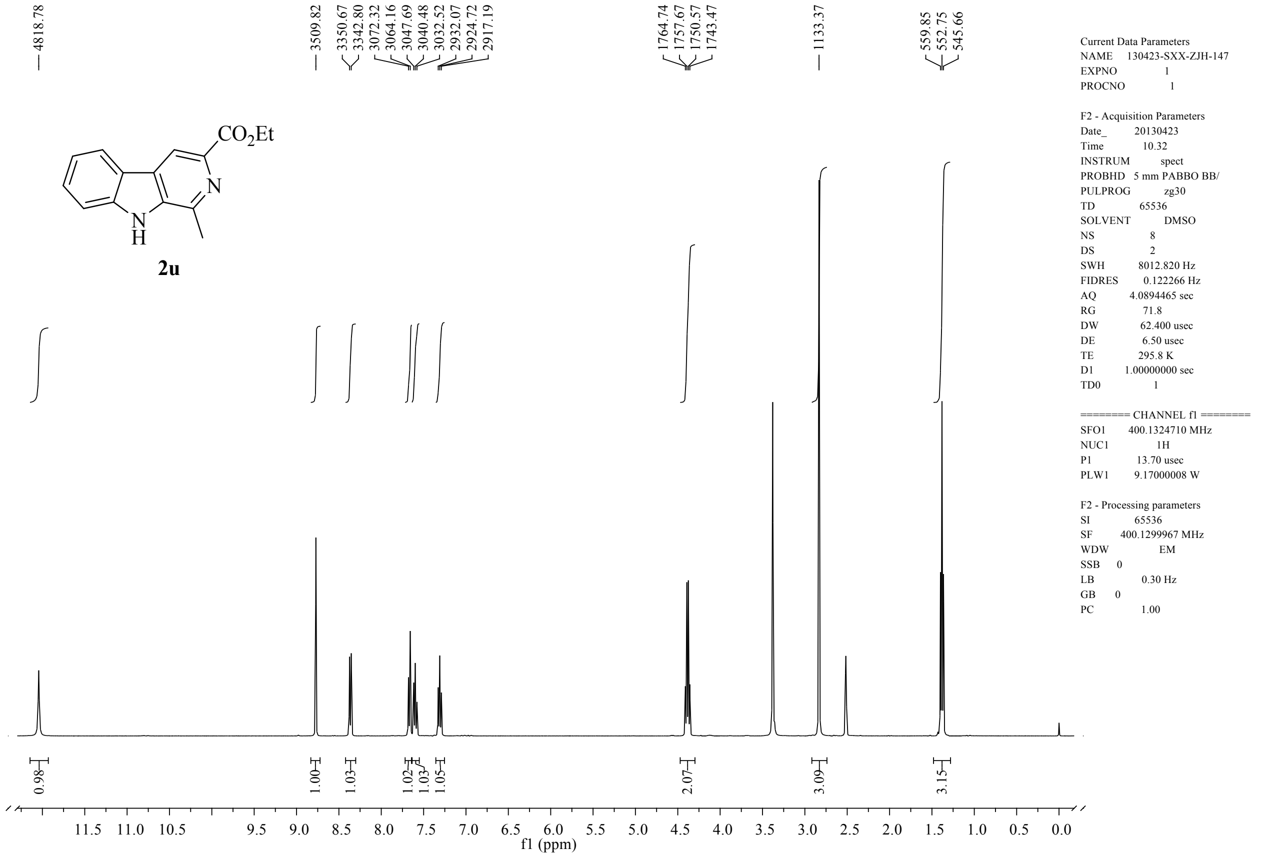

^{13}C NMR (DMSO-d_6, 100 MHz) spectrum of compound **2u**:

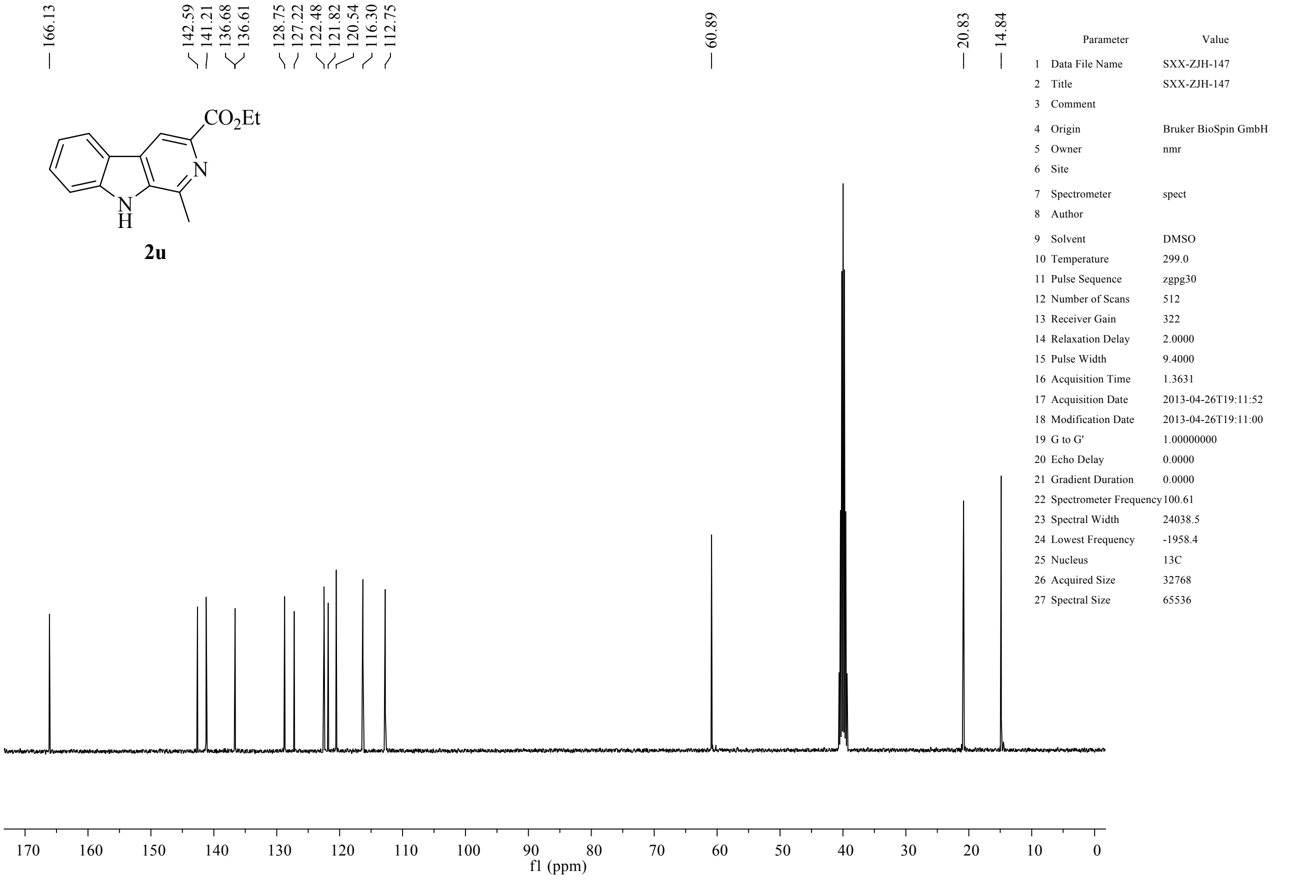

^{1}H NMR (DMSO-d_6, 400 MHz) spectrum of compound **2v**:

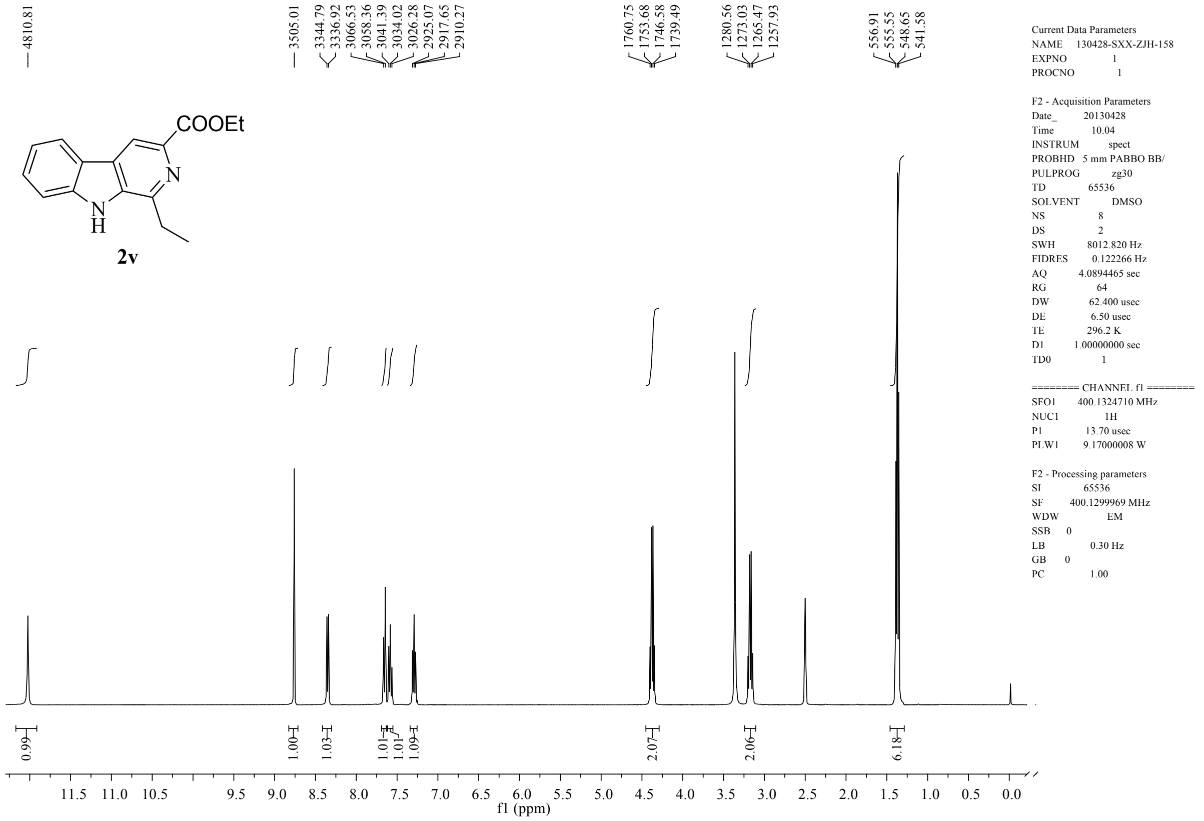

^{13}C NMR (DMSO-d_6, 100 MHz) spectrum of compound **2v**:

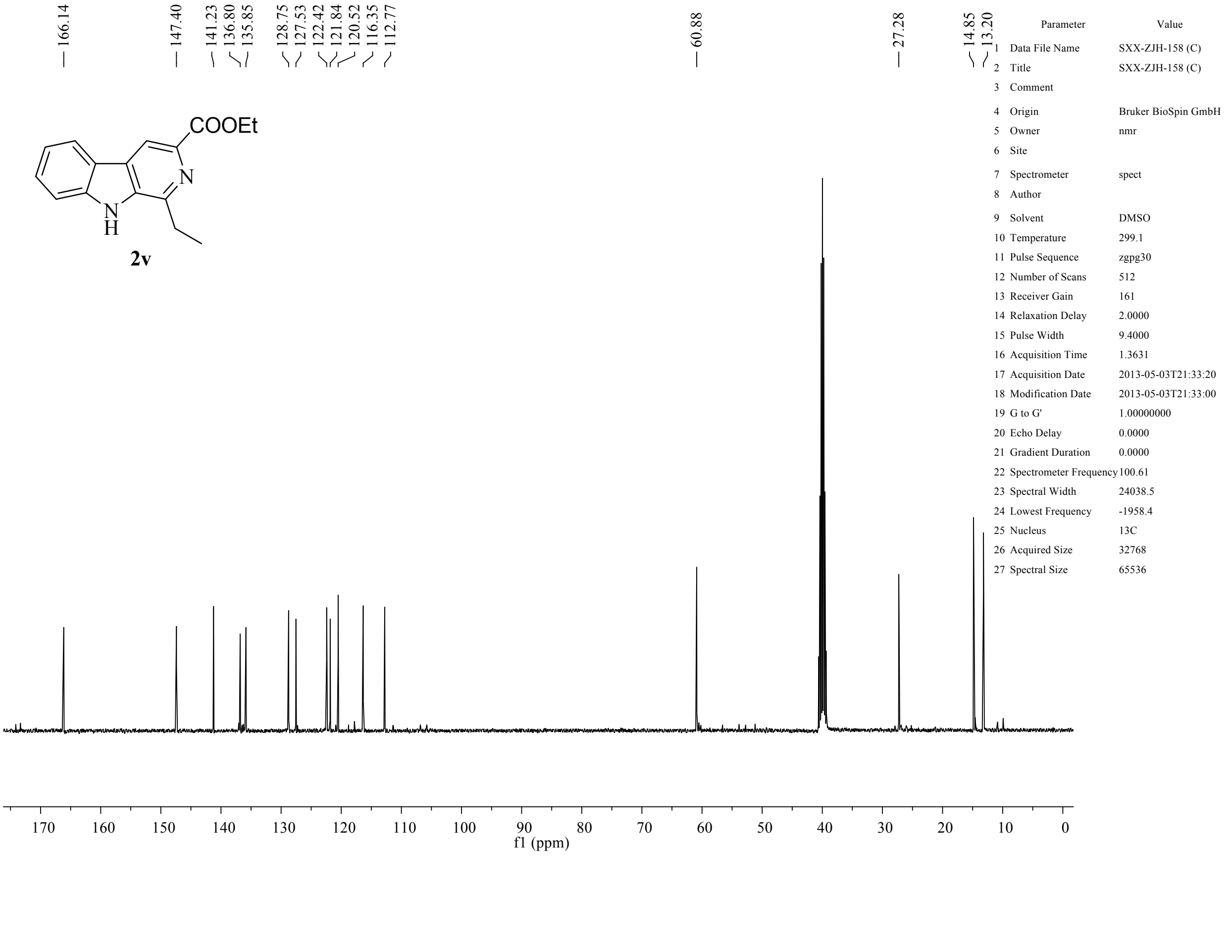

^{1}H NMR (DMSO-d_6, 400 MHz) spectrum of compound **2w**:

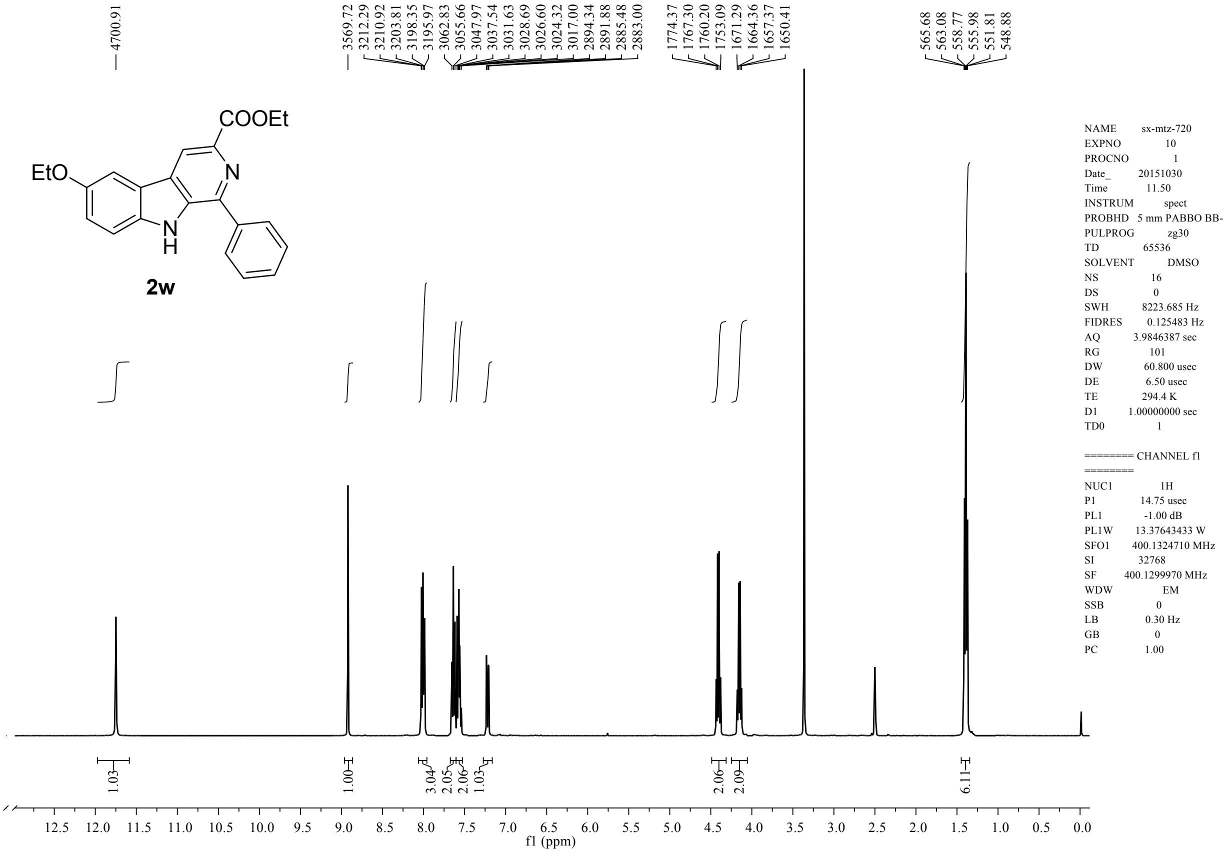

^{13}C NMR (DMSO-d_6, 100 MHz) spectrum of compound **2w**:

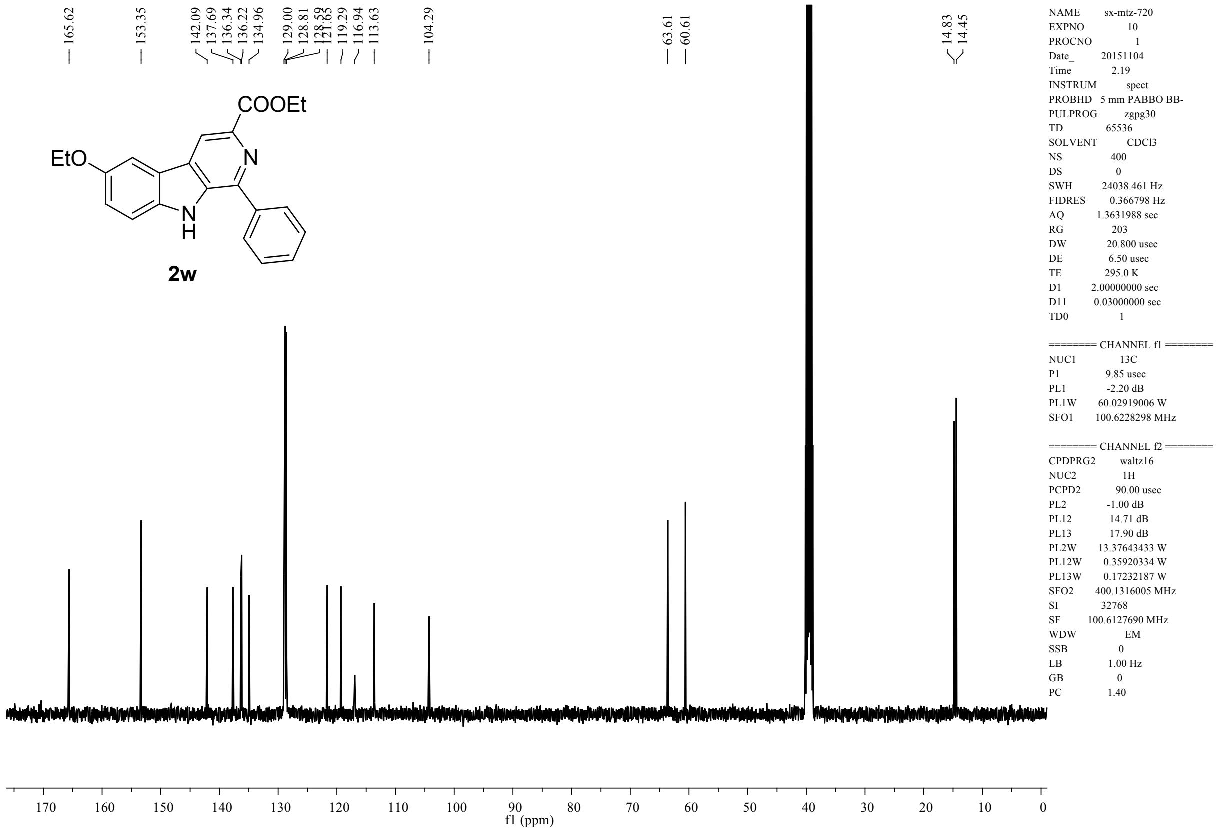

^1H NMR (DMSO-d_6, 400 MHz) spectrum of compound **2x**:

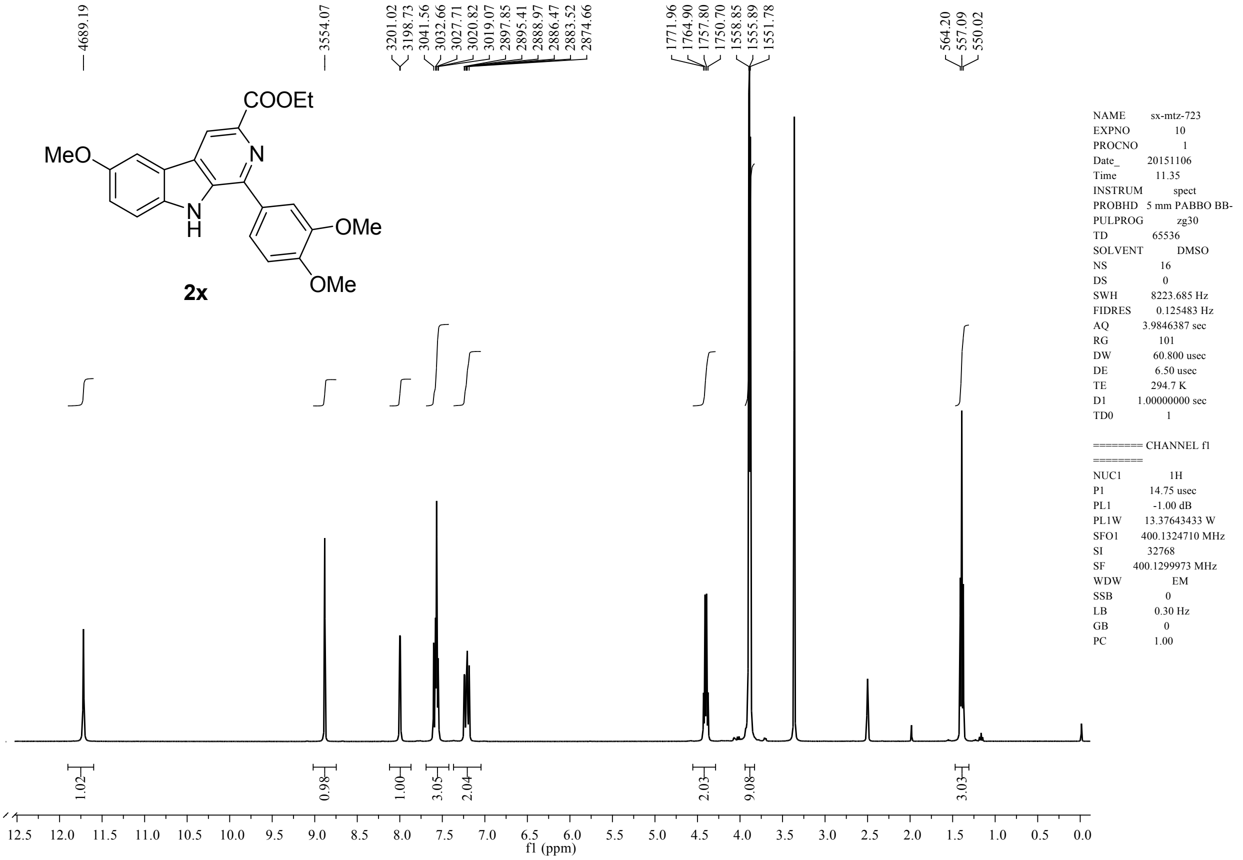

^{13}C NMR (DMSO-d_6, 100 MHz) spectrum of compound **2x**:

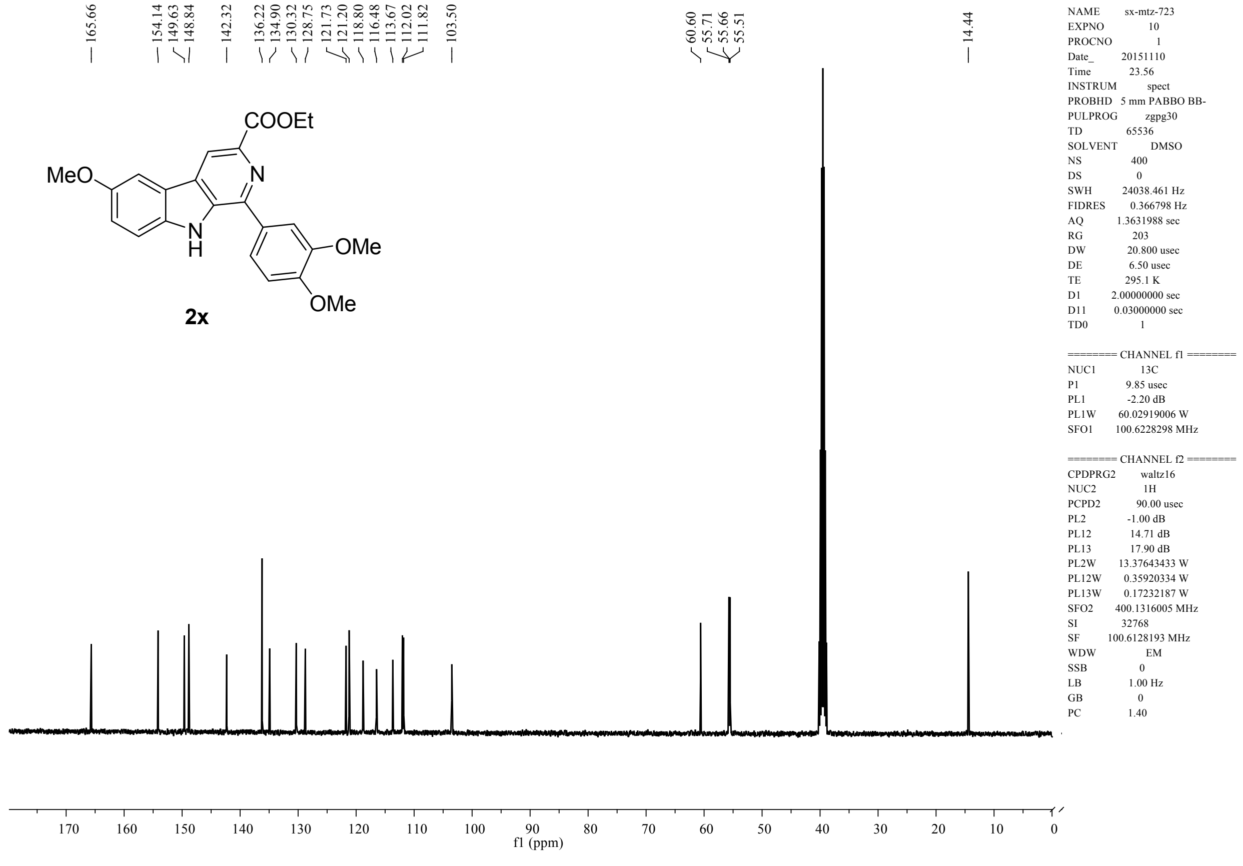

^{1}H NMR (DMSO-d_6, 400 MHz) spectrum of perlolyrine **3**:

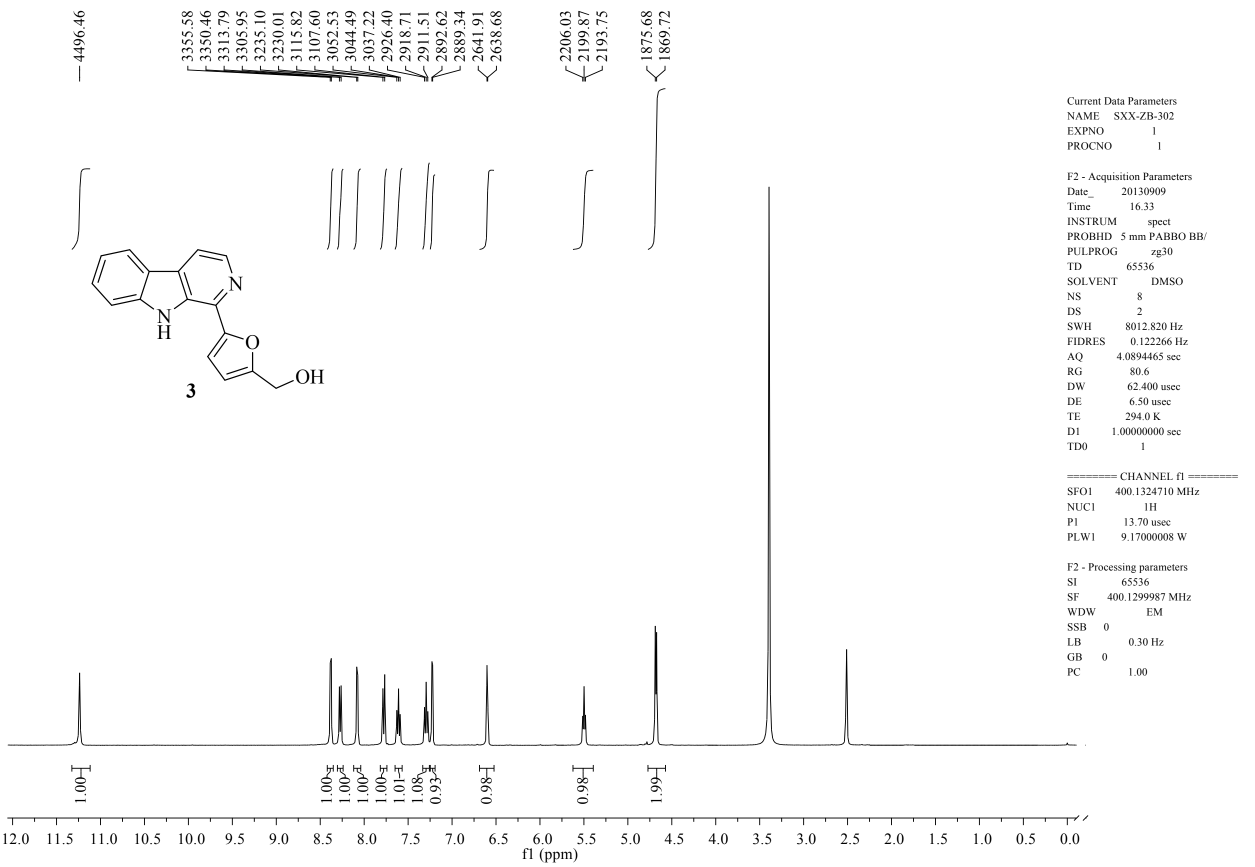

^{13}C NMR (DMSO-d_6, 100 MHz) spectrum of perlolyrine **3**:

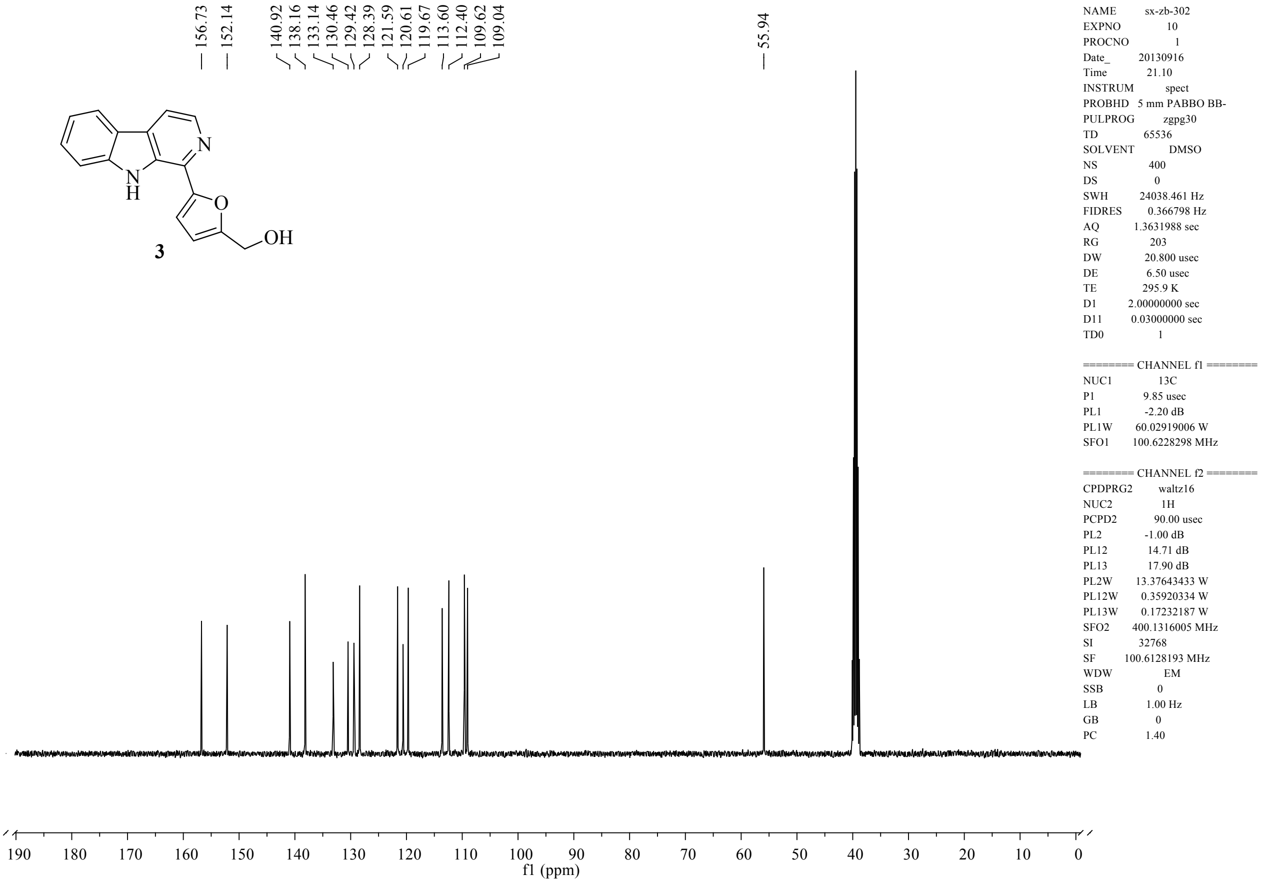

^{1}H NMR (DMSO-d_6, 400 MHz) spectrum of flazin **4**:

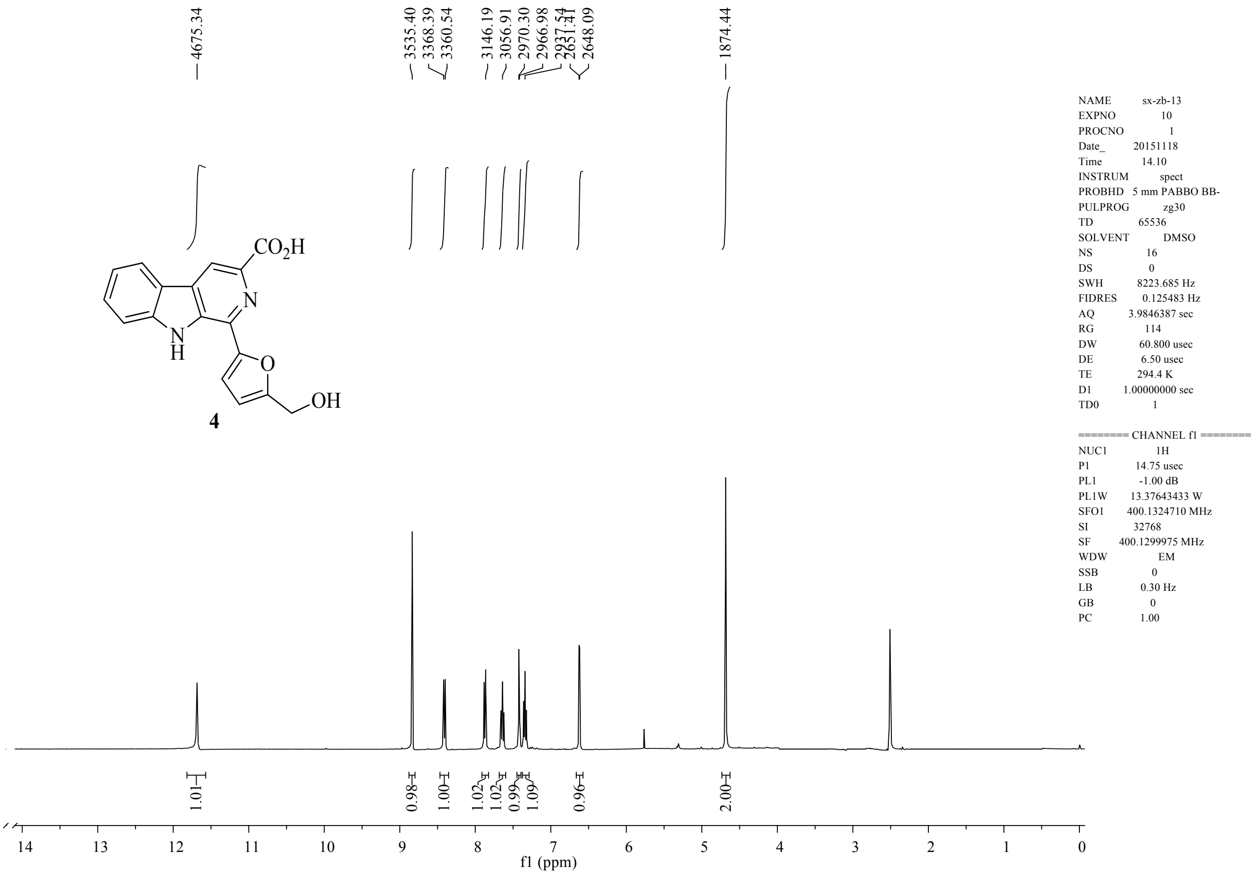

^{13}C NMR (DMSO-d_6, 100 MHz) spectrum of flazin **4**:

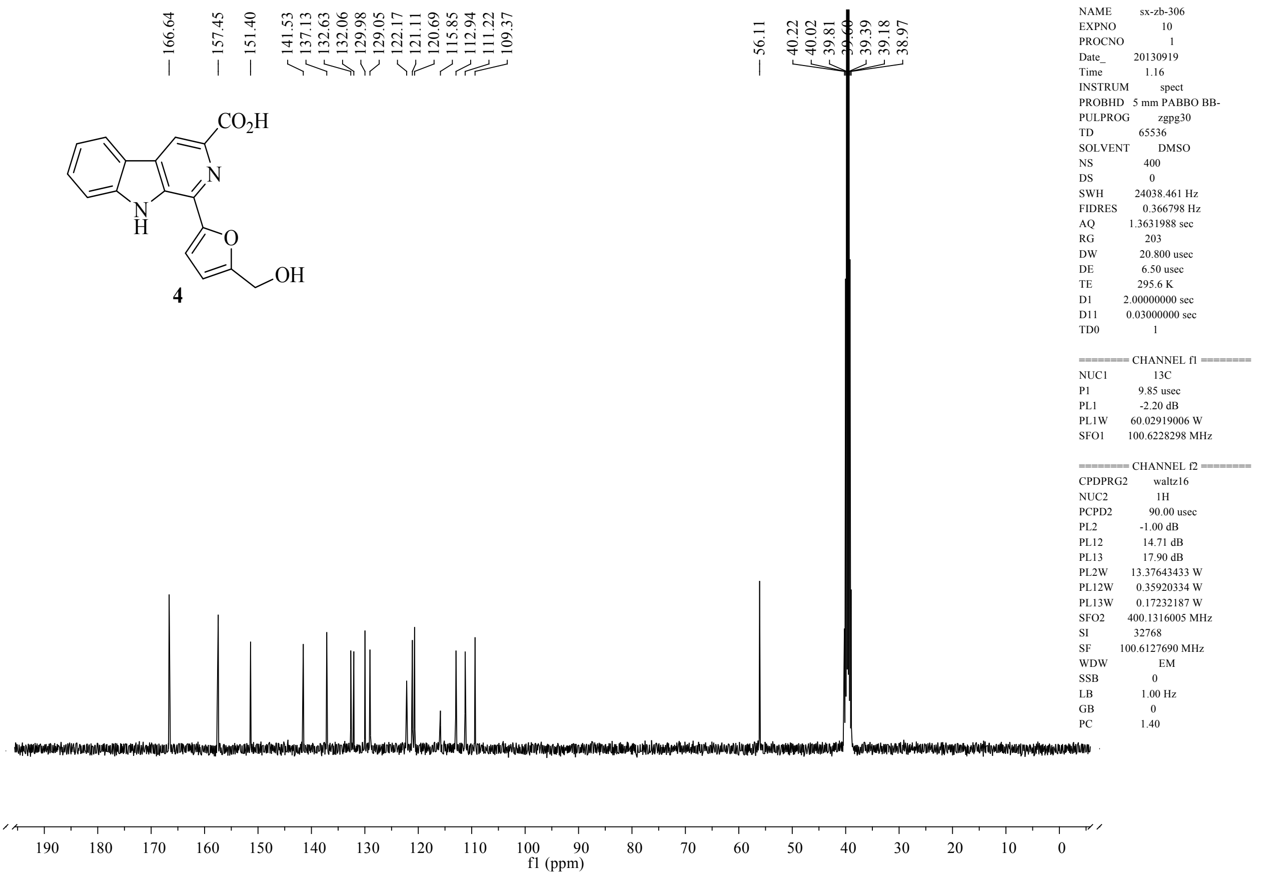

^{1}H NMR ($CDCl_3$, 400 MHz) spectrum of compound **5**:

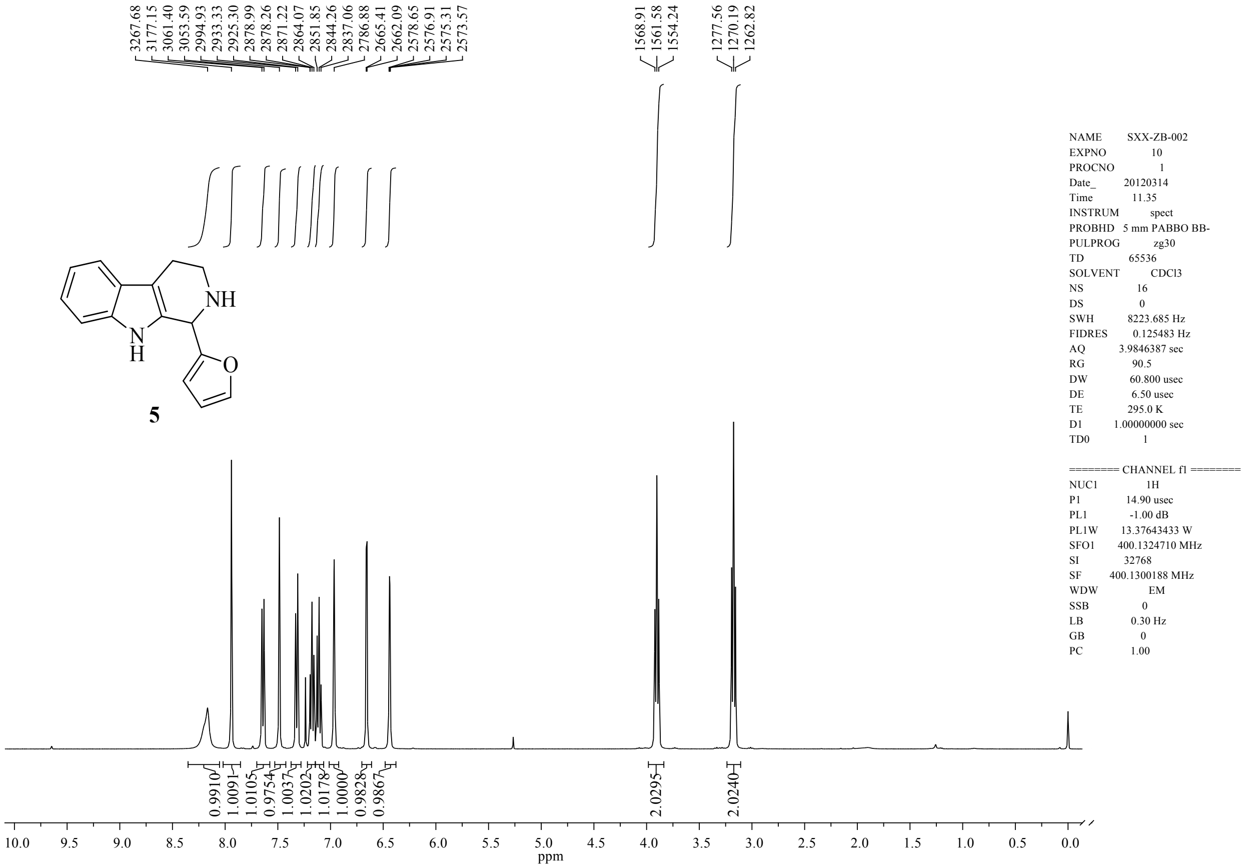

^{13}C NMR ($CDCl_3$, 100 MHz) spectrum of compound **9a**:

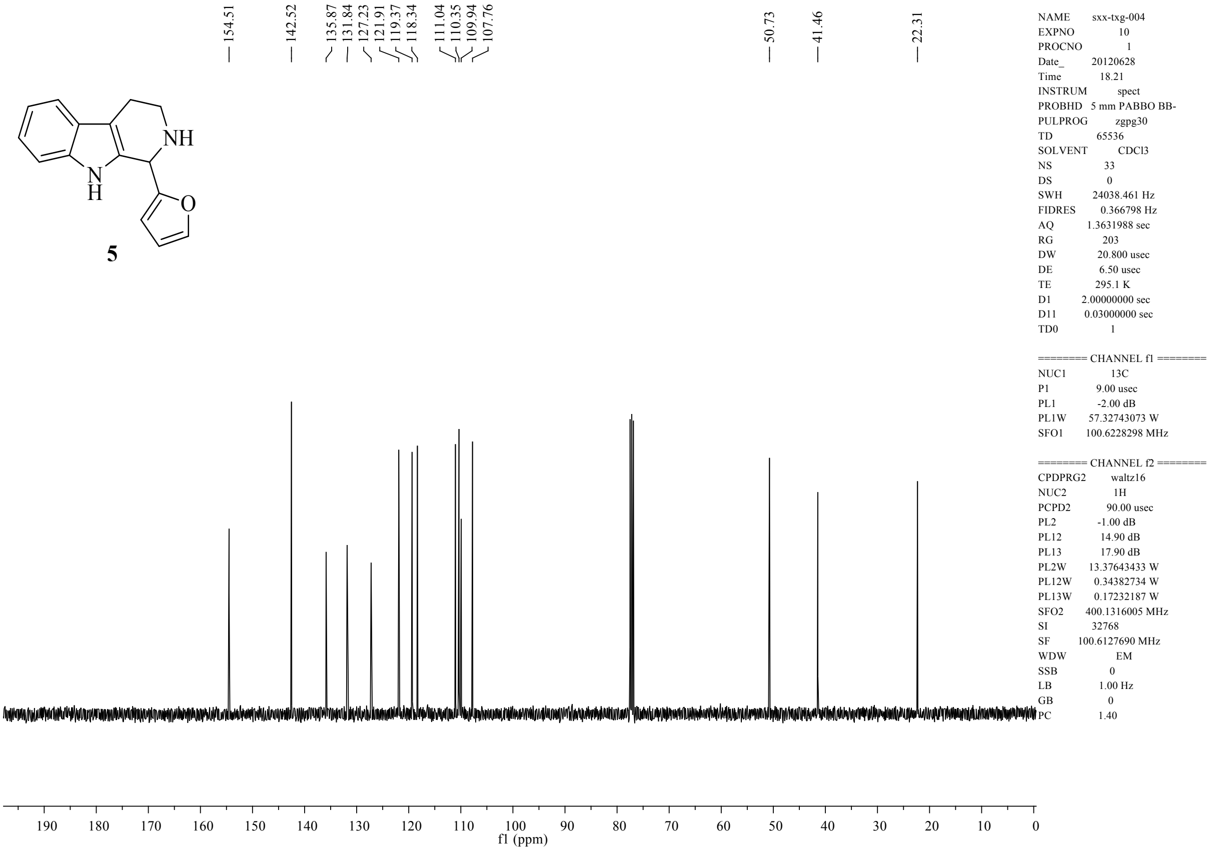

^{1}H NMR ($CDCl_3$, 400 MHz) spectrum of compound ***cis*-6**:

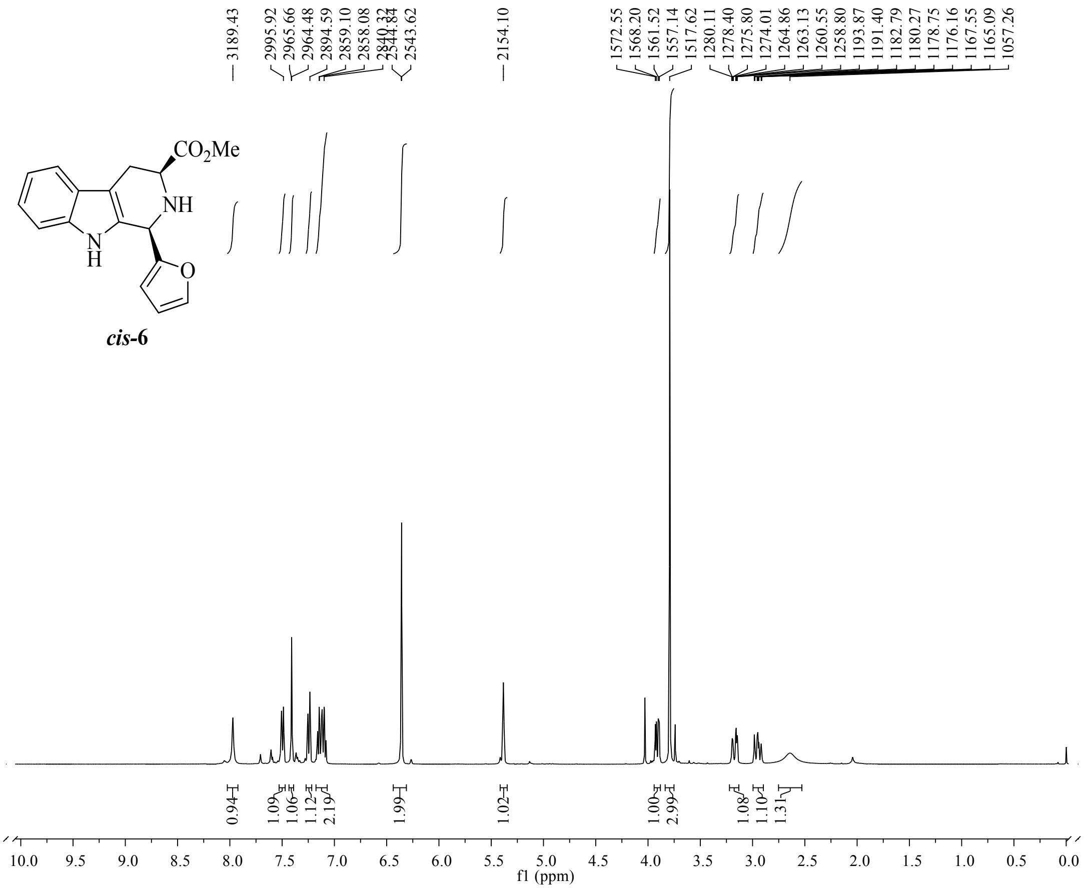

NAME sx-zb-041
EXPNO 10
PROCNO 1
Date_ 20151120
Time 7.05
INSTRUM spect
PROBHD 5 mm PABBO BB-
PULPROG zg30
TD 65536
SOLVENT CDCl3
NS 16
DS 0
SWH 8223.685 Hz
FIDRES 0.125483 Hz
AQ 3.9846387 sec
RG 71.8
DW 60.800 usec
DE 6.50 usec
TE 294.7 K
D1 1.00000000 sec
TD0 1

======== CHANNEL f1 ========
NUC1 1H
P1 14.75 usec
PL1 -1.00 dB
PL1W 13.37643433 W
SFO1 400.1324710 MHz
SI 32768
SF 400.1300178 MHz
WDW EM
SSB 0
LB 0.30 Hz
GB 0
PC 1.00

^{13}C NMR ($CDCl_3$, 100 MHz) spectrum of compound ***cis*-6**:

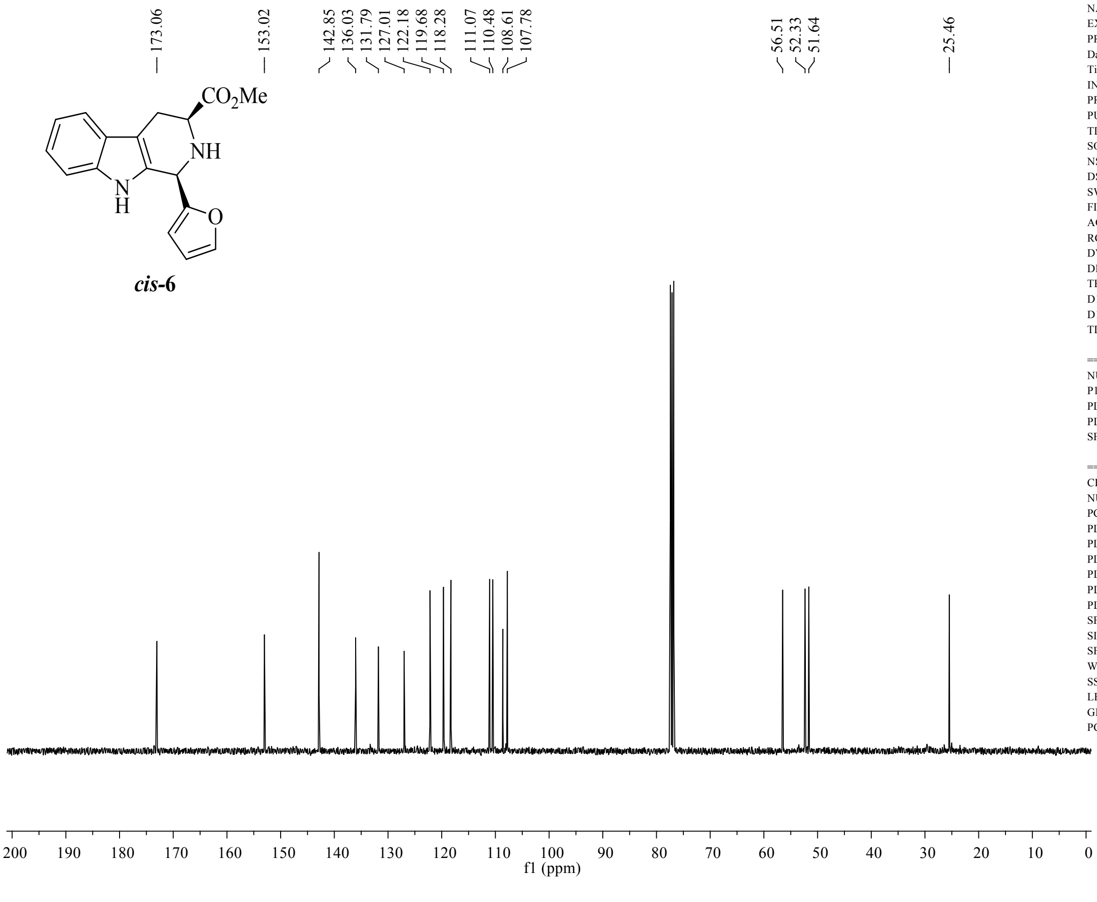

NAME sx-zb-041
EXPNO 11
PROCNO 1
Date_ 20151120
Time 7.30
INSTRUM spect
PROBHD 5 mm PABBO BB-
PULPROG zgpg30
TD 65536
SOLVENT CDCl3
NS 400
DS 0
SWH 24038.461 Hz
FIDRES 0.366798 Hz
AQ 1.3631988 sec
RG 203
DW 20.800 usec
DE 6.50 usec
TE 295.5 K
D1 2.00000000 sec
D11 0.03000000 sec
TD0 1

======== CHANNEL f1 ========
NUC1 13C
P1 9.85 usec
PL1 -2.20 dB
PL1W 60.02919006 W
SFO1 100.6228298 MHz

======== CHANNEL f2 ========
CPDPRG2 waltz16
NUC2 1H
PCPD2 90.00 usec
PL2 -1.00 dB
PL12 14.71 dB
PL13 17.90 dB
PL2W 13.37643433 W
PL12W 0.35920334 W
PL13W 0.17232187 W
SFO2 400.1316005 MHz
SI 32768
SF 100.6127690 MHz
WDW EM
SSB 0
LB 1.00 Hz
GB 0
PC 1.40

^{1}H NMR ($CDCl_3$, 400 MHz) spectrum of compound ***trans*-6**:

***trans*-6**

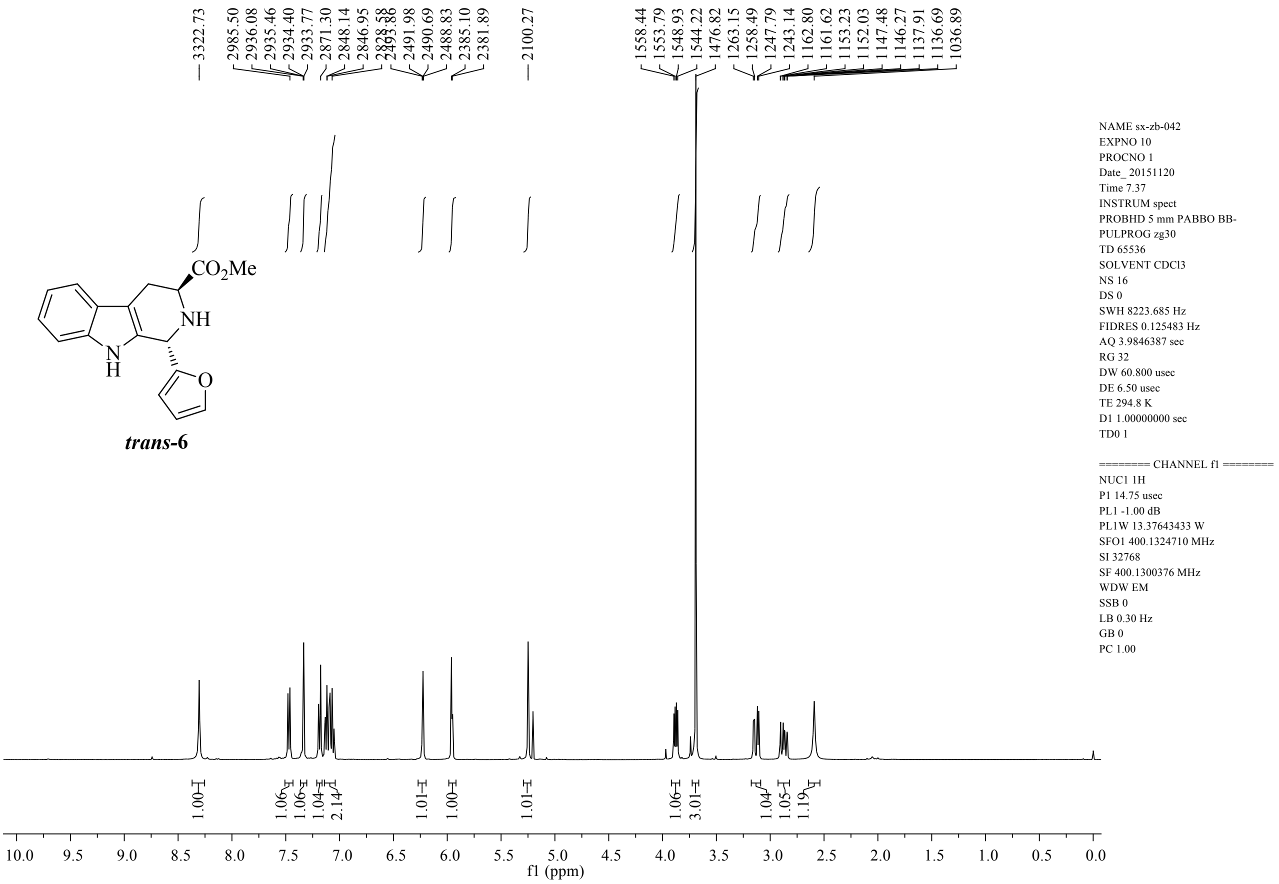

^{13}C NMR ($CDCl_3$, 100 MHz) spectrum of compound ***trans*-6**:

***trans*-6**

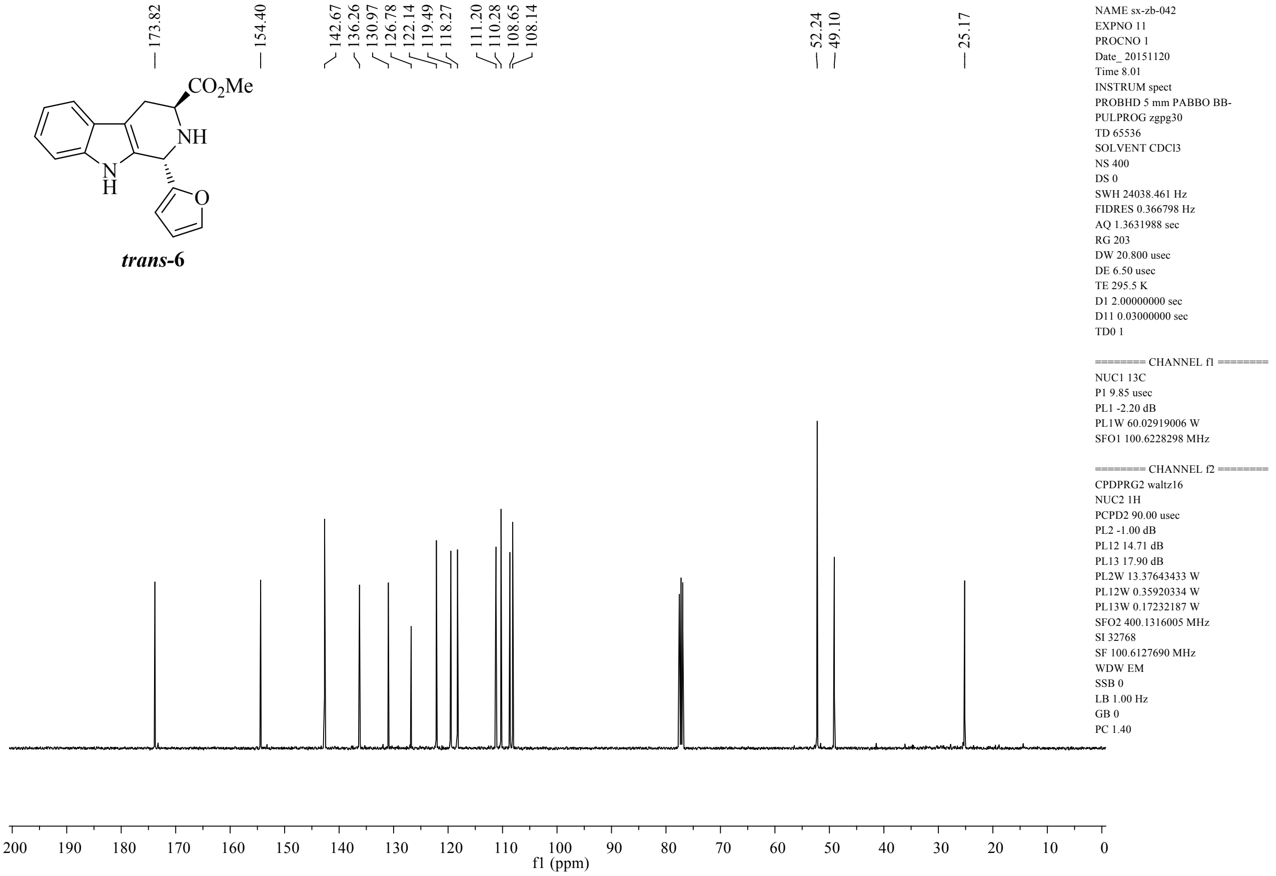

^{1}H NMR (DMSO-d_6, 400 MHz) spectrum of compound **7**:

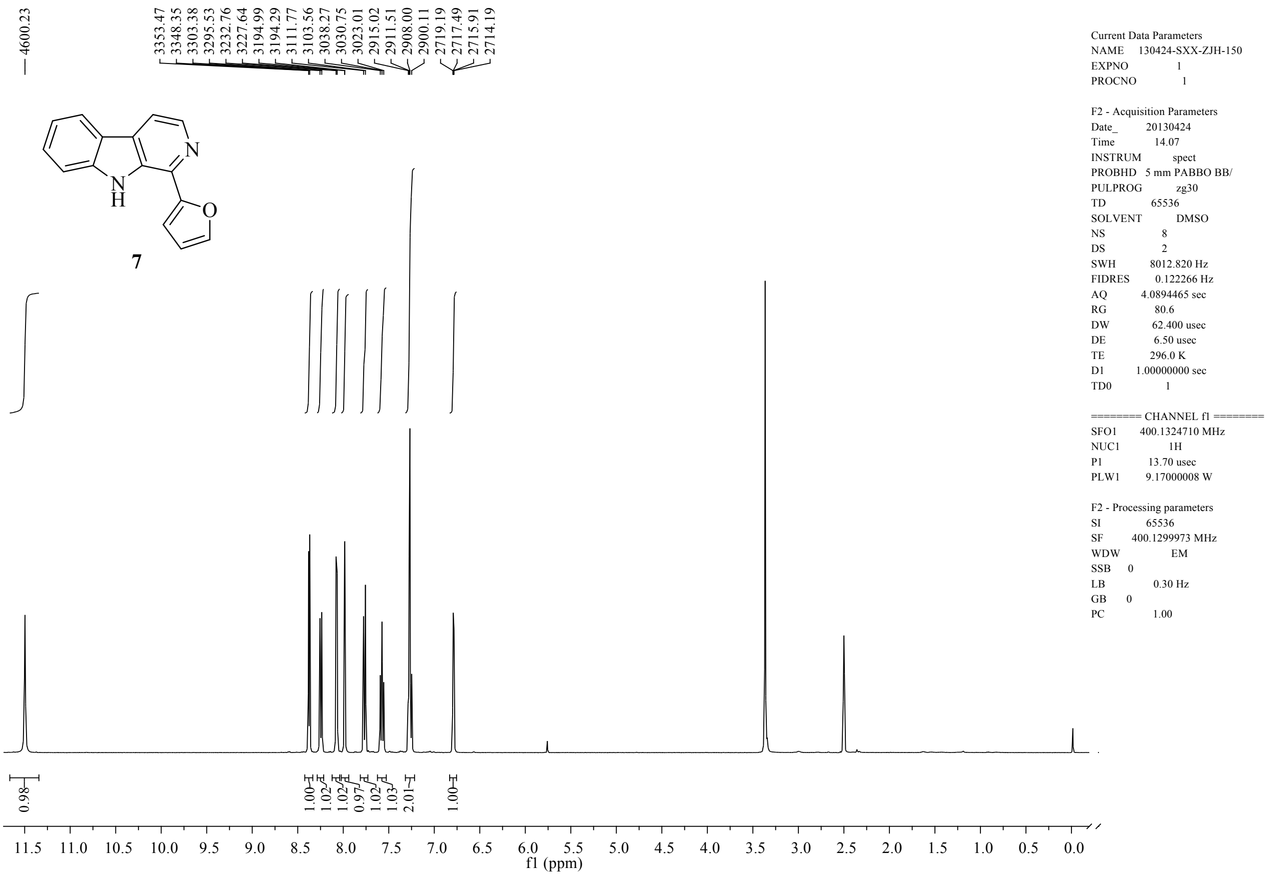

^{13}C NMR ($CDCl_3$, 100 MHz) spectrum of compound **7**:

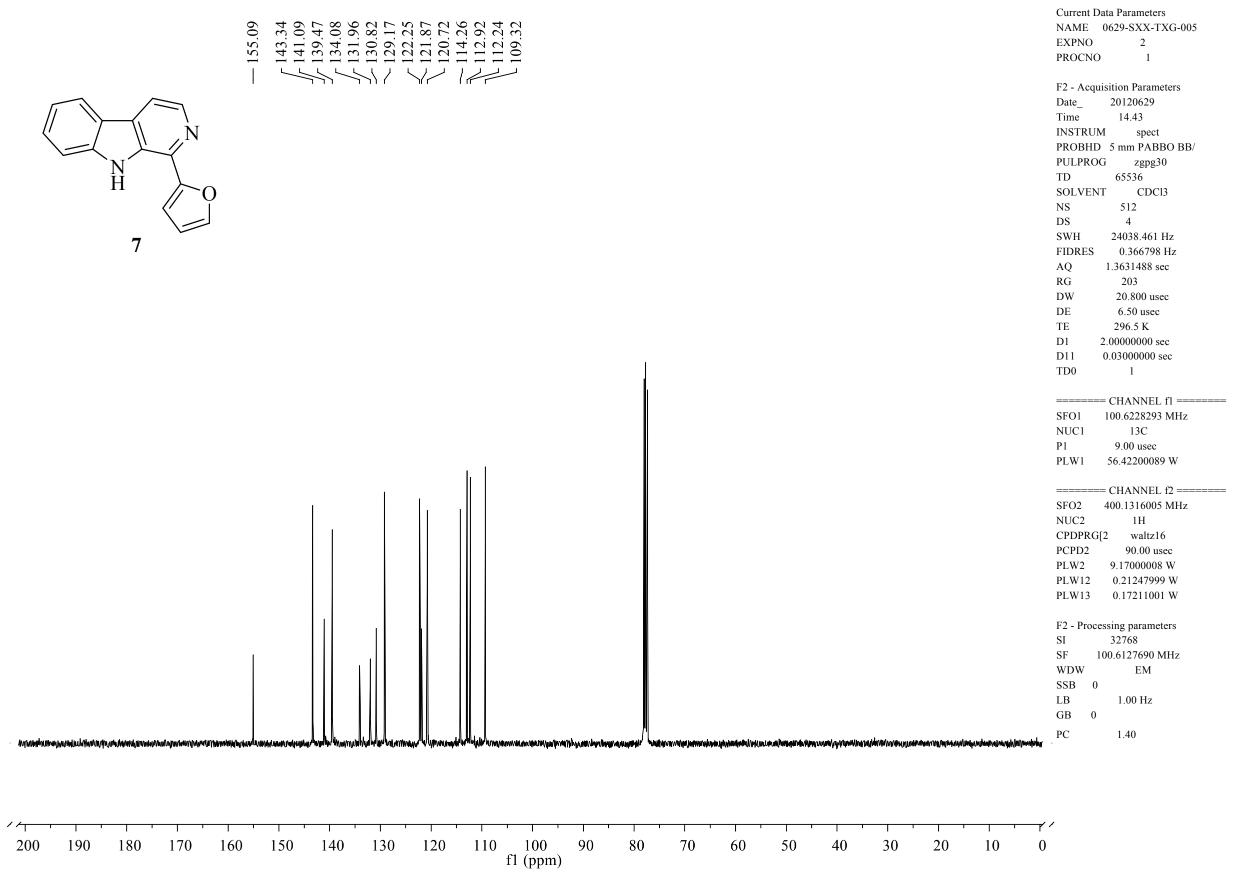

^{1}H NMR (DMSO-d_6, 400 MHz) spectrum of compound **8**:

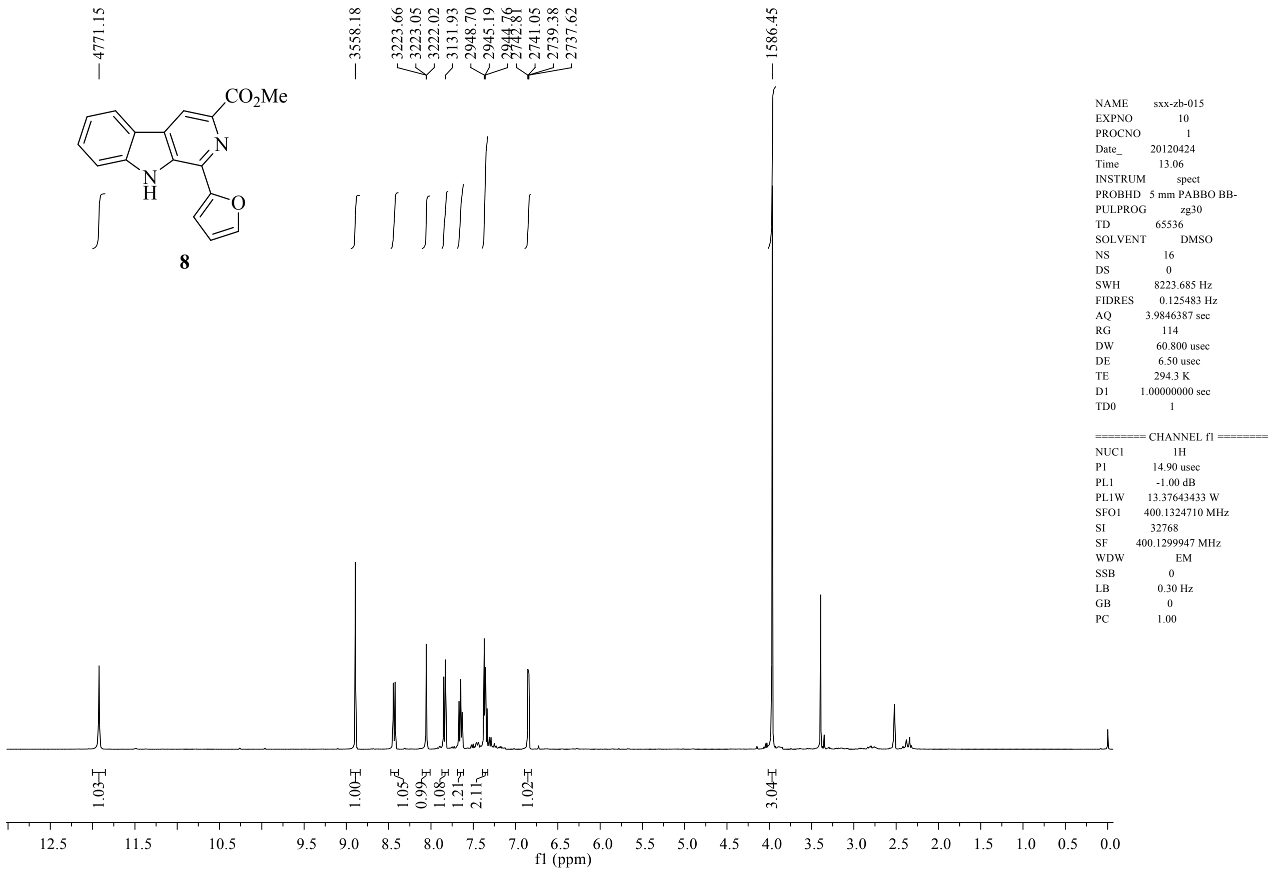

^{13}C NMR (DMSO-d_6, 100 MHz) spectrum of compound **8**:

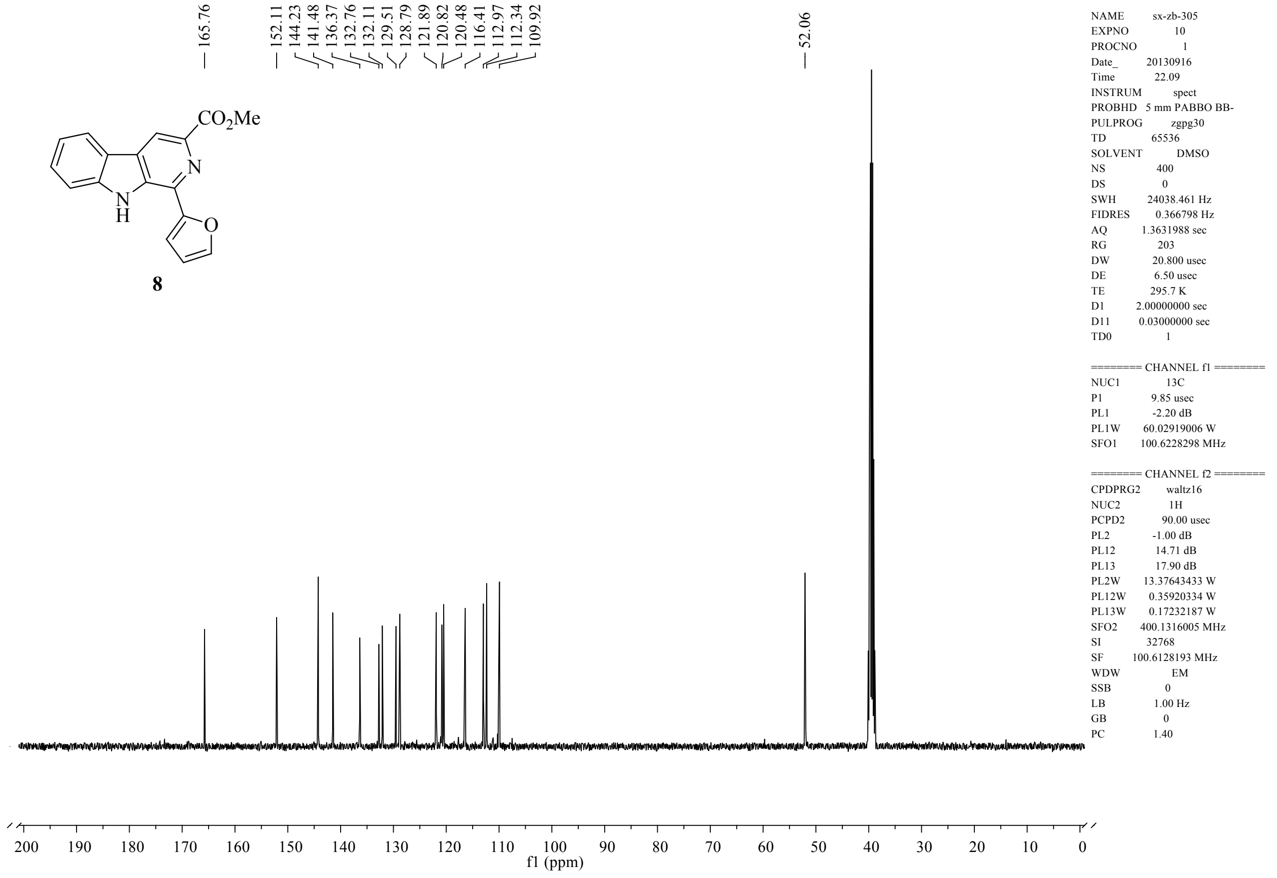

^{1}H NMR (DMSO-d_6, 400 MHz) spectrum of compound **9**:

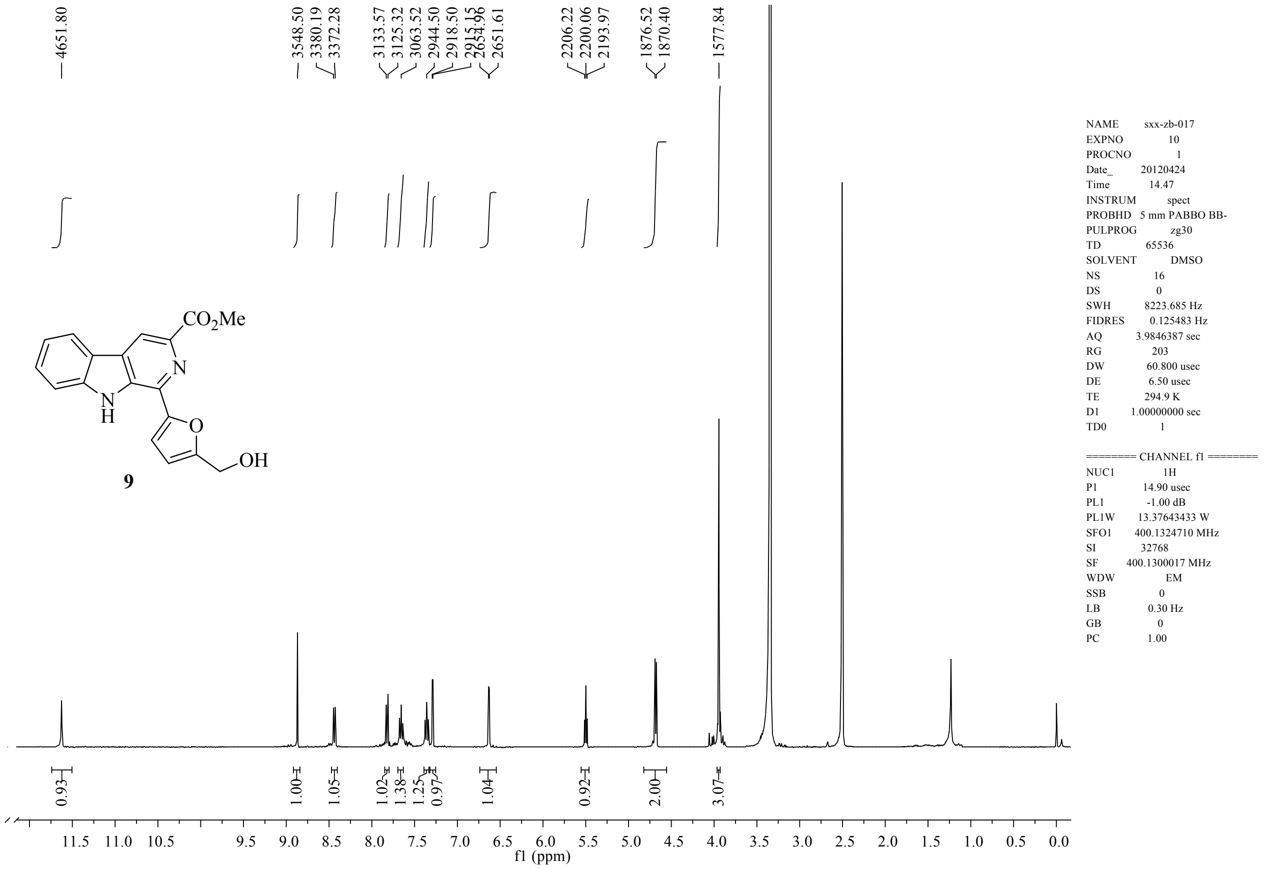

^{13}C NMR (DMSO-d_6, 100 MHz) spectrum of compound **9**:

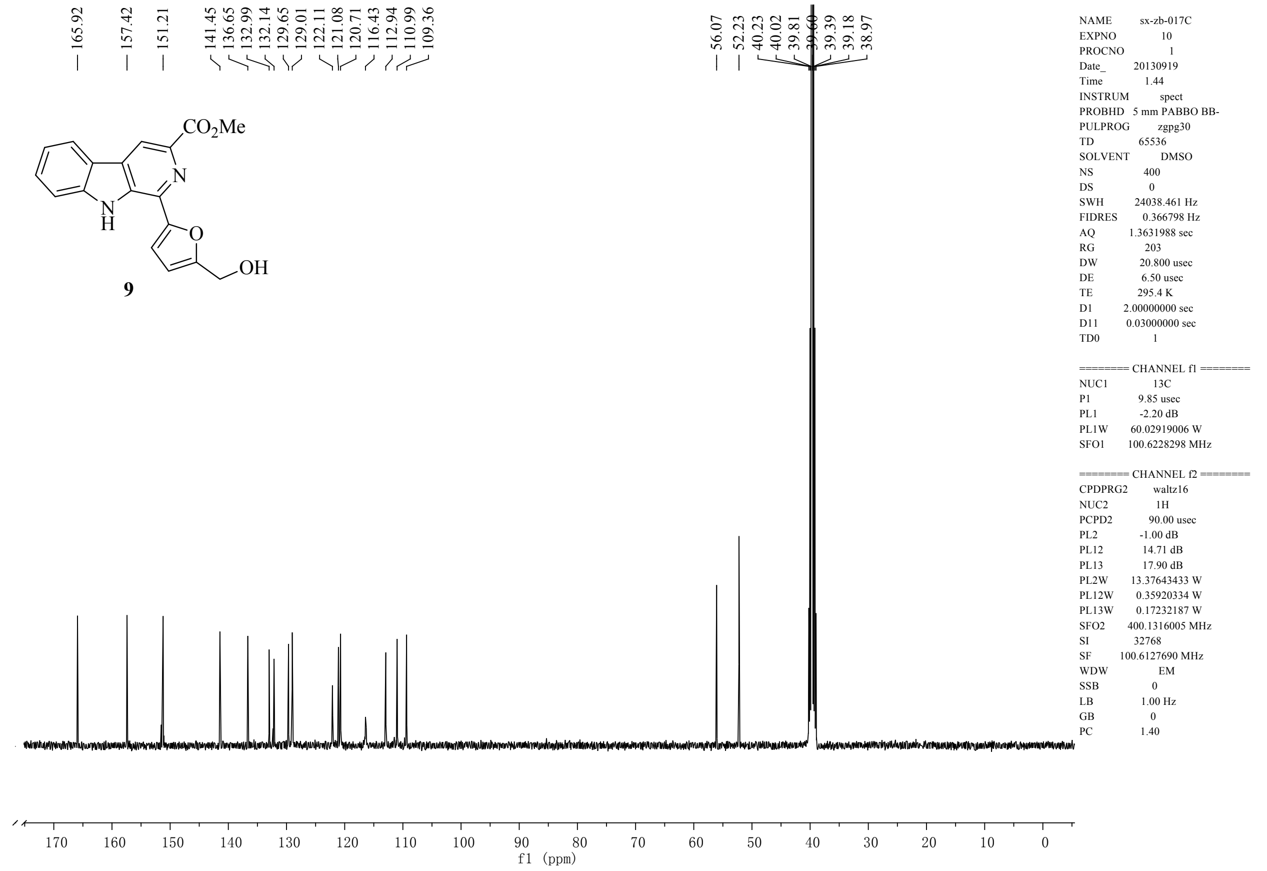

Supplement: RA-008-C7RA13434G-s001 [file RA-008-C7RA13434G-s001.pdf]
